# Systems Pharmacology Dissection of Multi-Scale Mechanisms of Action for Herbal Medicines in Stroke Treatment and Prevention

**DOI:** 10.1371/journal.pone.0102506

**Published:** 2014-08-05

**Authors:** Jingxiao Zhang, Yan Li, Xuetong Chen, Yanqiu Pan, Shuwei Zhang, Yonghua Wang

**Affiliations:** 1 Key laboratory of Industrial Ecology and Environmental Engineering (MOE), Faculty of Chemical, Environmental and Biological Science and Technology, Dalian University of Technology, Dalian, Liaoning, China; 2 College of Life Sciences, Northwest A & F University, Yangling, Shaanxi, China; 3 Center of Bioinformatics, Northwest A & F University, Yangling, Shaanxi, China; Sudbury Regional Hospital, Canada

## Abstract

Annually, tens of millions of first-ever strokes occur in the world; however, currently there is lack of effective and widely applicable pharmacological treatments for stroke patients. Herbal medicines, characterized as multi-constituent, multi-target and multi-effect, have been acknowledged with conspicuous effects in treating stroke, and attract extensive interest of researchers although the mechanism of action is yet unclear. In this work, we introduce an innovative systems-pharmacology method that combines pharmacokinetic prescreening, target fishing and network analysis to decipher the mechanisms of action of 10 herbal medicines like *Salvia miltiorrhizae*, *Ginkgo biloba* and *Ephedrae herba* which are efficient in stroke treatment and prevention. Our systematic analysis results display that, in these anti-stroke herbal medicines, 168 out of 1285 constituents with the favorable pharmacokinetic profiles might be implicated in stroke therapy, and the systematic use of these compounds probably acts through multiple mechanisms to synergistically benefit patients with stroke, which can roughly be classified as preventing ischemic inflammatory response, scavenging free radicals and inhibiting neuronal apoptosis against ischemic cerebral damage, as well as exhibiting lipid-lowering, anti-diabetic, anti-thrombotic and antiplatelet effects to decrease recurrent strokes. Relying on systems biology-based analysis, we speculate that herbal medicines, being characterized as the classical combination therapies, might be not only engaged in multiple mechanisms of action to synergistically improve the stroke outcomes, but also might be participated in reducing the risk factors for recurrent strokes.

## Introduction

Stroke is the second leading cause of death and the main cause of long-term disability in the world population. Annually, approximately 16 million first-ever strokes occur in the world, which results in nearly 6.2 million deaths [1]. And medication treatments both for acute stroke treatment and stroke prevention have changed over the years. Specifically, pharmacological treatments that are for the purposes of lysing clots and reestablishing blood flow, as well as those remedies that suppress apoptosis cascades after hypoxia-ischemia, inhibit ischemic inflammatory responses, prohibit excitatory neurotransmission or scavenge free radicals have all shown promising therapeutic potentials against stroke in animal models [2]. Meanwhile, preventive measures that aimed at controlling hypertension, atherosclerosis, hyperlipidemia, hyperglycemia, and other high risk factors can effectively reduce the incidence of stroke, as well as prevent recurrent stroke [3].

Researches show that cerebral ischemia can trigger an intricate series of biochemical and molecular mechanisms to impair the neurologic functions [4]; therefore, comparing with monotherapy, combination therapies have been identified as more promising strategies to improve stroke management [5]. Factually, more and more preclinical observations manifest that combining neuroprotective therapy with thrombolytic drugs is optimal, and this combination not only decreases reperfusion damage, but also inhibits downstream cascades of cell death [6]. And numerous combination therapies producing synergistic or additive effects have been reported when thrombolysis was used in conjunction with neuroprotective agents including anti-oxidants [7], MMP inhibitors [8], anti-thrombotic agents [9]. Additionally, considering the fact that various pathways implicated in cell death are triggered by cerebral ischemic, effective neuroprotective therapy might also require the combination of drugs in series which disturb distinct pathways during the evolution of ischemic damage [6]. Similarly, for stroke prevention, research shows that a combination strategy might reduce recurrent vascular events by 80% in patients with cerebrovascular disease [7]. Besides, the combinative therapy might reduce dosages for each agent, thereby decreasing the occurrence of adverse effects. However, in spite of these therapeutic benefits, effective and widely applicable medication treatments for stroke patients are still scarce.

Herbal medicines always contain combinations of bioactive ingredients which provide the synergistic effects, and thus have attracted more attentions in recent years. Fortunately, a large number of herbs have been widely used against cerebral stroke including *Salvia miltiorrhizae*, *Ginkgo biloba*, *Ephedrae herba*, *Erigeron breviscapus*, and so forth. Pharmacological studies have suggested that these herbal medicines or their corresponding products might dilate cardiocerebral vessels, suppress platelet aggregation, improve microcirculation in brain, protect against ischemic and reperfusion injury, possess neuroprotective properties or enhance the tolerance of ischemic tissue to hypoxia [10], [11]. However, unlike conventional pharmacological drugs used in western medicine, bioactive ingredients of medicinal herbs often have not been specified and measured, although there have been some attempts to standardize these medicines by some governments. Meanwhile, the multiple components, targets and pathways involved in herbal medicines also complicate the pharmacological research.

Being an emerging area of pharmacology, systems pharmacology combines pharmacokinetic (the absorption, distribution, metabolism, excretion and toxicity properties of drugs) and pharmacodynamics models, as well as pathway and network analyses, to systematically analyze drugs, drug targets and effects [12], which provides a platform for identifying multi-scale mechanisms of action of herbal medicines. Among them, the study of pharmacokinetic characteristics of herbal medicines might help us to understand the molecular mechanisms of herbal active compounds. Considering the complexity of multiple constituents and targets involved in the therapeutic properties of herbal medicines, analyzing botanical herbs in the context of biological pathways and networks can facilitate a better understanding of multiple mechanisms of action of herbal medicines. In previous work, we have constructed a systems-pharmacology-based method which is specially designed for herbal medicines in drug discovery and in deciphering the therapeutic mechanisms. Combining with pharmacokinetics, pharmacology and network analyses, this method is devoted to evaluate the therapeutic effectiveness of herbs through identifying their active constituents and possible targets, and has been successfully used in dissecting the therapeutic mechanisms of herbal medicine in treatment of cardiovascular diseases [13] and influenza [14].

In this text, a modified systems-pharmacology method is employed to dissect the multiscale mechanisms of action of herbal medicine for improving stroke management, including providing pharmacological effects against cerebral stroke and offering preventive measures to reduce the primary and secondary stroke. Specifically, we firstly explored anti-stroke herbs and their corresponding constituents with a wide-scale text mining method. Then, based on the pharmacokinetic principles of drugs, we employed six ADME profiles (including aqueous solubility, lipophilicity, drug-likeness, oral bioavailability, Caco-2 permeability and blood-brain barrier (BBB)) to filter active ingredients with favorable pharmacokinetic properties from these anti-stroke herbs. Subsequently, target proteins of these active compounds were identified and validated through a systematic approach which effectively integrated abundant biological and pharmacological methods. Finally, based on pharmacology and network analyses, we interpreted the multi-scale mechanisms of action of herbs in stroke prevention and management.

## Materials and Methods

### Constructing herbs and ingredients database for stroke

We conducted a robust wide-scale text mining of PubMed and the clinical trial database (www.clinicaltrials.gov), and extracted the available anti-stroke herbs manually using the ‘herbal medicine’ and ‘stroke’ as search terms. Owning to diverse herbs with different research extents, *P*-value (as displayed in Eq.1) which had been described in our previous work [15] was employed to equilibrate this bias and further appraise the chance probability of co-occurrence of each herb and stroke [16].
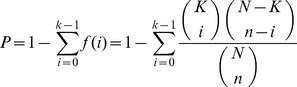
(1)where *N* represents the total number of papers in PubMed (22.8 million articles, until July 25th, 2013), *K* is the number of articles linked with stroke in PubMed (185,188 papers), *n* shows the number of articles of one herbal medicine, and *k* displays the number of articles about the effects of corresponding herb on stroke. Here, when *P*-value is less than 0.01, this herbal medicine is regarded as having significant correlation with stroke.

Subsequently, all constituents of these anti-stroke herbs were extracted from our own Traditional Chinese Medicine System Pharmacology Database and Analysis Platform (TCMSP, http://tcmspnw.com). Considering the fact that glycosides could be hydrolyzed to their aglycone forms before being absorbed, in this section, their corresponding aglycones were also added to the database for further research.

### ADME screening

According to the pharmacokinetic models constructed in drug design, ADME properties can be roughly classified into two categories: “physicochemical” and “physiological” groups. The physicochemical ADME features like aqueous solubility (S) and lipophilicity are ruled by simple physical and chemical laws, while the physiological properties including *in vivo* pharmacokinetic properties (like Caco-2 permeability, et al) and *in vitro* ADME features (such as oral bioavailability (OB), etc.) are regulated by several physiological elements [17]. In this text, for filtering active compounds with favorable pharmacokinetic properties, six *in silico* physicochemical or physiological predictive profiles were considered, which consist of aqueous solubility (log*S*, the logarithm of aqueous solubility), lipophilicity (log*P*, logarithm of octanol-water partition coefficient), drug-likeness (DL), oral bioavailability, Caco-2 permeability and BBB penetration.

#### Aqueous solubility

Being one of the chief physicochemical properties to be optimized in drug discovery, aqueous solubility has been considered as an important factor in drug absorption and distribution. And one drug with adequate aqueous solubility is considered exempt from bioavailability problems [18]. Here, for filtering compounds with suitable aqueous solubilities from herbs, we calculated the log*S* of each molecule using the ALOGPS 2.1 program [19]. This model was developed with 1291 molecules using ANN methodology and electrotopological state descriptors, resulting in an optimal predictive model with determination coefficient (*R* ^2^) = 0.91 and root mean squared error (*RMS*) = 0.62. Considering the fact that most drugs reveal a compromise between the polarities required for reasonable aqueous solubility and the hydrophobicities needed for satisfactory membrane passage [20], we set the threshold of log*S* in the range of −5 to −1.

#### Lipophilicity

Molecular lipophilicity, another basic physicochemical property, plays an essential role in determining ADME properties and the overall suitability of drug candidates, hence controlling molecular lipophilic property within an optimal range can improve compound quality and the likelihood of therapeutic success [21]. In this text, molecular lipophilicity (expressed as a value of log*P*) was also predicted by ALOGPS 2.1 program, which developed the predictive model of lipophilicity on the basis of 64 neural networks, 12,908 compounds with experimental log*P* values from PHYSPROP database and 75 input parameters, providing an optimal model with *RMS* = 0.49 and standard mean error = 0.26 [22]. On the basis of Lipinski's rule of five, the compounds with log*P* less than 5 were selected for further analysis.

#### Drug-likeness

For the reason that medicinal properties of herbs depend on the presence of active constituents with drug-likeness features, in this study, we employed a robust self-constructed model preDruglike as described in our previous work [23] to calculate the drug-likeness index of each compound in herbs. This model is constructed based on the molecular descriptors and Tanimoto coefficient (as displayed in Eq.2).
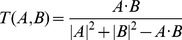
(2)where A shows the molecular properties of herbal ingredients, and B displays the average molecular properties of molecules in DrugBank database (http://www.drugbank.ca/) based on Dragon soft descriptors. Considering the fact that the average DL index for all 6511 molecules in DrugBank is 0.18, the molecule with suitable drug-likeness index (DL≥0.18) was chosen as candidate molecule for further research.

#### Oral bioavailability

Being an essential parameter in drug screening cascades, oral bioavailability was employed to determine the fraction of oral dose of compounds reaching systemic circulation in the TCM treatment. Here, a reliable *in silico* screening model OBioavail 1.1 constructed in our previous work [24] was engaged in OB value calculation of these herbal constituents. This model was constructed based on 805 structurally diverse drugs and drug-like molecules. Multiple linear regression, partial least square and support vector machine methods were employed during this model building, ending up with determination coefficient (*R* ^2^) = 0.80 and standard error of estimate (*SEE*) = 0.31 for test sets. Finally, the threshold of OB value is set to 30% by careful consideration of the following rules: 1) extracting information from the studied herbs should be as much as possible using the least number of molecules [13]; 2) the acquired model can be reasonably explicated by the existing pharmacological data [14].

#### Caco-2 permeability

For an orally administered drug, the majority of drug absorption occurs in the small intestine where the presence of villi and microvilli greatly increases the surface available for absorption [25]. A number of *in silico* drug absorption models using *in vitro* Caco-2 permeability have been widely established and used in drug discovery and development processes [26]. Here, we employed a robust *in silico* Caco-2 permeability prediction model preCaco2 [27] which was constructed by 100 drug molecules with satisfactory statistical results (*R* ^2^>0.8) to predict the drug absorption. Finally, we set the threshold of Caco-2 permeability to −0.4, for the reason that compound with Caco-2 value less than −0.4 is not permeable.

#### BBB penetration

Since blood-brain barrier is a protective fence between central nervous system (CNS) and systemic circulation to maintain the normal homeostasis of the CNS [28], predicting BBB permeability is regarded as an indispensible parameter in screening candidate compounds for stroke management. In this section, an updated and reliable BBB model preBBB which was constructed in our previous work [29] was employed to examine whether the constituents of herbal medicines pass through the BBB. The dataset of this model was composed of 190 related but chemically diverse compounds which are either penetrating or non-penetrating cross the BBB [23]. The statistical model was constructed by partial least squares discriminant analysis with two significant latent variables. In this model, compounds possessing BBB values greater than −0.3 were considered as penetrating, thus the threshold of BBB is set to −0.3.

### Target fishing and validation

To identify molecular targets of these active ingredients, we proposed a systematic approach which effectively integrated data mining, chemogenomic, pharmacological and statistical methods. To be specific, first of all, information of target proteins for herbal ingredients was identified from Therapeutic Target Database (TTD, http://bidd.nus.edu.sg/group/ttd/) [30], DrugBank and HIT (Herbal Ingredients' Targets Database, http://lifecenter.sgst.cn/hit/) [31], and all compound-target interactions from these databases were known and supported by published literatures. Secondly, the efficient systemic analysis methods, including Similarity Ensemble Approach (SEA, http://sea.bkslab.org/) [32], information integration method (STITCH, http://stitch.embl.de/) [33] and omics-based Ligand-Target Chemogenomic model (LTC) [34], were implemented to predict the potential target proteins of herbal ingredients. Finally, for better defining the role of herbs in stroke prevention and treatment, TTD, PharmGKB (http://www.pharmgkb.org) [35] and Comparative Toxicogenomics Database (CTD, http://ctdbase.org/) were employed to eliminate the noise in the two previous steps, providing a more complete and greater accuracy view on compound-target associations.

For improving the reliability of predicted target results, molecular docking program was employed in this section using GOLD5.1 software, which utilizes an evolutionary genetic algorithm to optimize the docked pose of the ligand within the receptor [36]. 3D crystallographic structures of these targets were downloaded from the RCSB Protein Data Bank (http://www.pdb.org/) or constructed by the Swiss-Model Automated Mode Serve (http://swissmodel.expasy.org/) if the 3D structures were not obtainable. Prior to performing the docking process, crystallographic ligands were extracted and mixed into docking database for re-dock, and hydrogen atoms were added. The default genetic algorithm parameters were used and *GoldScore* scoring function was selected. Eventually, the predicted compound-target interactions with Gold dock scores greater than 40 were taken into account for further research.

### Network construction

For further probing the multi-scale mechanisms of action of herbal medicines in stroke prevention and treatment, presently we constructed two kinds of networks: 1) Compound-Target network (C-T network). We used active compounds and their corresponding targets to generate a bipartite graph of compound-target interactions in which a compound and a target are linked with each other if the protein is a known or validated target of this compound, giving rise to the C-T network. 2) Target-Pathway network (T-P network). We firstly extracted the canonical pathways that were highly associated with stroke from KEGG database (http://www.genome.jp/kegg/), and then all target proteins attempted to be mapped onto these pathways, resulting in a target-pathway network. All these visualized networks were constructed by Cytoscape 2.8.1, an open source software project for biological network visualization and data integration [37].

## Results and Discussion

Extensive experiences and abundant clinical data reveal that, comparing with conventional FDA-approved drugs, medicinal herbs, featured as multiple constituents, targets and actions, have been reported exhibiting notable benefits in preventing and treating stroke although the mechanism of action remains unclear, which makes the efforts for deciphering the pharmacological mechanism of herbal medicines even more imperative [10]. Meanwhile, it is worth noting that, during the decoding process, there are several valuable resources available for drug development, such as discovering bioactive molecules with therapeutic effects in stroke, designing multi-target drugs or combination drugs, as well as expanding the structural diversity of small molecules for stroke. In this study, based on the application of bioinformatics resources and methodologies, we present a systematic analysis framework which integrates active ingredients filtering, target identification and network construction to decode the mechanisms of action of herbal medicines in stroke management and prevention.

### Anti-stroke herbs

Statistical results show that 10 herbs, as displayed in Table 1, are documented possessing significant correlations with stroke, like *S. miltiorrhiza*, *G. biloba*, *E. herba*, etc. Among them, *S. miltiorrhiza* obtains the optimal *P*-value (2.73E-16), indicating that anti-stroke may be one of the key therapeutic effects found so far for this herb. Factually, this herb has been documented exhibiting anti-atherosclerosis, anti-platelet aggregation, anti-oxidative and anti-inflammatory effects [38] which are all effective strategies in preventing and treating stroke. Analogously, *G. biloba* which possesses a favorable *P*-value (4.36E-15) has been widely used in treatment of acute ischemic stroke in China and occasionally in Europe [39].

**Table 1:** Statistics and association analysis between herbs and stroke.

Herb Name	*n*	*k*	*P*-value
*Salvia miltiorrhizae* (*S. miltiorrhiza*)	1716	55	2.73E-16
*Ginkgo biloba* (*G. biloba*)	2574	67	4.36E-15
*Ephedrae herba* (*E. herba*)	565	24	2.51E-10
*Gastrodia elata* (*G. elata*)	191	11	9.19E-07
*Panax notoginseng* (*P. notoginseng*)	581	19	9.45E-07
*Erigeron breviscapus* (*E. breviscapu*)	95	9	1.37E-07
*Rhizoma chuanxiong* (*R. chuanxiong*)	375	12	1.10E-04
*Panax ginseng* (*P. ginseng*)	3591	50	6.56E-04
*Uncaria rhynchophylla* (*U. rhynchophylla*)	102	6	2.48E-04
*Scutellaria baicalensis* (*S. baicalensis*)	848	15	6.89E-03

Surprisingly, further research exhibits that these 10 herbal medicines or their corresponding herbal constituents are widely participated in Traditional Chinese Patent Medicine (TCPM) for treating stroke in China. For instance, Dan Shen agents [11] (consisted by the main components of *S. miltiorrhiza*) with eligible clinical trials are commonly used in treating ischemic stroke through dilating cerebral vessel, suppressing aggregation of platelets, refining blood circulation, removing blood stasis, as well as defending against ischemic reperfusion injury [40]. Notably, the same situation occurs in Deng Zhan Xi Xin infection (the extractive of *E. breviscapu*), *Ginkgo biloba* agents and Ligustrazine injection (the major constituent of *R. chuanxiong*). Additionally, herbs including *G. elata*, *R. chuanxiong*, *P. notoginseng* and et al. are frequently applied as the major ingredients of Chinese medicinal formulae, such as Xiaoshuan Tongluo and She Xiang Kang Shuang Tablets, to against ischemia stroke, as well as hemiplegia and aphasia after stroke.

After eliminating the overlapped compounds among herbs, a total of 1285 compounds (as displayed in Table S1 in Supporting Information S1) are finally extracted from these 10 medicinal herbs, including 1234 herbal constituents and 51 aglycones (the hydrolysis products of 97 glycosides). Among them, *G. biloba* is found having the largest numbers of chemical components (256 molecules), following by *E. herba* with 236 chemicals and *S. miltiorrhiza* having 206 compounds.

### Screening active ingredients for anti-stroke

**Figure 1:**
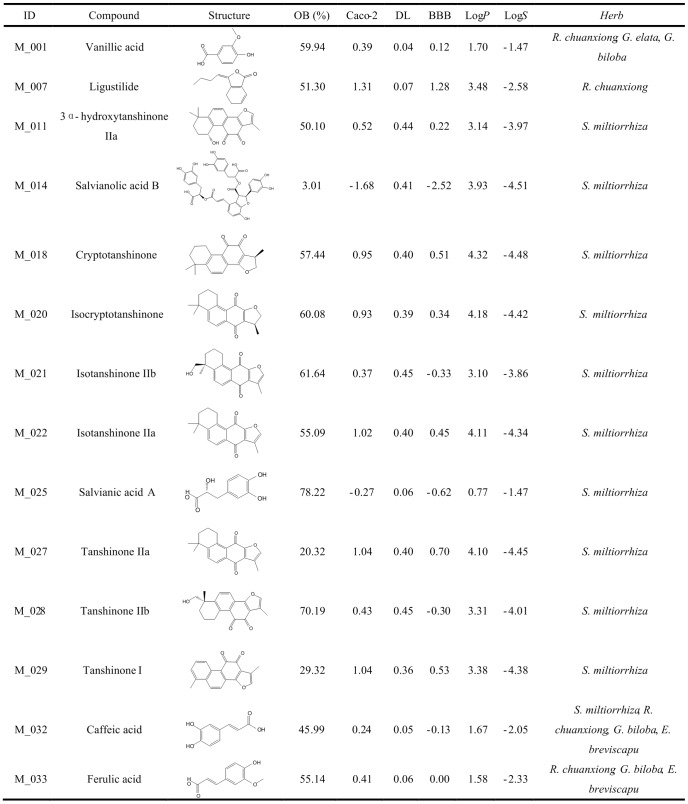
Representative active constituents of anti-stroke herbs and their corresponding ADME parameters (Part 1).

**Figure 2:**
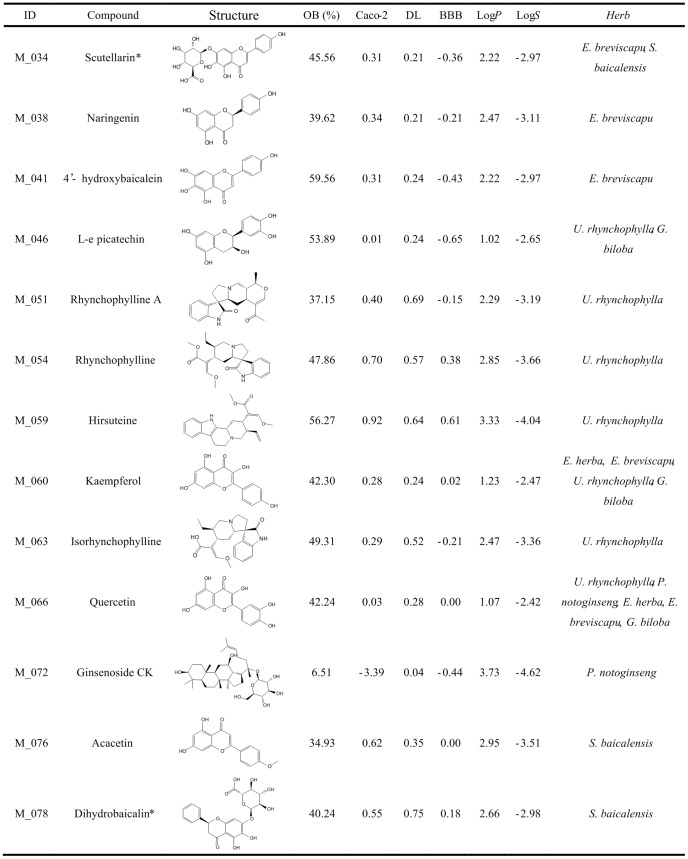
Representative active constituents of anti-stroke herbs and their corresponding ADME parameters (Part 2). The sign * represents the molecule after deglycosylation.

**Figure 3:**
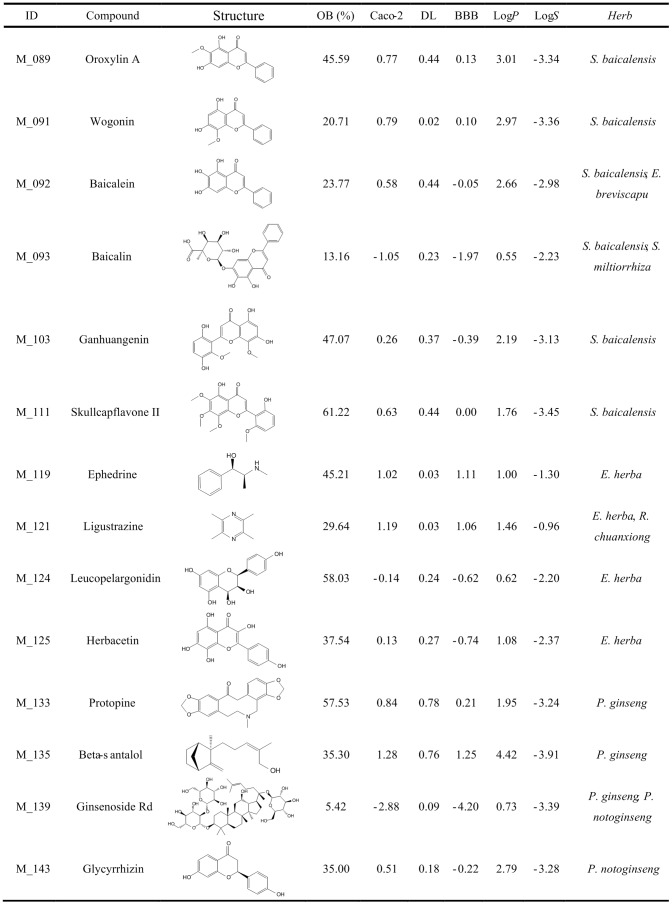
Representative active constituents of anti-stroke herbs and their corresponding ADME parameters (Part 3).

**Figure 4:**
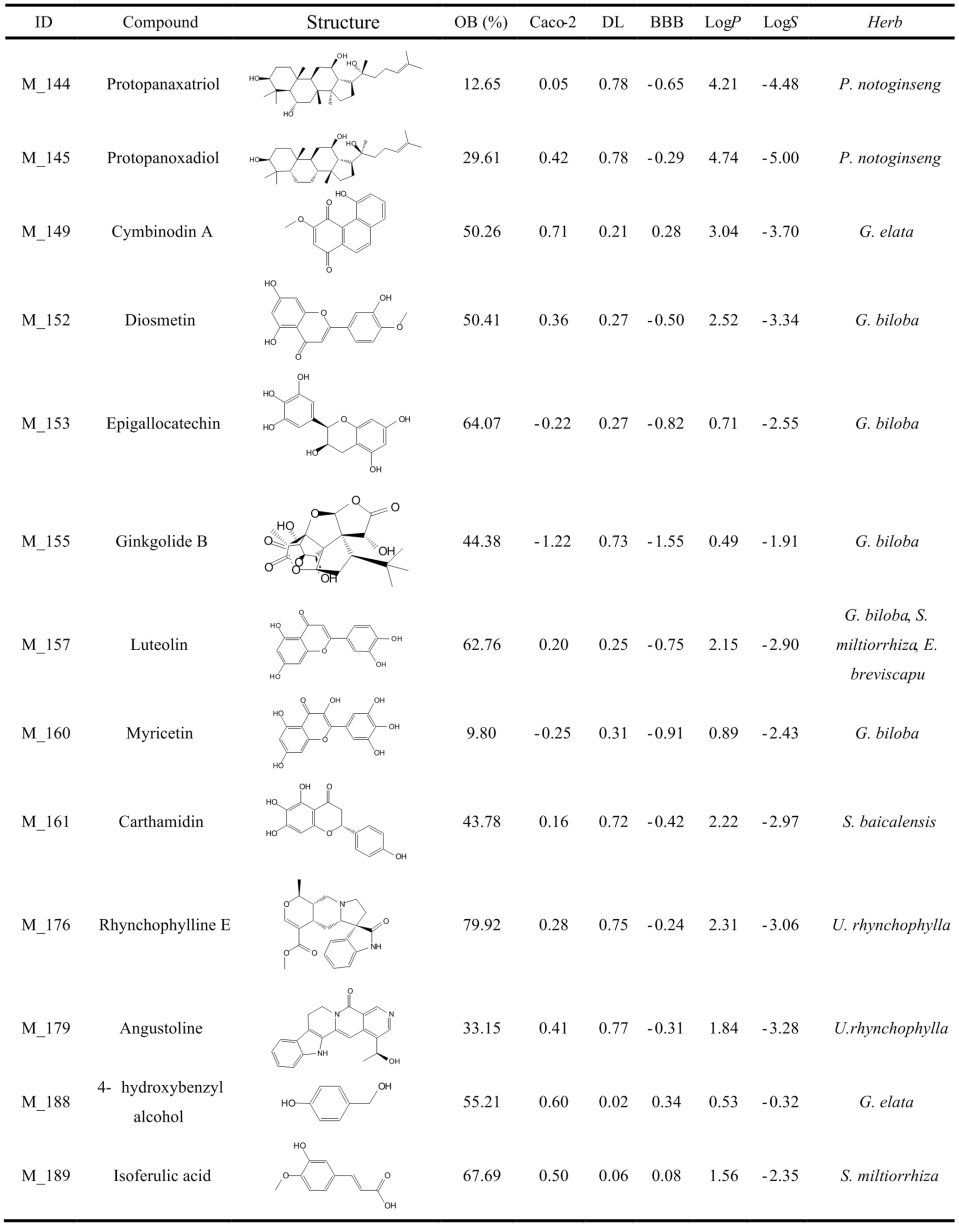
Representative active constituents of anti-stroke herbs and their corresponding ADME parameters (Part 4).

Before an orally administered drug exerting a pharmacological effect, this drug should hold satisfactory pharmacokinetic properties to overcome different barriers to reach its target. In this article, for screening the active pharmaceutical ingredients from these anti-stroke herbs, we introduced six ADME parameters: log*P*, log*S*, drug-likeness, Caco-2 cell permeability, oral bioavailability, blood-brain barrier permeability. As a result of these filters, 190 out of 1285 ingredients (as shown in Table S2 in Supporting Information S1) are demonstrated possessing satisfactory pharmacokinetic properties, among them, the representative compounds including their ADME parameters and structural information are displayed in Figures 1–4. Here, in order to illuminate clearly, three representative herbal medicines are specified in detail to interpret these filtering principles.

#### Uncaria rhynchophylla

In medicinal herb *U. rhynchophylla*, 41 out of 92 compounds are found meeting the screening criteria. Among them, plenty of indole alkaloids with satisfactory ADME properties have already been demonstrated exerting significant protective effects against several diseases. For example, the major constituents of this herb rhynchophylline (log*P* = 2.85, log*S* = −3.66, DL = 0.57, Caco-2 = 0.70, OB = 47.86% and BBB = 0.38) and isorhynchophylline (log*P* = 2.47, log*S* = −3.36, DL = 0.52, Caco-2 = 0.29, OB = 49.31% and BBB = −0.21) have been reported showing neuroprotective, vasodilatory and antiarrhythmia effects [41], [42], thus becoming the candidate compounds for treating cardiovascular and central nervous system diseases. Furthermore, isorhynchophylline also exhibits inhibitory effects on platelet aggregation and thrombosis, which mechanism may be at least partly due to the increase of intraplatelet cAMP generation [42], [43]. It is noteworthy that two bioactive compounds kaempferol (log*P* = 1.23, log*S* = −2.47, DL = 0.24, Caco-2 = 0.28, OB = 42.30% and BBB = 0.02) and quercetin (log*P* = 1.07, log*S* = −2.42, DL = 0.28, Caco-2 = 0.03, OB = 42.24% and BBB = 0.00), being the common flavones existed in several herbs like *U. rhynchophylla* and *G. biloba*, also show satisfactory pharmacokinetic properties.

As we know, unlike allopathic western medicine, herbal medicine characterized as holistic treatment emphasizes on maintaining the integrity of human body. Factually, medicinal herb *U. rhynchophylla* not only acts on the central nervous system to produce anti-inflammation, anti-apoptosis and free radical scavenging activities [44], but also exhibits antiplatelet and anti-hypertensive effects on the cardiovascular system [45]. These phenomena prove that, as a typical holistic therapy, herb *U. rhynchophylla*, besides providing benefits in stroke treatment, shows preventive effects in primary and secondary stroke prevention. Therefore, compounds which have suitable pharmacokinetic properties but low BBB values are also considered as potential active compounds, such as L-epicatechin (log*P* = 1.02, log*S* = −2.65, DL = 0.24, Caco-2 = 0.01, OB = 53.89% and BBB = −0.65) and angustoline (log*P* = 1.84, log*S* = −3.28, DL = 0.77, Caco-2 = 0.41, OB = 33.15% and BBB = −0.31).

#### Scutellaria baicalensis

For herb *S. baicalensis*, 56 out of 111 constituents pass through the filtering criteria, and the majority of them (46 out of 56 active compounds) are flavonoids, such as oroxylin A (log*P* = 3.01, log*S* = −3.34, DL = 0.44, Caco-2 = 0.77, OB = 45.59% and BBB = 0.13) and acacetin (log*P* = 2.95, log*S* = −3.51, DL = 0.35, Caco-2 = 0.62, OB = 34.93% and BBB = 0.00). Surprisingly, most flavonoids in this herb have been proven to possess various biological activities. For instance, oroxylin A shows memory ameliorative activity in memory impaired mice [46], and also has neuroprotective effect against ischemic/reperfusion-induced brain damage [47]; acacetin exhibits anti-neuroinflammation effect through regulating the response to LPS stimuli *in vitro* and *in vivo*, and is also considered as a potential therapeutic agent for brain diseases [48].

Besides these, those constituents which have high contents in herb but low OB indexes such as baicalin (OB = 13.16%), baicalein (OB = 23.77%) and wogonin (OB = 20.71%) should attract our more attention. For example, baicalin obtains a poor OB index, however, the content of this flavone in *S. baicalensis* is extremely high (8.12% of dry root mass) [48], which significantly increase its absolute OB value. Actually, baicalin has been documented exhibiting powerful pharmacological activities, including protective effect against cerebrovascular dysfunction and inhibitory effect on brain inflammatory response [49]. Therefore, these bioactive ingredients with high contents in herb are also added into active ingredients database, although they have low OB values.

#### Salvia miltiorrhiza

After ADME prescreening, 35 constituents from *S. miltiorrhiza* have desirable physicochemical and physiological properties, including many documented bioactive compounds such as cryptotanshinone (log*P* = 4.32, log*S* = −4.48, DL = 0.40, Caco-2 = 0.95, OB = 57.44% and BBB = 0.51) and tanshinone IIb (log*P* = 3.31, log*S* = −4.01, DL = 0.45, Caco-2 = 0.43, OB = 70.19% and BBB = −0.30) which have been reported to show neuroprotective [50], antidiabete [51], anti-atherosclerosis [52] and anti-inflammatory [53] effects. Two bioactive compounds tanshinone IIa and tanshinone I as the major constituents of *S. miltiorrhiza* are also put into the active ingredient database due to their *in vitro* and *in vivo* biological activities [54]. One exception is salvianolic acid B which obtains the low OB (3.01%), caco-2 (−1.68) and BBB (−2.52), however, several *in vitro* studies indicate that salvianolic acid B displays significant pharmacological activities: protection of local cerebral ischemia-reperfusion injury [55], inhibition of platelet aggregation [56] and low density lipoprotein oxidation [57], as well as improving regional cerebral blood flow. In fact, several investigations have proposed that the bioactive effects of salvianolic acid B may not only be due to itself but also its metabolites, like isoferulic acid (log*P* = 1.56, log*S* = −2.35, DL = 0.06, Caco-2 = 0.50, OB = 67.69% and BBB = 0.08) and salvianic acid A (log*P* = 0.77, log*S* = −1.47, DL = 0.06, Caco-2 = −0.27, OB = 78.22%, BBB = −0.62) [58], [13]. Therefore, these metabolites are also added into active ingredients database for further research.

### Target proteins of anti-stroke herbal ingredients

In order to further decipher the underlying molecular mechanism of these herbal medicines, target proteins of those active ingredients were identified based on the comprehensive method. As a result, 196 candidate targets are identified for 185 compounds, while other 5 ingredients have no related targets. For increasing the reliability of target fishing, the predicted compound-target interactions are validated using docking program. As displayed in the Table S3 in Supporting Information S1, 183 targets linked with 168 molecules are reserved. The results also display that the majority of active herbal ingredients (129 out of 168 molecules) are linked with more than one target, exhibiting their promiscuous actions. For instance, compound luteolin which is shared by herbs *S. miltiorrhiza*, *G. biloba* and *E. breviscapu* not only serves as an inhibitors of xanthine dehydrogenase/oxidase (XDH) [59] and interleukin-4 (IL4) [60], but also acts as an antagonist of peroxisome proliferator-activated receptor gamma (PPARG) [61]; flavonoid compound kaempferol (shared by four herbs like *E. herba*) has interactions with tens of target proteins like arachidonate 5-lipoxygenase (ALOX5), and the same phenomenon also occur in quercetin, another common flavonoid compound shared by five herbal medicines.

As we know, during cerebral ischemic injury, several mechanisms can lead to neurons damage such as oxidative and nitrosative stress, inflammation and apoptotic-like cell death [6]. Hence, enzyme systems like pro-inflammatory cascades, caspases, nitric oxide synthases (NOSs), superoxide dismutases (SODs) and matrix metalloproteinases (MMPs) all have the prospect of becoming the therapeutic targets for stroke. Additionally, owing to the fact that diabetes, hypertension, dyslipidemia and atherosclerosis are all the main risk factors of stroke, a modest controlling of these factors can significantly reduce the frequency of primary and secondary stroke. Thus, appropriate antihypertensive, lipid-lowering, anti-diabetic and antiplatelet therapies have the promise of developing into remedies for preventing stroke. Based on the above strategies, 94 targets (as displayed in Table 2–4) that are implicated in stroke prevention and treatment are retrieved from 183 target validated proteins; meanwhile 168 compounds linked with these 94 targets are also extracted for further analysis.

**Table 2:** The stroke-related targets of herbs and their corresponding diseases (Part 1).

ID	Protein Name	UniProt ID	Gene Name	Related Diseases
T_01	Tyrosine-protein phosphatase non-receptor type 1	P18031	PTPN1	Diabetes
**T_02**	**Prostaglandin G/H synthase 2**	**P35354**	**PTGS2**	**Stroke, Inflammation, Nervous system diseases, Myocardial infarction**
**T_03**	**Prostaglandin G/H synthase 1**	**P23219**	**PTGS1**	**Inflammation, Cardiovascular disease**
**T_04**	**Nitric-oxide synthase, endothelial**	**P29474**	**NOS3**	**Coronary artery disease, Heart diseases, Hypertension, Thromboembolism**
**T_05**	**Glycogen synthase kinase-3 beta**	**P49841**	**GSK3B**	**Diabetes, Ischemia, Brain injury, Nervous system diseases**
**T_06**	**Alpha-1D adrenergic receptor**	**P25100**	**ADRA1D**	**Hypertension**
**T_07**	**Nitric oxide synthase, inducible**	**P35228**	**NOS2**	**Ischemia reperfusion injury, Hypertension**
**T_08**	**Matrix metalloproteinase-9**	**P14780**	**MMP9**	**Atherosclerosis, Multiple sclerosis, Coronary artery disease, Heart failure**
T_09	Mitogen-activated protein kinase 1	P28482	MAPK1	Nervous system diseases
**T_10**	**Coagulation factor X**	**P00742**	**F10**	**Atrial fibrillation, Thromboembolism, Coronary artery disease**
T_11	Beta-1 adrenergic receptor	P08588	ADRB1	Hypertension
**T_12**	**Glycine receptor subunit alpha-1**	**P23415**	**GLRA1**	**Nervous system diseases**
**T_13**	**Superoxide dismutase [Cu-Zn]**	**P00441**	**SOD1**	**Ischemic injury**
**T_14**	**Tumor necrosis factor**	**P01375**	**TNF**	**Brain Diseases, Diabetes, Inflammation**
**T_15**	**Transcription factor p65**	**Q04206**	**RELA**	**Embolic focal cerebral ischemia, Ischemic renal injury, Thrombosis, Inflammation, Atherosclerosis**
T_16	Glycogen phosphorylase, muscle form	P11217	PYGM	Diabetes
**T_17**	**Coagulation factor VII**	**P08709**	**F7**	**Thromboembolism, Cardiovascular diseases**
**T_18**	**Caspase-3**	**P42574**	**CASP3**	**Venous thrombosis**
**T_19**	**Apoptosis regulator Bcl-2**	**P10415**	**BCL2**	**Nervous system diseases, Neoplasms**
**T_20**	**Apoptosis regulator BAX**	**Q07812**	**BAX**	**Neoplasms**
T_21	3-hydroxy-3-methylglutaryl-coenzyme A reductase	P04035	HMGCR	Myocardial infarction, Hyperlipidemias, Cardiovascular diseases, Arteriosclerosis, Hypertension
T_22	TGF-beta receptor type-1	P36897	TGFBR1	Cardiovascular diseases, Hypertension
T_23	Peroxisome proliferator-activated receptor gamma	P37231	PPARG	Hypertension, Cardiovascular diseases, Hyperlipidemias
T_24	Peroxisome proliferator-activated receptor delta	Q03181	PPARD	Venous thrombosis, Hyperlipidemias, Diabetes, Inflammation
T_25	Phospholipase A2, membrane associated	P14555	PLA2G2A	Myocardial infarction, Coronary artery disease, Atherosclerosis
T_26	Glucocorticoid receptor	P04150	NR3C1	Hypertension, Cardiovascular diseases, Diabetes

**Table 3:** The stroke-related targets of herbs and their corresponding diseases (Part 2).

ID	Protein Name	UniProt ID	Gene Name	Related Diseases
T_27	Mineralocorticoid receptor	P08235	NR3C2	Hypertension, Hyperlipidemias, Brain injury
**T_28**	**72 kDa type IV collagenase**	**P08253**	**MMP2**	**Nervous system diseases**
T_29	Thrombomodulin	P07204	THBD	Thrombosis
**T_30**	**E-selectin**	**P16581**	**SELE**	**Hypertension**
T_31	Protein kinase C, beta type	P05771	PRKCB	Diabetes
**T_32**	**Tissue-type plasminogen activator**	**P00750**	**PLAT**	**Nervous system diseases**
T_33	Vascular endothelial growth factor receptor 2	P35968	KDR	Hypertension
**T_34**	**Intercellular adhesion molecule 1**	**P05362**	**ICAM1**	**Multiple sclerosis, Inflammation**
**T_35**	**Prothrombin**	**P00734**	**F2**	**Myocardial infarction, Thromboembolism**
T_36	Cathepsin K	P43235	CTSK	Atherosclerosis
**T_37**	**BCL2-like 1**	**Q07817**	**BCL2L1**	**Neoplasms**
T_38	Oxysterols receptor LXR-alpha	Q13133	NR1H3	Cardiovascular diseases, Hypertension, Coronary artery disease, Atherosclerosis
T_39	Cell division protein kinase 4	P11802	CDK4	Diabetes
**T_40**	**Caspase-9**	**P55211**	**CASP9**	**Nervous system diseases**
T_41	Peroxisome proliferator activated receptor alpha	Q07869	PPARA	Hypertension, Coronary artery disease, Hyperlipidemias, Cardiovascular diseases
T_42	Thyroid hormone receptor beta-1	P10828	THRB	Hyperlipidemia
T_43	Transforming growth factor beta-1	P01137	TGFB1	Multiple sclerosis, Neoplasms
T_44	Integrin beta-3	P05106	ITGB3	Myocardial infarction, Cardiovascular disease
T_45	Endothelin-1 receptor	P25101	EDNRA	Hypertension, Cardiovascular disease
**T_46**	**Xanthine dehydrogenase**	**P47989**	**XDH**	**Hypertension**
**T_47**	**P-selectin**	**P16109**	**SELP**	**Stroke, Inflammation**
**T_48**	**Arachidonate 5-lipoxygenase**	**P09917**	**ALOX5**	**Cerebrovascular disorders, Inflammation**
T_49	Solute carrier family 2, facilitated glucose transporter member 4	P14672	SLC2A4	Diabetes
**T_50**	**Plasminogen activator inhibitor 1**	**P05121**	**SERPINE1**	**Thromboembolism, Inflammation**
T_51	Interstitial collagenase	P03956	MMP1	Myocardial infarction
T_52	Insulin receptor	P06213	INSR	Diabetes
**T_53**	**Interleukin-4**	**P05112**	**IL4**	**Inflammation**
**T_54**	**Interleukin-13**	**P35225**	**IL13**	**Inflammation**
T_55	Interferon gamma	P01579	IFNG	Multiple sclerosis
T_56	Heme oxygenase 1	P09601	HMOX1	Atherosclerosis, Cardiovascular diseases
**T_57**	**Hypoxia-inducible factor 1-alpha**	**Q16665**	**HIF1A**	**Stroke, Cardiovascular diseases**
T_58	Glucose-6-phosphatase	P35575	G6PC	Hyperglycemia
T_59	CD40 ligand	P29965	CD40LG	Atherosclerosis
**T_60**	**RAC-alpha serine/threonine-protein kinase**	**P31749**	**AKT1**	**Stroke, Brain ischemic insult, Diabetes**
**T_61**	**Monoamine oxidase A**	**P21397**	**MAOA**	**Nervous system diseases**
**T_62**	**Interleukin-6**	**P05231**	**IL6**	**Inflammation**
**T_63**	**C-C motif chemokine 2**	**P13500**	**CCL2**	**Atherosclerosis, Inflammation, Cardiovascular diseases**

**Table 4:** The stroke-related targets of herbs and their corresponding diseases (Part 3).

ID	Protein Name	UniProt ID	Gene Name	Related Diseases
T_64	Angiotensin-converting enzyme	P12821	ACE	Arteriosclerosis, Hypertension, Heart failure, Hypokinesia, Stroke, Thromboembolism
T_65	Alpha-2A adrenergic receptor	P08913	ADRA2A	Hypertension, Heart failure, Cardiovascular diseases
T_66	Estrogen receptor	P03372	ESR1	Hyperlipidemia, Coronary artery diseases
**T_67**	**5-hydroxytryptamine receptor 1D**	**P28221**	**HTR1D**	**Nervous system diseases**
**T_68**	**5-hydroxytryptamine receptor 2A**	**P28223**	**HTR2A**	**Nervous system diseases, Diabetes**
T_69	Phosphatidylinositol-4,5-bisphosphate 3-kinase catalytic subunit, gamma isoform	P48736	PIK3CG	Myocardial infarction, Cardiovascular diseases
**T_70**	**Poly [ADP-ribose] polymerase 1**	**P09874**	**PARP1**	**Cardiovascular diseases**
**T_71**	**Myeloperoxidase**	**P05164**	**MPO**	**Nervous system diseases**
**T_72**	**Interleukin-1 beta**	**P01584**	**IL1B**	**Inflammation**
**T_73**	**Tissue factor**	**P13726**	**F3**	**Myocardial ischemia**
**T_74**	**C-X-C motif chemokine 10**	**P02778**	**CXCL10**	**Inflammation**
**T_75**	**Caspase-8**	**Q14790**	**CASP8**	**Nervous system diseases**
**T_76**	**Interleukin-1**	**P22301**	**IL10**	**Inflammation**
**T_77**	**Monoamine oxidase B**	**P27338**	**MAOB**	**Nervous system diseases**
**T_78**	**Ornithine decarboxylase**	**P11926**	**ODC1**	**Inflammation**
**T_79**	**Adenosine receptor A2a**	**P29274**	**ADORA2A**	**Ischemia reperfusion injuries, Inflammation, Nervous system diseases**
**T_80**	**Mitogen-activated protein kinase 14**	**Q16539**	**MAPK14**	**Inflammation, Nervous system diseases**
**T_81**	**Macrophage migration inhibitory factor**	**P14174**	**MIF**	**Arteriosclerosis, Inflammation**
T_82	Sodium-dependent serotonin transporter	P31645	SLC6A4	Dyslipidemias, Nervous system diseases
T_83	Stromelysin-1	P08254	MMP3	Myocardial infarction, Nervous system diseases
T_84	Alpha-1A adrenergic receptor	P35348	ADRA1A	Hypertension
**T_85**	**Acetylcholinesterase**	**P22303**	**ACHE**	**Brain ischemia, Nervous system diseases**
**T_86**	**Beta-2 adrenergic receptor**	**P07550**	**ADRB2**	**Multiple sclerosis, Hypertension, Inflammation**
**T_87**	**Histamine H1 receptor**	**P35367**	**HRH1**	**Cardiovascular diseases, Nervous system diseases, Ischemia**
**T_88**	**D(2) dopamine receptor**	**P14416**	**DRD2**	**Nervous system diseases**
**T_89**	**Arachidonate 12-lipoxygenase**	**P1854**	**ALOX12**	**Nervous system diseases**
**T_90**	**Integrin beta-2**	**P05107**	**ITGB2**	**Ischemic stroke**
**T_91**	**Interleukin-8**	**P10145**	**IL8**	**Nervous system diseases, Inflammation**
**T_92**	**Gamma-aminobutyric-acid receptor subunit alpha-5**	**P31644**	**GABRA5**	**Central Nervous system diseases**
T_93	Acyl coenzyme A:cholesterol acyltransferase	P23141	CES1	Atherosclerosis, Dyslipidemias, Cardiovascular disease, Nervous system diseases
T_94	Fatty acid binding protein adipocyte	P159	FABP4	Atherosclerosis

For further elucidating the relationships between herbal ingredients and stroke, these 94 targets are sent to TTD and PharmGKB for discovering their corresponding diseases, which are then roughly classified into two categories: for stroke treatment (marked in bold in Tables 2–4) and for stroke prevention (others in the tables). As displayed in Tables 2–4, herbal medicines, featured as the typical multicomponent regimens, might simultaneously regulate multiple molecular components which are involved in the pathogenesis of stroke to exhibit the therapeutic or prophylactic effects. Factually, for stroke treatment and prevention, there exist many overlapping features in their pathologic and therapeutic processes. For example, inflammation involved in blood vessel wall and cerebral parenchyma contributes to tissue injury after ischemia and also to stroke risk [6]; recent observation confirms that a PPARG agonist pioglitazone being a prescription drug with hypoglycemic action to treat diabetes can decrease the incidence of stroke in patients with diabetes, and mediation of intracerebral PPARG might also provide neuroprotective effect against brain ischemic injury [62].

Specifically, as displayed in Tables 2 and 3, proteins like PTGS2, AKT1, NOS2, MMP9, SOD1 and CASP3 can be regulated by herbal ingredients, and then may produce anti-inflammatory, anti-oxidant and anti-apoptosis effects against ischemic cerebral damage. For example, target PTGS2 is documented to be involved in pathogenic events that take place in both the early and late stages of cerebral ischemia, including attenuation of glutamate neurotoxicity [63], and abrogation of deleterious effects of postischemic inflammation [64]. Therefore, inhibition of PTGS2 has developed into an attractive therapeutic strategy of stroke due to the following advantages: 1) targeting both “early” and “later” components of ischemic injury; 2) relatively safe and well tolerated. For stroke prevention, herbal constituents that interact with series of targets like ACE, PPARG, ESR1, NR3C1, ADRA2A and PYGM, might exhibit lipid-lowering, anti-diabetic, anti-thrombotic and antiplatelet effects, subsequently lowering the risk of stroke. For instance, epidemiological evidence shows that blood pressure level is directly and continuously related with the ischemic stroke and intracerebral hemorrhage [65], and recently, an ACE inhibitor perindopril (a well-established antihypertensive agent)-based therapy is reported to reduce the risks of both ischemia and hemorrhagic stroke [66].

### Network analysis of molecular mechanism for anti-stroke herbs

#### C-T network: elucidating the combining and additive strategies for herbal medications in stroke therapy and prophylaxis

**Figure 5:**
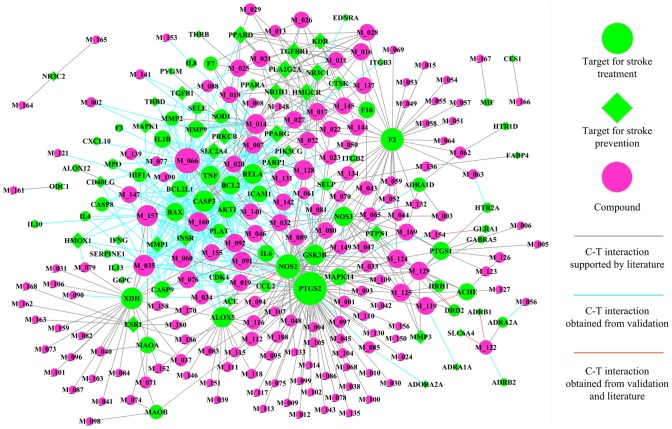
Compound-target network of anti-stroke herbs. Node size is proportional to its degree.

As displayed in Figure 5, C-T network is generated based on 641 associations between 168 herbal ingredients (magenta circle nodes) and their corresponding 94 targets. As outlined above, we distinguish the potential targets into stroke therapy (limegreen circle nodes) and prophylaxis (limegreen diamond ones). Network analysis results show that the average number of targets per compound is 3.9. Nearly two thirds compounds (105 out of 168 compounds) are linked with more than one target, showing the multi-target properties of herbal ingredients. Among them, compound quercetin possesses the highest number of target interactions (degree = 42), following by apigenin with 26 targets and luteolin having 25 linked targets.

**Figure 6:**
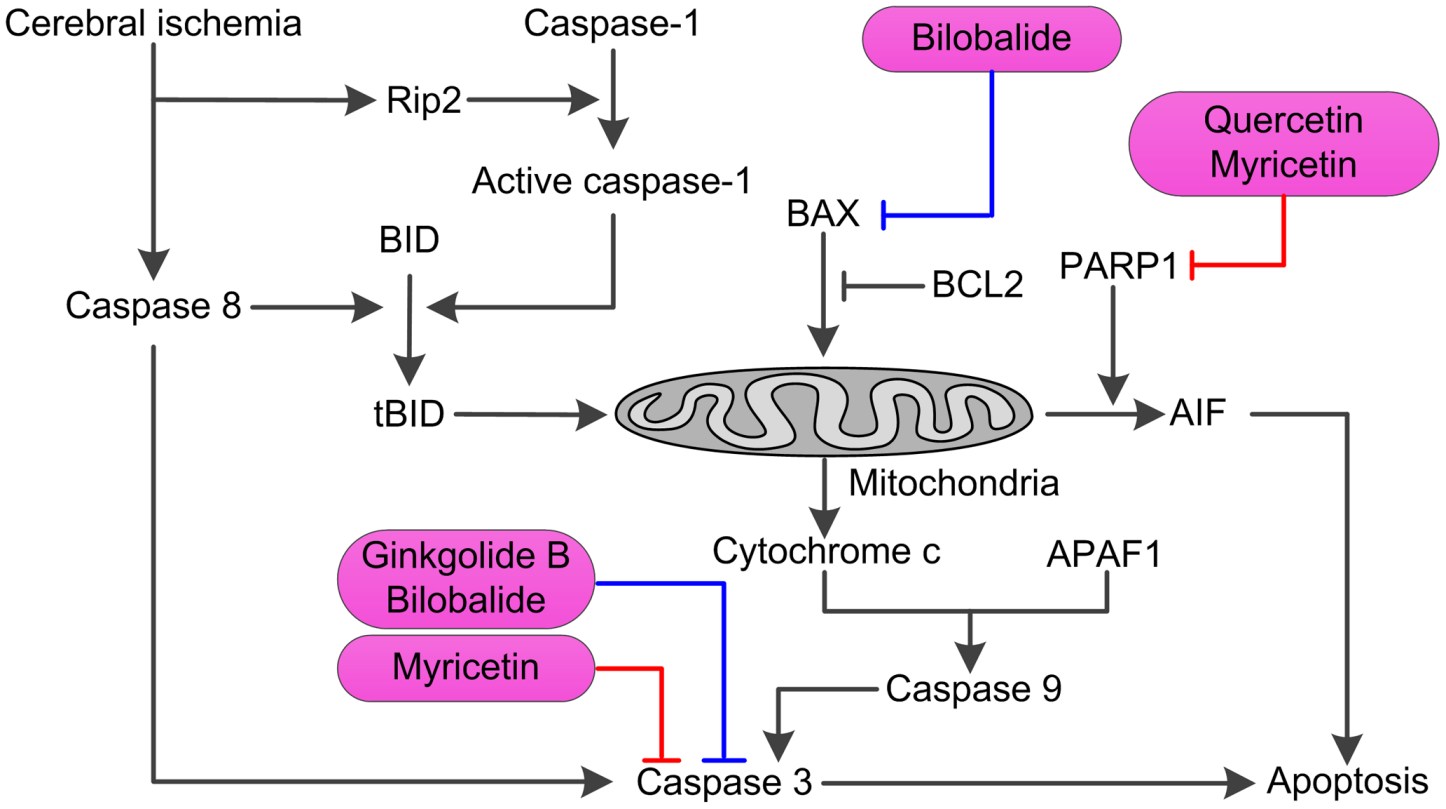
Illustrating the synergistic actions of anti-apoptosis of *Ginkgo biloba*.

As mentioned above, several pathways that lead to cell death are triggered by cerebral ischemia; therefore effective neuroprotective strategy might require a combination of drugs which act on distinct pathways during the progression of ischemic damage. Fortunately, by further analysis of the C-T net, we conclude that, in one herb, several compounds may bind to different points of same signaling pathway or different targets of different signaling cascades to exhibit the synergistic therapeutic actions. As an illustration, herb *Ginkgo biloba* (as displayed in Figure 6) was specified in detail to interpret the combination principle. During ischemia and reperfusion injury, overactivation of PPAR1 can induce translocation of AIF (apoptosis-inducing factor) from mitochondria to nucleus, which results in subsequent cell death [67]. Fortunately, compounds quercetin and myricetin of *G. biloba* which are identified as significant inhibitors of PPAR1 [68] collectively mediate the caspase-independent pathway, and have the potential to exhibit the additive anti-apoptosis effects. Moreover, compounds ginkgolide B and bilobalide of this herb can block neuronal apoptosis by attenuating the activation of caspase-3 [69]; meanwhile myricetin can directly bind to the active site of caspase-3 to inhibit its activity [70]. All of these document that this herb is possible to regulate the caspase-independent pathway to display the synergistic inhibition of neuronal cell apoptosis. Therefore, we speculate that herb *G. biloba* might provide an effective therapeutic approach for the treatment of cerebral ischemia through synergistic inhibition of both the caspase-dependent and independent pathways.

Further analysis of the C-T net shows that, besides those combination therapies, several active ingredients in one herb can act on one common target, which might exhibit additive effects for improving the stroke outcome. For instance, after ischemia develops, NOS2 which produces NO contributes to the evolution of the cerebral injury [71], because of the fact that NOS2 is expressed in the setting of the inflammation response after cerebral ischemia. Then, inhibition of NOS2 expression or activity has potential to develop into an attractive therapeutic strategy for stroke. Fortunately, 39 herbal ingredients (as shown in Figure 5) like kaempferol, quercetin, ginkgolide B, ginsenoside C-K and oroxylin A all have interactions with NOS2, and might contribute to the anti-stroke effect of herbal medicines. Just for herb *S. baicalensis*, 12 constituents such as oroxylin A, acacetin and eugenol work together on protein NOS2, and might provide additive effects to reduce the infarct volume and ischemia brain damage.

Owing to the pathophysiology of stroke involved in multiple mechanisms, the use of combinations for improving the stroke outcome is especially rational. Facts have proven that therapies that eliminate the clot, restore blood flow, inhibit excitatory neurotransmission, reduce inflammatory response following ischemia/reperfusion, or scavenge free radicals all have promising therapeutic potentials in animal models of stroke [72], [73]. Luckily, in the C-T network of anti-stroke herbs (Figure 5), several high degrees of correlations of targets for anti-inflammation (like NOS2, ALOX5 and PPARG), anti-oxidant (including XDH and HMOX1) and anti-apoptosis (such as BAX, BCL2 and caspse-3), neuroprotection (for instance: AKT1, MMP9 and MMP2) are regulated by herbal ingredients, which might display a combination of multiple mechanisms for effective long-term treatment for stroke patients.

Furthermore, as we know, the incidence of stroke can be reduced with appropriate preventive measures like blood pressure lowering, as well as lipid-lowering, anti-diabetic, anti-thrombotic and antiplatelet therapies. In the C-T network of Figure 5, besides those potential therapies for stroke treatment, series of target proteins of herbal constituents are also engaged in diabetes mellitus (such as PPARG and NR3C1), atherosclerosis (for instance: PLA2G2A, NR1H3 and F10), hypertension (including ADRB1, NOS3 and ACE), dyslipidemia (like NR1H2, PPARD and HMGCR), which may be implicated in the primary and secondary stroke preventions.

In summary, as displayed in the C-T net, botanical medicines are engaged in multiple mechanisms of stroke to synergistically improve outcome, which are roughly classified into preventing inflammatory response, suppressing apoptosis, scavenging free radicals, inhibition of platelet aggregation, as well as modifying diabetes mellitus, hypertension, dyslipidemia and other stroke risk factors. And several ingredients in one herb might act on one the same target to display an additive effect, or bind at the different points of the same signaling pathway or at different targets of several pathways, and then have the potential to exhibit the synergistic therapeutic actions for stroke therapy and prophylaxis.

#### T-P network: holistic mechanisms of anti-stroke medicinal herbs

For better elaborating the major pathways involved in herbal medicines for stroke therapy, we extract the canonical pathways that are highly associated with stroke from KEGG database (http://www.genome.jp/kegg/), resulting in 30 canonical pathways including PI3K-Akt signaling pathway, TNF signaling pathway, neuroactive ligand-receptor interaction, calcium signaling pathway and metabolic pathways. For example, being a central mediator in signal transduction pathways modulating cell growth, metabolism and survival, the PI3K-Akt signaling pathway can mediate the neuroprotective activity of vascular endothelial growth factor and induce BBB permeability after focal cerebral ischemia [74]; MAPK signaling pathway transduces a large number of external signals, resulting in a large-scale cellular responses which contain cell proliferation, differentiation, inflammation and apoptosis, and now it is becoming obvious that this pathway plays a significant role in mediating cell survival following brain ischemia.

**Figure 7:**
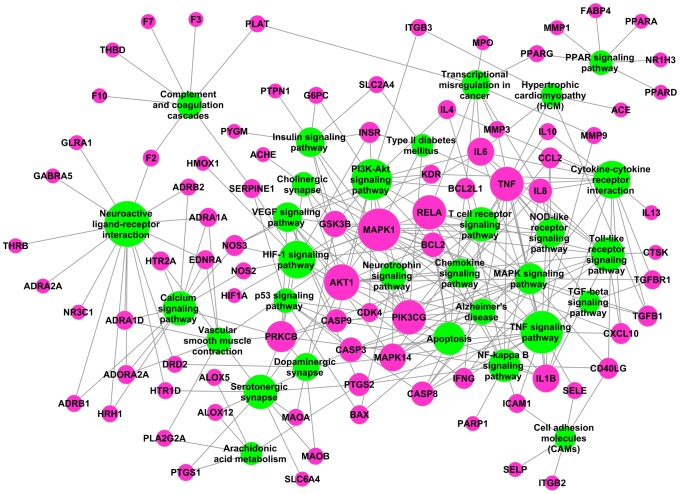
Target-pathway network of anti-stroke herbs where magenta nodes represent the targets and limegreen nodes signify pathways. Node size is proportional to its degree.

Subsequently, all target proteins from target fishing and validation attempt to be mapped onto these 30 pathways, generating a bipartite target-pathway network graph as displayed in Figure 7. Results show that after discarding 9 target proteins without participating in these pathways, this T-P network consists of 119 nodes (85 targets and 30 pathways) and 280 edges. And these pathways are linked with target proteins intensively, such as the TNF signaling pathway which exhibits the largest number of interactions with targets (degree = 17), neuroactive ligand-receptor interaction (degree = 16), PI3K-Akt signaling pathway (degree = 16) and HIF-1 signaling pathway (degree = 14). Actually, plenty of compounds in medicinal herbs are involved in these pathways, which maybe provide basis for stroke treatment and prevention strategies as well. For example, 62 herbal ingredients like ephedrine, corynantheine, ginkgolide A and pseudoephedrine are involved in mediating the major components of neuroactive ligand-receptor interaction pathway including ADRA2A, DRD2 and GABRA2, and might provide neuroprotective strategies to cerebral ischemia reperfusion injury. Analogously, the activation of PI3K-Akt signaling pathway has been proved essential for preventing the neuronal apoptosis and protecting the brain from cerebral ischemia/reperfusion injury [75]. And herbal ingredients like salvianolic acid B [76], cryptotanshinone [50] and ferulic acid [77] have been documented to regulate the PI3K-Akt signaling pathway in cerebral ischemia, thereby exhibiting protective effects against brain injury. Additionally, it is worth noting that several other pathways such as PPAR signaling pathway, arachidonic acid metabolism, insulin signaling pathway and TGF-beta signaling pathway can also be observed in this T-P network, and these pathways are closely related with glucose homeostasis, lipid profile, platelet coagulation and blood pressure. Therefore, we speculate that herbal medicines probably mediate these pathways to exhibit the anti-diabetes, antihypertensive, anti-platelet and anti-hyperlipidemia properties, and thereby might provide a combining system for stroke prevention.

Consequently, we deduce that herbal ingredients mainly target on proteins such as PTGS2, NOS, GSK3B, F2 and CASP3, and thereby have the potential to regulate the pathways like TNF signaling pathway, neuroactive ligand-receptor interaction, PI3K-Akt signaling pathway and HIF-1 signaling pathway to show the anti-apoptosis, anti-oxidant, anti-inflammatory, as well as other neuroprotective effects. Besides these, constituents of these ant-stroke herbs might also disturb PPAR signaling pathway, arachidonic acid metabolism, insulin signaling pathway and other pathways via regulating PPARG, MAPK1, PTGS1 and so forth, and then display anti-diabetes, antihypertensive, anti-platelet and anti-hyperlipidemia properties against recurrent stroke.

## Conclusion

The lack of widely applicable and effective pharmacological therapies for stroke patients results in a growing interest in traditional herbal medications, for which extensive experience and plentiful clinical data on herbal medications in stroke have been accumulated over the past thousands of years. However, unlike conventional pharmacological medications used in western medicine, bioactive compounds and mechanisms of action of herbal medications have not been specified and measured precisely. Therefore, in this paper, we introduced systems pharmacology-based approach, which combines the use of computational modeling and wide-scale text-mining methods, to elucidate the mechanisms of action of the most widely studied medicinal herbs for the stroke treatment and prevention. The main findings are summarized as follows:

1. Based on large-scale text mining method, 10 medicinal herbs such as *S. miltiorrhiza*, *G. biloba*, *E. herba*, etc. have been identified exhibiting significant correlations with stroke, and all these herbs or their corresponding herbal constituents are widely involved in TCPM for stroke treatment in China.
2. After ADME prescreening, 168 compounds with satisfactory pharmacokinetic profiles have the potential to participate in stroke therapy, and the systematic use of these compounds might offer valuable clues on the combination therapies for stroke treatment and prevention.
3. In target fishing, the results display that these 10 anti-stroke herbal medicines probably acts on 94 target proteins, and then exhibit the potential therapeutic benefits in stroke treatment and prevention through the following ways: producing anti-inflammation, anti-oxidant and anti-apoptosis effects against ischemic brain damage, and exhibiting lipid-lowering, anti-diabetic, anti-thrombotic and antiplatelet effects to reduce the risk factors of stroke.
4. The results of C-T network indicate that medicinal herbs exhibit the therapeutic or prophylactic effects against stroke probably through synergistic and additive actions on multiple molecular components involved in the pathogenesis of stroke.
5. The T-P network of anti-stroke herbs constructed in our work demonstrates that herbal medicines might simultaneously target several pathways like PI3K-Akt, TNF and calcium signaling pathways, and thereby exhibit synergistic benefits in stroke treatment and prevention.

## Supporting Information

Supporting Information S1 Table S1, All compounds in ten anti-stroke herbs and the corresponding pharmacokinetics parameters. Table S2, Active constituents of anti-stroke herbs and their corresponding ADME parameters. Table S3, The detailed information of docking validation.
(DOCX)
