# Supplementary material for: Systems Pharmacology Dissection of Multi-Scale Mechanisms of Action for Herbal Medicines in Stroke Treatment and Prevention
Source: PLoS One. 2014 Aug 5;9(8):e102506. doi: 10.1371/journal.pone.0102506 (PMC4122378; doi:10.1371/journal.pone.0102506)
Supplement: Supporting Information S1 — Table S1, All compounds in ten anti-stroke herbs and the corresponding pharmacokinetics parameters. Table S2, Active constituents of anti-stroke herbs and their corresponding ADME parameters. Table S3, The detailed information of docking validation. (DOCX) [file pone.0102506.s001.docx]

Table S1 All compounds in ten anti-stroke herbs and the corresponding pharmacokinetics parameters.

| Number | Compound | OB | Caco-2 | DL | BBB | Log*P* | Log*S* | Herbs |
| --- | --- | --- | --- | --- | --- | --- | --- | --- |
| Molecule0001 | 2-hydroxymethyl-3,5,6-trimethylpyrazine | 11.76 | 0.51 | 0.03 | 0.30 | 0.67 | -1.22 | *R. chuanxiong* |
| Molecule0002 | (+)-alpha-funebrene | 52.87 | 1.80 | 0.10 | 2.09 | 5.18 | -5.11 | *R. chuanxiong* |
| Molecule0003 | (-)-alpha-cubebene | 32.49 | 1.82 | 0.11 | 2.11 | 3.88 | -5.05 | *R. chuanxiong* |
| Molecule0004 | 1,1-dimethylcyclopentane | 41.22 | 1.78 | 0.01 | 2.27 | 3.58 | -3.63 | *R. chuanxiong* |
| Molecule0005 | Senkyunolide-I | 21.38 | 1.00 | 0.08 | 0.90 | 3.69 | -2.62 | *R. chuanxiong* |
| Molecule0006 | Senkyunolide-K | 61.80 | 0.52 | 0.08 | 0.30 | 2.13 | -1.84 | *R. chuanxiong* |
| Molecule0007 | Senkyunolide-M | 3.50 | 0.08 | 0.16 | -0.23 | 2.73 | -2.86 | *R. chuanxiong* |
| Molecule0008 | Senkyunolide-N | 16.62 | -0.15 | 0.10 | -0.45 | 0.94 | -1.03 | *R. chuanxiong* |
| Molecule0009 | Senkyunolide-O | 11.23 | 0.94 | 0.82 | 0.45 | 5.57 | -4.92 | *R. chuanxiong* |
| Molecule0010 | Senkyunolide-P | 9.46 | 0.92 | 0.81 | 0.55 | 5.55 | -4.93 | *R. chuanxiong* |
| Molecule0011 | Senkyunolide-Q | 23.86 | 0.42 | 0.16 | 0.23 | 2.73 | -2.86 | *R. chuanxiong* |
| Molecule0012 | Senkyunolide-R | 14.18 | -0.76 | 0.11 | -1.42 | 0.01 | -1.16 | *R. chuanxiong* |
| Molecule0013 | Trans-ocimene | 23.56 | 1.85 | 0.02 | 2.04 | 4.30 | -2.91 | *R. chuanxiong, E. herba* |
| Molecule0014 | 1,1-diethoxybutane | 29.28 | 1.30 | 0.01 | 1.52 | 2.08 | -1.72 | *R. chuanxiong* |
| Molecule0015 | Valerophenone | 20.76 | 1.46 | 0.03 | 1.53 | 2.94 | -2.97 | *R. chuanxiong* |
| Molecule0016 | Verbenene | 37.71 | 1.80 | 0.05 | 2.16 | 3.64 | -3.19 | *R. chuanxiong* |
| Molecule0017 | Z-6,8',7,3'-diligustilide | 11.95 | 0.74 | 0.70 | 0.31 | 5.54 | -4.89 | *R. chuanxiong* |
| Molecule0018 | Z-Neo-allo-ocimene | 14.89 | 1.86 | 0.02 | 1.92 | 4.36 | -2.45 | *R. chuanxiong* |
| Molecule0019 | Z-butylidenephthalide | 41.80 | 1.32 | 0.07 | 1.27 | 3.38 | -3.11 | *R. chuanxiong* |
| Molecule0020 | Alpha-selinene | 31.81 | 1.82 | 0.10 | 2.14 | 6.58 | -5.52 | *R. chuanxiong* |
| Molecule0021 | 1,2,3,4,4a,7-Hexahydro-l,6-dimethyl-4-(1-methylethyl)-naphthalene | 19.05 | 1.88 | 0.05 | 2.14 | 4.98 | -3.16 | *R. chuanxiong* |
| Molecule0022 | Augustic-acid | 22.71 | 0.18 | 0.74 | -0.38 | 6.06 | -5.07 | *R. chuanxiong* |
| Molecule0023 | Benzene,1-ethyl-3-methyl- | 50.77 | 1.87 | 0.02 | 2.00 | 3.79 | -3.32 | *R. chuanxiong* |
| Molecule0024 | Beta-acoradiene | 40.01 | 1.85 | 0.07 | 2.10 | 5.57 | -4.93 | *R. chuanxiong* |
| Molecule0025 | Beta-pinene | 44.84 | 1.81 | 0.05 | 2.13 | 3.94 | -3.33 | *R. chuanxiong* |
| Molecule0026 | Beta-scliene | 22.19 | 1.83 | 0.08 | 2.11 | 5.26 | -4.79 | *R. chuanxiong* |
| Molecule0027 | 1,3,6-heptatriene,2,5,5-trimethyl- | 42.10 | 1.84 | 0.02 | 2.07 | 4.22 | -3.48 | *R. chuanxiong* |
| Molecule0028 | Beta-cubebene | 31.61 | 1.83 | 0.11 | 2.14 | 3.49 | -4.92 | *R. chuanxiong* |
| Molecule0029 | Butylidene-phthalide | 52.47 | 1.30 | 0.07 | 1.25 | 3.38 | -3.11 | *R. chuanxiong* |
| Molecule0030 | Chrysophanol | 18.55 | 0.60 | 0.21 | -0.20 | 3.08 | -3.31 | *R. chuanxiong* |
| Molecule0031 | Chuanxingol | 30.93 | 0.94 | 0.10 | 0.57 | 3.09 | -2.71 | *R. chuanxiong* |
| Molecule0032 | Cis-asarone | 45.51 | 1.45 | 0.06 | 1.24 | 3.35 | -2.78 | *R. chuanxiong* |
| Molecule0033 | Cnidilide | 77.55 | 1.21 | 0.07 | 1.41 | 3.37 | -3.47 | *R. chuanxiong* |

Table S1: Continued

| Number | Compound | OB | Caco-2 | DL | BBB | Log*P* | Log*S* | Herbs |
| --- | --- | --- | --- | --- | --- | --- | --- | --- |
| Molecule0034 | 1,3,8-p-menthatriene | 35.86 | 1.86 | 0.02 | 2.07 | 4.15 | -2.23 | *R. chuanxiong* |
| Molecule0035 | Cyclodecene | 47.89 | 1.84 | 0.03 | 2.18 | 6.03 | -5.59 | *R. chuanxiong* |
| Molecule0036 | Cyclohexane,1,1,2,3-tetramethyl- | 48.08 | 1.78 | 0.03 | 2.18 | 4.41 | -4.82 | *R. chuanxiong* |
| Molecule0037 | Decane,2,9-dimethyl- | 9.93 | 1.81 | 0.02 | 2.06 | 6.65 | -5.87 | *R. chuanxiong* |
| Molecule0038 | Elemol | 19.51 | 1.34 | 0.07 | 1.34 | 3.78 | -4.67 | *R. chuanxiong* |
| Molecule0039 | Eudesma-4,11-diene | 22.13 | 1.85 | 0.08 | 2.14 | 5.80 | -4.08 | *R. chuanxiong* |
| Molecule0040 | Folic-acid | 70.51 | -1.56 | 0.71 | -2.56 | -0.66 | -3.38 | *R. chuanxiong* |
| Molecule0041 | 1,5,5-Trimethyl-6-methylenecyclohexene | 46.08 | 1.82 | 0.03 | 2.16 | 4.59 | -2.81 | *R. chuanxiong* |
| Molecule0042 | Gamma-bisabolene | 18.95 | 1.91 | 0.06 | 1.92 | 5.88 | -3.55 | *R. chuanxiong* |
| Molecule0043 | Hinesol | 37.83 | 1.44 | 0.09 | 1.62 | 4.84 | -3.79 | *R. chuanxiong* |
| Molecule0044 | Isopropyl-phenyl-ketone | 80.39 | 1.46 | 0.03 | 1.59 | 2.54 | -2.44 | *R. chuanxiong* |
| Molecule0045 | Myricanone | 57.61 | 0.67 | 0.51 | -0.08 | 3.95 | -4.51 | *R. chuanxiong* |
| Molecule0046 | N-caprylaldehyde | 19.06 | 1.30 | 0.01 | 1.49 | 3.19 | -2.65 | *R. chuanxiong* |
| Molecule0047 | 1-(2-Hydroxy-5-methylphenyl)ethanone | 26.39 | 1.19 | 0.03 | 1.28 | 1.91 | -1.78 | *R. chuanxiong* |
| Molecule0048 | P-menth-1-en-4-ol | 81.41 | 1.38 | 0.03 | 1.68 | 2.81 | -1.79 | *R. chuanxiong* |
| Molecule0049 | P-mentha-1,4(8)-diene | 29.54 | 1.86 | 0.02 | 2.09 | 3.82 | -1.99 | *R. chuanxiong* |
| Molecule0050 | 1-acetyl-2-phenylhydrazine | 24.40 | 1.17 | 0.03 | 1.15 | 0.95 | -1.91 | *R. chuanxiong* |
| Molecule0051 | Perlolyrine | 67.82 | 0.88 | 0.27 | 0.15 | 2.66 | -3.47 | *R. chuanxiong* |
| Molecule0052 | Pregnenolone | 14.08 | 0.69 | 0.43 | 0.24 | 4.06 | -4.37 | *R. chuanxiong* |
| Molecule0053 | Sedanoic-acid | 57.14 | 0.37 | 0.06 | 0.37 | 2.66 | -2.44 | *R. chuanxiong* |
| Molecule0054 | Senkyunolide-C | 39.45 | 0.87 | 0.08 | 0.50 | 3.49 | -2.68 | *R. chuanxiong* |
| Molecule0055 | Senkyunolide-D | 23.79 | 0.12 | 0.10 | -0.07 | 0.99 | -1.95 | *R. chuanxiong* |
| Molecule0056 | Senkyunolide-E | 33.55 | 0.55 | 0.08 | 0.06 | 1.93 | -2.39 | *R. chuanxiong* |
| Molecule0057 | Senkyunolide-F | 40.35 | 0.61 | 0.08 | 0.35 | 2.16 | -2.11 | *R. chuanxiong* |
| Molecule0058 | Senkyunolide-J | 21.11 | 0.02 | 0.10 | -0.11 | 0.94 | -1.03 | *R. chuanxiong* |
| Molecule0059 | Senkyunolide-L | 29.68 | 0.63 | 0.09 | 0.40 | 2.64 | -2.44 | *R. chuanxiong* |
| Molecule0060 | Senkyunolide-S | 14.64 | -0.46 | 0.11 | -0.70 | -0.08 | -1.29 | *R. chuanxiong* |
| Molecule0061 | 1-acetyl-carboline | 67.12 | 1.18 | 0.13 | 0.81 | 2.53 | -3.36 | *R. chuanxiong* |
| Molecule0062 | Senkyunone | 15.20 | 1.15 | 0.24 | 0.50 | 5.91 | -5.00 | *R. chuanxiong* |
| Molecule0063 | Sinapic-acid | 59.21 | 0.48 | 0.08 | -0.10 | 1.63 | -2.55 | *R. chuanxiong* |
| Molecule0064 | Trans-2-nonen-1-ol | 37.64 | 1.17 | 0.02 | 1.20 | 3.58 | -2.70 | *R. chuanxiong* |
| Molecule0065 | Trans-piperitol | 47.52 | 1.18 | 0.03 | 1.38 | 2.64 | -1.90 | *R. chuanxiong* |
| Molecule0066 | Trimethylamine | 59.98 | 1.79 | 0.00 | 2.17 | -0.14 | 1.04 | *R. chuanxiong* |
| Molecule0067 | Uracil | 42.53 | 0.03 | 0.02 | -0.13 | -1.28 | -0.64 | *R. chuanxiong* |
| Molecule0068 | Wallichilide | 5.73 | 0.82 | 0.71 | 0.73 | 4.80 | -4.84 | *R. chuanxiong* |
| Molecule0069 | Xiongterpene | 23.77 | 0.44 | 0.42 | -0.20 | 7.16 | -6.34 | *R. chuanxiong* |

Table S1: Continued

| Number | Compound | OB | Caco-2 | DL | BBB | Log*P* | Log*S* | Herbs |
| --- | --- | --- | --- | --- | --- | --- | --- | --- |
| Molecule0070 | (-)-isoledene | 53.28 | 1.83 | 0.10 | 2.08 | 4.98 | -4.18 | *R. chuanxiong* |
| Molecule0071 | 1-octanol,2,7-dimethyl- | 24.50 | 1.25 | 0.02 | 1.30 | 4.01 | -3.22 | *R. chuanxiong* |
| Molecule0072 | 1-beta-ethylacrylate-7-aldehyde-beta-carboline | 29.37 | 0.45 | 0.31 | -0.60 | 2.98 | -4.59 | *R. chuanxiong* |
| Molecule0073 | 1-propanone,1-[2-furanyl]- | 63.49 | 1.21 | 0.02 | 1.37 | 1.39 | -1.02 | *R. chuanxiong* |
| Molecule0074 | 1-propanone,1-phenyl- | 60.17 | 1.45 | 0.02 | 1.56 | 2.15 | -2.12 | *R. chuanxiong* |
| Molecule0075 | 2,2,3-trimethylcyclopent-3-ene-1-carboxaldehyde | 42.64 | 1.31 | 0.03 | 1.60 | 2.75 | -1.71 | *R. chuanxiong* |
| Molecule0076 | 2,2,3-trimethyl-3-cyclopentene-1-acetaldehyde | 45.18 | 1.32 | 0.03 | 1.67 | 3.17 | -2.12 | *R. chuanxiong* |
| Molecule0077 | 2-(1-oxopentyl)-benzoic-acid-methyl-ester | 88.69 | 0.91 | 0.07 | 0.91 | 2.64 | -3.76 | *R. chuanxiong* |
| Molecule0078 | 2-methylhexane | 59.94 | 1.81 | 0.01 | 2.24 | 4.17 | -3.94 | *R. chuanxiong* |
| Molecule0079 | (-)-p-mentha-1,5-diene | 27.74 | 1.86 | 0.02 | 2.09 | 4.29 | -3.01 | *R. chuanxiong* |
| Molecule0080 | 2-furancarboxylic-acid,methyl-ester | 49.41 | 1.12 | 0.02 | 1.34 | 1.21 | -0.77 | *R. chuanxiong* |
| Molecule0081 | 2-methyl-1-propenyl-benzene | 35.00 | 1.88 | 0.02 | 1.95 | 3.78 | -2.79 | *R. chuanxiong* |
| Molecule0082 | 2-methyl-5-(1-methylene)-1,3-cyclohexadiene | 39.91 | 1.83 | 0.03 | 2.12 | 2.69 | -1.29 | *R. chuanxiong* |
| Molecule0083 | 2-methyl-5-(1-methylethenyl)-2-cyclohexen-1-ol | 32.52 | 1.22 | 0.03 | 1.33 | 2.41 | -1.73 | *R. chuanxiong* |
| Molecule0084 | 2-methyl-benzoxazole | 65.25 | 1.31 | 0.03 | 1.33 | 1.90 | -1.49 | *R. chuanxiong* |
| Molecule0085 | 2H-pyran-2-one,tetrahydro-5,6-dimethyl-,trans- | 48.07 | 1.15 | 0.02 | 1.52 | 1.61 | -1.25 | *R. chuanxiong* |
| Molecule0086 | 3(S)-3-butyl-4,5-dihydrophthalide | 25.79 | 1.28 | 0.07 | 1.39 | 3.36 | -2.51 | *R. chuanxiong* |
| Molecule0087 | 3,4-epoxy-2,2,7,7-tetramethyl-octane | 66.87 | 1.56 | 0.05 | 1.84 | 4.06 | -4.50 | *R. chuanxiong* |
| Molecule0088 | 3-carene | 45.15 | 1.85 | 0.04 | 2.16 | 3.64 | -2.96 | *R. chuanxiong* |
| Molecule0089 | 3-cyclohexen-1-ol | 70.57 | 1.14 | 0.01 | 1.52 | 0.96 | -0.55 | *R. chuanxiong* |
| Molecule0090 | 3-furancarboxylic-acid,methyl-ester | 77.82 | 1.03 | 0.02 | 1.11 | 1.09 | -0.76 | *R. chuanxiong* |
| Molecule0091 | 3-methyl-6-(1-methylethyl)-2-cyclohexen-1-ol | 47.83 | 1.19 | 0.03 | 1.38 | 2.64 | -1.90 | *R. chuanxiong* |
| Molecule0092 | 3-methylene-6-(1-methylethyl)-cyclohexene | 40.25 | 1.82 | 0.02 | 2.12 | 3.98 | -3.60 | *R. chuanxiong* |
| Molecule0093 | 4,7-dihydroxy-3-butylphthalide | 105.74 | 0.69 | 0.10 | 0.44 | 2.69 | -2.20 | *R. chuanxiong* |
| Molecule0094 | 4-iodoindoline | 27.36 | 1.79 | 0.03 | 1.89 | 3.17 | -3.08 | *R. chuanxiong* |
| Molecule0095 | 4-octanone | 18.66 | 1.37 | 0.01 | 1.55 | 2.57 | -1.87 | *R. chuanxiong* |

Table S1: Continued

| Number | Compound | OB | Caco-2 | DL | BBB | Log*P* | Log*S* | Herbs |
| --- | --- | --- | --- | --- | --- | --- | --- | --- |
| Molecule0096 | 4-hydroxy-3-butylphthalide | 58.11 | 0.90 | 0.08 | 0.69 | 3.65 | -2.56 | *R. chuanxiong* |
| Molecule0097 | (-)-spathulenol | 25.87 | 1.45 | 0.12 | 1.65 | 2.75 | -3.84 | *R. chuanxiong* |
| Molecule0098 | 5-propyl-2-thiouracil | 77.60 | 0.93 | 0.03 | 0.78 | 1.00 | -2.20 | *R. chuanxiong* |
| Molecule0099 | 6-butyl-1,4-cycloheptadiene | 31.73 | 1.85 | 0.02 | 2.18 | 5.28 | -4.64 | *R. chuanxiong* |
| Molecule0100 | 7-oxabicyclo-2.2.1-heptane,1-methyl-4-[1-methylethyl]- | 60.92 | 1.53 | 0.04 | 1.92 | 2.31 | -3.43 | *R. chuanxiong* |
| Molecule0101 | Alpha-curcumene | 28.47 | 1.93 | 0.06 | 1.96 | 6.15 | -5.31 | *R. chuanxiong* |
| Molecule0102 | Alpha-phellandrene | 27.96 | 1.86 | 0.02 | 2.11 | 4.29 | -3.01 | *R. chuanxiong* |
| Molecule0103 | Aromadendrene-(+) | 55.90 | 1.83 | 0.10 | 2.05 | 3.70 | -4.94 | *R. chuanxiong* |
| Molecule0104 | Aromadendrene-oxide-2 | 64.90 | 1.56 | 0.14 | 1.82 | 2.68 | -5.23 | *R. chuanxiong* |
| Molecule0105 | (1alpha,2beta,4beta)-1-methyl-2,4-bis(methylvinyl)-1-vinylcyclohexane | 24.44 | 1.84 | 0.06 | 2.14 | 5.40 | -4.71 | *R. chuanxiong* |
| Molecule0106 | Alpha-thujene | 48.64 | 1.81 | 0.04 | 2.18 | 4.07 | -2.92 | *R. chuanxiong* |
| Molecule0107 | Amylbenzene | 34.34 | 1.87 | 0.03 | 2.02 | 4.81 | -4.79 | *R. chuanxiong* |
| Molecule0108 | Butanedioic-acid,hydroxy-,dimethyl-ester,(2R)- | 33.95 | 0.11 | 0.03 | -0.06 | -0.59 | 0.29 | *R. chuanxiong* |
| Molecule0109 | Butylphthalide | 49.01 | 1.30 | 0.07 | 1.32 | 3.00 | -3.46 | *R. chuanxiong* |
| Molecule0110 | Carotol | 149.03 | 1.46 | 0.09 | 1.65 | 4.65 | -3.45 | *R. chuanxiong* |
| Molecule0111 | Coniferylfcrulate | 4.54 | 0.67 | 0.39 | -0.53 | 3.47 | -4.78 | *R. chuanxiong* |
| Molecule0112 | D(+)-Sucrose | 1.65 | -2.89 | 0.23 | -6.54 | -2.63 | 0.38 | *R. chuanxiong* |
| Molecule0113 | Limonene | 37.52 | 1.82 | 0.02 | 2.15 | 4.50 | -2.47 | *R. chuanxiong, E. herba* |
| Molecule0114 | Dihydroeugenol | 61.58 | 1.42 | 0.04 | 1.42 | 2.87 | -2.27 | *R. chuanxiong* |
| Molecule0115 | Decahydro-1,6-bis(methylene)-4-(1-methylethyl)-naphthalene | 28.34 | 1.84 | 0.08 | 2.10 | 4.06 | -4.57 | *R. chuanxiong* |
| Molecule0116 | Globulol-(-)- | 81.84 | 1.33 | 0.12 | 1.45 | 3.15 | -4.72 | *R. chuanxiong* |
| Molecule0117 | Hexanophenone | 19.94 | 1.49 | 0.04 | 1.55 | 3.38 | -3.56 | *R. chuanxiong* |
| Molecule0118 | (E,E)-undeca-1,3,5-triene | 34.61 | 1.83 | 0.02 | 1.94 | 5.22 | -4.65 | *R. chuanxiong* |
| Molecule0119 | Hendecanoic-acid | 25.06 | 1.00 | 0.03 | 1.07 | 4.49 | -3.91 | *R. chuanxiong* |
| Molecule0120 | L-valyl-L-valinc-achydride | 37.91 | -0.15 | 0.05 | -0.52 | 0.00 | -1.46 | *R. chuanxiong* |
| Molecule0121 | Levistolide-A | 3.11 | 0.96 | 0.82 | 0.55 | 5.57 | -4.92 | *R. chuanxiong* |
| Molecule0122 | Ligustilide | 51.30 | 1.31 | 0.07 | 1.28 | 3.48 | -2.58 | *R. chuanxiong* |
| Molecule0123 | Methyl-linoleate | 41.93 | 1.49 | 0.17 | 1.21 | 6.95 | -6.76 | *R. chuanxiong* |
| Molecule0124 | (Z,Z)-9,12-Octadecadien-1-ol | 37.76 | 1.41 | 0.12 | 1.02 | 6.99 | -6.62 | *R. chuanxiong* |
| Molecule0125 | Myrcene | 24.96 | 1.84 | 0.02 | 1.98 | 4.32 | -3.25 | *R. chuanxiong* |

Table S1: Continued

| Number | Compound | OB | Caco-2 | DL | BBB | Log*P* | Log*S* | Herbs |
| --- | --- | --- | --- | --- | --- | --- | --- | --- |
| Molecule0126 | N-octatriacontane | 7.91 | 1.98 | 0.37 | 1.21 | 11.26 | -8.03 | *R. chuanxiong* |
| Molecule0127 | Pentyl-benzenemethanol | 55.40 | 1.26 | 0.04 | 1.14 | 3.60 | -3.50 | *R. chuanxiong* |
| Molecule0128 | Sedanolide | 21.24 | 1.25 | 0.07 | 1.38 | 3.28 | -2.82 | *R. chuanxiong* |
| Molecule0129 | Senkyunolide-A | 65.15 | 1.30 | 0.07 | 1.35 | 3.47 | -2.63 | *R. chuanxiong* |
| Molecule0130 | Senkyunolide-G | 39.44 | 0.63 | 0.08 | 0.48 | 2.24 | -2.01 | *R. chuanxiong* |
| Molecule0131 | Senkyunolide-H | 21.97 | 0.00 | 0.10 | -0.23 | 1.20 | -1.55 | *R. chuanxiong* |
| Molecule0132 | (1R, 3R)-dihyd-roxy-(4S, 5R)-dicaffeoyxycyclohe-xane carboxylic acid methyl ester | 1.74 | -0.97 | 0.69 | -2.28 | 2.21 | -3.74 | *E. breviscapu* |
| Molecule0133 | Scopolin | 26.03 | -0.94 | 0.39 | -1.59 | -0.58 | -1.70 | *E. breviscapu* |
| Molecule0134* | Scopolin_DG | 27.50 | 0.74 | 0.08 | 0.34 | 1.62 | -1.77 | *E. breviscapu* |
| Molecule0135 | Apigenin-7-O-β-D-glucuronide-6"-methyl ester | 7.67 | -0.98 | 0.80 | -1.97 | 0.74 | -2.53 | *E. breviscapu* |
| Molecule0136 | Tiliroside | 1.94 | -1.39 | 0.66 | -2.45 | 2.42 | -3.22 | *E. breviscapu* |
| Molecule0137 | Plantaginin | 8.89 | -1.04 | 0.76 | -2.01 | 0.06 | -2.30 | *E. breviscapu* |
| Molecule0138 | Formononetin | 24.40 | 0.84 | 0.21 | 0.25 | 3.01 | -3.51 | *E. breviscapu* |
| Molecule0139 | Isoliquiritigenin | 88.59 | 0.36 | 0.15 | -0.27 | 3.04 | -3.67 | *E. breviscapu* |
| Molecule0140 | 5,7,4'-tirhydroxy flavonoid | 19.98 | 0.36 | 0.21 | -0.44 | 2.47 | -3.11 | *E. breviscapu* |
| Molecule0141 | 1,4-dihyd-roxy-(3R, 5R)-dicaffeoyxycyclohe-xane carboxylic acid methyl ester | 1.75 | -0.93 | 0.68 | -2.10 | 2.22 | -3.72 | *E. breviscapu* |
| Molecule0142 | Gallic acid | 67.96 | -0.10 | 0.04 | -0.43 | 1.17 | -1.54 | *E. breviscapu* |
| Molecule0143 | 4-coumaric acid | 80.70 | 0.45 | 0.04 | 0.14 | 1.74 | -2.21 | *E. breviscapu* |
| Molecule0144 | 1,5-dicatfeoylquinic acid | 1.77 | -1.53 | 0.68 | -2.44 | 2.07 | -3.58 | *E. breviscapu* |
| Molecule0145 | 1,3-dicatfeoyl quinic acid | 1.81 | -1.26 | 0.66 | -2.49 | 2.06 | -3.58 | *E. breviscapu* |
| Molecule0146 | 4,5-dicatfeoyl quinic acid | 1.78 | -1.04 | 0.69 | -2.08 | 2.05 | -3.62 | *E. breviscapu* |
| Molecule0147 | Erigeroside | 13.37 | -1.34 | 0.15 | -1.62 | -2.54 | 0.24 | *E. breviscapu* |
| Molecule0148* | Erigeroside_DG | 18.93 | 0.18 | 0.24 | 0.11 | -1.22 | 0.90 | *E. breviscapu* |
| Molecule0149 | Erigeside D | 20.86 | -0.96 | 0.63 | -1.58 | -0.38 | -2.00 | *E. breviscapu* |
| Molecule0150 | 3,5,6,7,4'-pentahydroxy flavonoid | 27.70 | 0.13 | 0.27 | -0.77 | 1.13 | -2.41 | *E. breviscapu* |
| Molecule0151 | Quercetin-3-O-glucuronide | 4.47 | -1.33 | 0.79 | -1.99 | 0.25 | -2.02 | *E. breviscapu* |
| Molecule0152 | N-butyl-O-β-D-fructopyra noside | 14.80 | -0.58 | 0.08 | -0.81 | -0.85 | 0.09 | *E. breviscapu* |
| Molecule0153* | N-butyl-O-β-D-fructopyra noside_DG | 19.98 | 0.96 | 0.21 | 1.14 | 0.84 | 0.33 | *E. breviscapu* |
| Molecule0154 | 4-O-caffeoyl quinic acid methyl ester | 21.85 | -0.77 | 0.36 | -1.54 | -0.06 | -2.19 | *E. breviscapu* |
| Molecule0155 | Ergosterol | 16.20 | 1.31 | 0.72 | 0.86 | 7.39 | -6.41 | *E. breviscapu* |
| Molecule0156 | Docosanoic acid | 15.69 | 1.26 | 0.26 | 0.95 | 9.19 | -7.05 | *E. breviscapu* |

Table S1: Continued

| Number | Compound | OB | Caco-2 | DL | BBB | Log*P* | Log*S* | Herbs |
| --- | --- | --- | --- | --- | --- | --- | --- | --- |
| Molecule0157 | 6-caffeoylerigeroside | 9.81 | -0.63 | 0.63 | -1.47 | 1.83 | -3.02 | *E. breviscapu* |
| Molecule0158 | 5-O-caffeoyl quinic acid methyl ester | 9.97 | -0.73 | 0.36 | -1.00 | -0.05 | -2.19 | *E. breviscapu* |
| Molecule0159 | Erigerobiside | 6.02 | -2.41 | 0.53 | -2.79 | -2.37 | -0.05 | *E. breviscapu* |
| Molecule0160* | Erigerobiside_DG | 39.88 | 0.23 | 0.02 | 0.17 | -1.22 | 0.90 | *E. breviscapu* |
| Molecule0161 | Plantagin | 16.38 | -1.13 | 0.76 | -2.24 | 0.06 | -2.30 | *E. breviscapu* |
| Molecule0162* | Plantagin_DG | 59.73 | 0.33 | 0.05 | -0.55 | 2.22 | -2.97 | *E. breviscapu* |
| Molecule0163 | 5,6,4'-trihydroxyflavone-7-O-β-D-galactonic acid | 4.63 | -1.37 | 0.79 | -2.16 | 1.21 | -2.22 | *E. breviscapu* |
| Molecule0164* | 5,6,4'-trihydroxyflavone-7-O-β-D-galactonic acid_DG | 38.91 | 0.33 | 0.76 | -0.55 | 2.22 | -2.97 | *E. breviscapu* |
| Molecule0165 | Baicalin hydrate | 15.42 | -0.76 | 0.75 | -1.59 | 0.79 | -2.18 | *E. breviscapu* |
| Molecule0166* | Baicalin hydrate_DG | 16.10 | 0.61 | 0.06 | 0.13 | 2.66 | -2.98 | *E. breviscapu* |
| Molecule0167 | 3,5,6,4’-tetrahydroxy-7-methoxy flavonoid | 55.25 | 0.37 | 0.30 | -0.39 | 1.36 | -2.61 | *E. breviscapu* |
| Molecule0168 | 3,4’-dihydroxybaicalein | 84.75 | -0.57 | 0.04 | -0.51 | -0.33 | -0.23 | *E. breviscapu* |
| Molecule0169 | Naringenin | 39.62 | 0.34 | 0.21 | -0.21 | 2.47 | -3.11 | *E. breviscapu* |
| Molecule0170 | 1-hydroxy-2,3,5-trimethoxyxanthone | 102.76 | 1.06 | 0.30 | 0.30 | 2.96 | -3.49 | *E. breviscapu* |
| Molecule0171 | 5,4’-dihydroxyflavonoid-7-O-β-D-pyranglucuronate buthyl ester | 3.31 | -0.91 | 0.85 | -1.92 | 1.43 | -2.69 | *E. breviscapu* |
| Molecule0172* | 5,4’-dihydroxyflavonoid-7-O-β-D-pyranglucuronate buthyl ester_DG | 47.87 | 0.43 | 0.72 | -0.33 | 2.47 | -3.11 | *E. breviscapu* |
| Molecule0173 | 5,6,4’ -trihydroxyflavonoid-7-O-β-D-pyranolucuronate ethyl ester | 1.05 | -1.07 | 0.83 | -2.00 | 1.21 | -2.35 | *E. breviscapu* |
| Molecule0174 | 3,4-dihydroxybenzoic acid | 24.92 | 0.19 | 0.04 | -0.10 | 1.32 | -1.10 | *E. breviscapu* |
| Molecule0175 | Ethyl caffeate | 6.08 | 0.61 | 0.07 | 0.08 | 2.60 | -2.41 | *E. breviscapu* |
| Molecule0176 | Methyl caffeate | 88.15 | 0.57 | 0.06 | 0.17 | 2.12 | -2.14 | *E. breviscapu* |
| Molecule0177 | (1R,3R)-dihydroxy-(4S,5R)-dicaffeoyloxycyclohexane carboxylic acid methyl ester | 61.82 | -0.60 | 0.69 | -1.76 | 2.21 | -3.73 | *E. breviscapu* |
| Molecule0178 | 1,4-di hydroxy-(3R,5R)-dicaffeoyloxycyclohexane carboxylic acid methyl ester | 44.07 | -0.84 | 0.64 | -2.11 | 2.09 | -3.84 | *E. breviscapu* |
| Molecule0179 | 1-O-methyl-3,5-O-dicaffcoyi quinic acid methyl ester | 1.53 | -0.29 | 0.69 | -1.73 | 2.83 | -4.43 | *E. breviscapu* |

Table S1: Continued

| Number | Compound | OB | Caco-2 | DL | BBB | Log*P* | Log*S* | Herbs |
| --- | --- | --- | --- | --- | --- | --- | --- | --- |
| Molecule0180 | 5-0-methyl-caffeoyl quinic acid butyl ester | 8.65 | -0.70 | 0.46 | -1.55 | 1.00 | -2.52 | *E. breviscapu* |
| Molecule0181 | 1-(2’-γ-pyranone) -6-caffeoyl-α-D-pyranoglucose | 6.16 | -1.01 | 0.62 | -1.64 | -0.02 | -2.23 | *E. breviscapu* |
| Molecule0182 | 3,5-O-dicaffcoyl quinic acid | 1.79 | -1.49 | 0.69 | -2.46 | 2.05 | -3.61 | *E. breviscapu* |
| Molecule0183 | 3,5-O-dicaffeoyl quinic acid methyl ester | 1.73 | -1.30 | 0.68 | -2.39 | 2.05 | -3.76 | *E. breviscapu* |
| Molecule0184 | 4’-hydroxybaicalein | 59.56 | 0.31 | 0.24 | -0.43 | 2.22 | -2.97 | *E. breviscapu* |
| Molecule0185 | 3,4-O-dicaffeoyl quinic acid methyl ester | 1.73 | -1.12 | 0.69 | -2.41 | 2.08 | -3.76 | *E. breviscapu* |
| Molecule0186 | Isoscopolefin | 25.34 | 0.77 | 0.08 | 0.48 | 1.57 | -1.76 | *E. breviscapu* |
| Molecule0187 | (Z)-cinnamic acid | 20.37 | 1.03 | 0.03 | 1.05 | 2.38 | -2.38 | *E. breviscapu* |
| Molecule0188 | β-(p-methoxyphenyl)acrylic acid/4-methoxycinnamic acid | 51.54 | 0.78 | 0.05 | 0.66 | 2.37 | -2.51 | *E. breviscapu* |
| Molecule0189 | 3,5-dimethoxybenzene carbonic acid-4-O-β-D-pyranglucose | 50.24 | -0.89 | 0.23 | -1.49 | -1.21 | -1.34 | *E. breviscapu* |
| Molecule0190* | 3,5-dimethoxybenzene carbonic acid-4-O-β-D-pyranglucose_DG | 23.70 | 0.81 | 0.07 | 0.89 | 1.65 | -1.93 | *E. breviscapu* |
| Molecule0191 | Fridelin | 11.44 | 1.44 | 0.76 | 1.39 | 6.66 | -7.15 | *E. breviscapu* |
| Molecule0192 | Fridelane | 6.70 | 1.81 | 0.75 | 1.99 | 7.32 | -7.34 | *E. breviscapu* |
| Molecule0193 | Fridelinol | 10.96 | 1.30 | 0.76 | 1.11 | 6.38 | -7.02 | *E. breviscapu* |
| Molecule0194 | Pyromeconic acid | 33.92 | 0.18 | 0.02 | 0.11 | -1.22 | 0.90 | *E. breviscapu* |
| Molecule0195 | Epi-fridelinol | 17.60 | 1.43 | 0.76 | 1.25 | 6.38 | -7.02 | *E. breviscapu* |
| Molecule0196 | Pentatetracontanoic acid | 11.05 | 1.43 | 0.21 | 0.37 | 10.94 | -7.81 | *E. breviscapu* |
| Molecule0197 | Ergosta-7,22-dien-3-one | 44.88 | 1.48 | 0.72 | 1.18 | 6.86 | -6.96 | *E. breviscapu* |
| Molecule0198 | α-methoxy-γ-pyranone | 42.13 | 0.67 | 0.02 | 0.60 | -0.44 | 0.41 | *E. breviscapu* |
| Molecule0199 | Stigmasteml-3-O-β-D-glucopyranoside | 21.07 | -0.16 | 0.59 | -0.86 | 5.53 | -5.30 | *E. breviscapu* |
| Molecule0200* | Stigmasteml-3-O-β-D-glucopyranoside_DG | 41.08 | 1.31 | 0.02 | 0.94 | 6.95 | -7.18 | *E. breviscapu* |
| Molecule0201 | Blumenol C glucoside | 19.57 | -0.64 | 0.33 | -1.06 | 0.49 | -2.14 | *E. breviscapu* |
| Molecule0202* | Blumenol C glucoside_DG | 36.34 | 0.64 | 0.05 | 0.39 | 2.39 | -2.56 | *E. breviscapu* |
| Molecule0203 | (+) - syringarcsinol-O-β-D-glueopyranoside | 43.35 | -0.85 | 0.77 | -1.73 | 0.98 | -3.10 | *E. breviscapu* |
| Molecule0204* | (+) - syringarcsinol-O-β-D-glueopyranoside_DG | 9.01 | 0.66 | 0.05 | -0.38 | 2.23 | -3.94 | *E. breviscapu* |

Table S1: Continued

| Number | Compound | OB | Caco-2 | DL | BBB | Log*P* | Log*S* | Herbs |
| --- | --- | --- | --- | --- | --- | --- | --- | --- |
| Molecule0205 | 3,5-dimethoxy-4-hydroxybenzene carbonic acid | 98.90 | 0.60 | 0.06 | 0.61 | 1.48 | -2.00 | *E. breviscapu* |
| Molecule0206 | 6-methoxycaumarin-7-O-β-D-glucopyranoside | 28.69 | -1.11 | 0.43 | -1.70 | -0.55 | -1.70 | *E. breviscapu* |
| Molecule0207* | 6-methoxycaumarin-7-O-β-D-glucopyranoside_DG | 26.40 | 0.73 | 0.13 | 0.38 | 1.62 | -1.77 | *E. breviscapu* |
| Molecule0208 | (E)-ethyl 3-(3,4-dihydroxyphenyl)acrylate | 6.08 | 0.65 | 0.07 | 0.22 | 2.60 | -2.41 | *E. breviscapu* |
| Molecule0209 | 7-(3,5-dimethoxy-4-hydroxybenzcne carbonic acid)-β-D-glucopyranose | 19.46 | -0.92 | 0.30 | -1.51 | -0.86 | -1.53 | *E. breviscapu* |
| Molecule0210* | 7-(3,5-dimethoxy-4-hydroxybenzcne carbonic acid)-β-D-glucopyranose_DG | 25.83 | 0.45 | 0.01 | 0.08 | 1.55 | -1.73 | *E. breviscapu* |
| Molecule0211 | Esculin | 14.38 | -1.17 | 0.36 | -1.92 | -0.93 | -1.47 | *E. breviscapu* |
| Molecule0212* | Esculin_DG | 28.51 | 0.44 | 0.11 | 0.17 | 1.21 | -1.34 | *E. breviscapu* |
| Molecule0213 | Erigoster A | 1.99 | -0.80 | 0.70 | -2.05 | 2.23 | -3.87 | *E. breviscapu* |
| Molecule0214 | Pcnta-acctate of efigoster A | 14.70 | -0.67 | 0.33 | -1.68 | 4.68 | -6.23 | *E. breviscapu* |
| Molecule0215 | Erigeside I | 7.53 | -1.22 | 0.62 | -2.02 | -0.02 | -2.23 | *E. breviscapu* |
| Molecule0216 | Erigeside II | 34.27 | -0.43 | 0.28 | -1.02 | 0.38 | -1.72 | *E. breviscapu* |
| Molecule0217* | Erigeside II_DG | 71.72 | 1.29 | 0.24 | 1.08 | 2.23 | -2.09 | *E. breviscapu* |
| Molecule0218 | Erigeside A | 4.51 | -0.92 | 0.50 | -1.49 | -0.39 | -2.04 | *E. breviscapu* |
| Molecule0219* | Erigeside A_DG | 45.56 | 0.34 | 0.21 | 0.05 | 1.46 | -1.78 | *E. breviscapu* |
| Molecule0220 | Erigeside B | 10.57 | -0.49 | 0.11 | -0.83 | 0.16 | -0.65 | *E. breviscapu* |
| Molecule0221* | Erigeside B_DG | 42.19 | 1.07 | 0.24 | 1.28 | 1.79 | -0.87 | *E. breviscapu* |
| Molecule0222 | Icariside B2 | 17.50 | -0.97 | 0.39 | -1.47 | -0.20 | -1.90 | *E. breviscapu* |
| Molecule0223* | Icariside B2_DG | 21.57 | 0.33 | 0.02 | -0.07 | 1.63 | -2.30 | *E. breviscapu* |
| Molecule0224 | 3,4,5-tricaffeoylquinic acid | 3.01 | -1.51 | 0.46 | -2.81 | 3.48 | -4.71 | *E. breviscapu* |
| Molecule0225 | 27-nor-olean-3-hydroxy-28→13 oate | 17.11 | 0.70 | 0.69 | 0.19 | 5.51 | -6.46 | *U. rhynchophylla* |
| Molecule0226 | Nemerosin | 51.78 | 0.83 | 0.65 | -0.09 | 3.48 | -4.96 | *U. rhynchophylla* |
| Molecule0227 | Ajmalicine | 55.19 | 0.94 | 0.81 | 0.33 | 3.41 | -3.54 | *U. rhynchophylla* |
| Molecule0228 | Angustidine | 41.37 | 1.15 | 0.66 | 0.16 | 2.93 | -3.63 | *U. rhynchophylla* |
| Molecule0229 | L-epicatechin | 53.89 | 0.01 | 0.24 | -0.65 | 1.02 | -2.65 | *U. rhynchophylla, G. biloba* |
| Molecule0230 | Pomolic acid | 16.48 | 0.16 | 0.73 | -0.43 | 5.90 | -5.35 | *U. rhynchophylla* |
| Molecule0231 | Geissoschizinc acid | 42.27 | 0.44 | 0.60 | 0.12 | 3.69 | -3.58 | *U. rhynchophylla* |
| Molecule0232 | Strictosamide | 7.68 | -0.52 | 0.67 | -1.27 | 0.65 | -2.47 | *U. rhynchophylla* |
| Molecule0233* | Strictosamide_DG | 23.88 | 0.50 | 0.76 | -0.34 | 1.80 | -2.89 | *U. rhynchophylla* |

Table S1: Continued

| Number | Compound | OB | Caco-2 | DL | BBB | Log*P* | Log*S* | Herbs |
| --- | --- | --- | --- | --- | --- | --- | --- | --- |
| Molecule0234 | Vallesiachotamine | 53.45 | 1.01 | 0.60 | 0.62 | 3.05 | -3.85 | *U. rhynchophylla* |
| Molecule0235 | Pteropodine | 62.34 | 0.09 | 0.75 | -0.19 | 2.31 | -3.06 | *U. rhynchophylla* |
| Molecule0236 | Glyceryl monopalmitate | 26.66 | 0.15 | 0.22 | -0.63 | 5.73 | -4.85 | *U. rhynchophylla* |
| Molecule0237 | Syringic acid | 36.81 | 0.50 | 0.06 | 0.08 | 1.55 | -1.73 | *U. rhynchophylla, E. breviscapu* |
| Molecule0238 | Scopoletin | 33.19 | 0.66 | 0.08 | 0.23 | 1.62 | -1.77 | *U. rhynchophylla, E. breviscapu* |
| Molecule0239 | Methyl 4-hydroxycinnamate | 35.68 | 0.81 | 0.05 | 0.91 | 2.42 | -2.26 | *U. rhynchophylla* |
| Molecule0240 | Dihydrocorynantheine | 29.49 | 1.14 | 0.67 | 0.75 | 4.70 | -4.68 | *U. rhynchophylla* |
| Molecule0241 | Rhynchophylline A | 37.15 | 0.40 | 0.69 | -0.15 | 2.29 | -3.19 | *U. rhynchophylla* |
| Molecule0242 | Geissoschizinc | 48.77 | 0.70 | 0.64 | 0.37 | 3.84 | -4.12 | *U. rhynchophylla* |
| Molecule0243 | Rhynchophylline C | 62.34 | 0.09 | 0.75 | -0.19 | 2.31 | -3.06 | *U. rhynchophylla* |
| Molecule0244 | Rhynchophylline | 47.86 | 0.70 | 0.57 | 0.38 | 2.85 | -3.66 | *U. rhynchophylla* |
| Molecule0245 | Rhynchophylline E | 79.92 | 0.28 | 0.75 | -0.24 | 2.31 | -3.06 | *U. rhynchophylla* |
| Molecule0246 | Rhynchophylline N-oxide | 51.19 | 0.58 | 0.57 | 0.33 | 2.85 | -3.66 | *U. rhynchophylla* |
| Molecule0247 | Harmane | 38.58 | 1.52 | 0.10 | 1.26 | 3.36 | -3.28 | *U. rhynchophylla* |
| Molecule0248 | Isorhynchophylline N-oxide | 15.37 | 0.41 | 0.59 | -0.08 | 0.62 | -4.60 | *U. rhynchophylla* |
| Molecule0249 | 8-gingerol | 7.01 | 0.55 | 0.22 | -0.09 | 4.33 | -3.87 | *U. rhynchophylla* |
| Molecule0250 | Hyperoside | 6.94 | -1.42 | 0.77 | -2.06 | 0.02 | -1.98 | *U. rhynchophylla* |
| Molecule0251 | Hirsutaside A | 70.34 | 0.34 | 0.81 | -0.10 | 3.23 | -4.85 | *U. rhynchophylla* |
| Molecule0252 | Akuammigine | 65.04 | 0.72 | 0.81 | 0.54 | 3.41 | -3.54 | *U. rhynchophylla* |
| Molecule0253 | Corynoxine | 38.51 | 0.63 | 0.57 | 0.33 | 2.85 | -3.66 | *U. rhynchophylla* |
| Molecule0254 | Corynoxine B | 54.47 | 0.64 | 0.57 | 0.25 | 2.85 | -3.66 | *U. rhynchophylla* |
| Molecule0255 | Mitraphyllic acid | 31.70 | 0.02 | 0.70 | -0.25 | 1.67 | -2.57 | *U. rhynchophylla* |
| Molecule0256 | Mitraphylline | 17.68 | 0.10 | 0.75 | -0.17 | 2.31 | -3.06 | *U. rhynchophylla* |
| Molecule0257 | Hirsutaside B | 40.21 | 0.74 | 0.80 | 0.08 | 5.27 | -5.76 | *U. rhynchophylla* |
| Molecule0258 | Corynoxeine | 18.05 | 0.85 | 0.57 | 0.50 | 2.61 | -3.47 | *U. rhynchophylla* |
| Molecule0259 | Hirsuteine | 56.27 | 0.92 | 0.64 | 0.61 | 3.33 | -4.04 | *U. rhynchophylla* |
| Molecule0260 | Kaempferol-3-O-glalctoside | 2.53 | -1.36 | 0.74 | -2.02 | -0.08 | -2.03 | *U. rhynchophylla* |
| Molecule0261 | Rhynchophine | 13.52 | -0.75 | 0.20 | -2.11 | 2.67 | -3.80 | *U. rhynchophylla* |
| Molecule0262 | Tetrahydroalstonine | 41.30 | 1.13 | 0.81 | 0.69 | 4.03 | -3.71 | *U. rhynchophylla* |
| Molecule0263 | (2S,12bR)-methyl 2-((E)-1-oxobut-2-en-2-yl)-1,2,6,7,12,12b-hexahydroindolo[2,3-a]quinolizine-3-carboxylate | 42.82 | 1.08 | 0.60 | 0.63 | 3.05 | -3.85 | *U. rhynchophylla* |
| Molecule0264 | Vincoside lactam | 14.02 | -0.85 | 0.56 | -1.61 | 0.85 | -2.02 | *U. rhynchophylla* |

Table S1: Continued

| Number | Compound | OB | Caco-2 | DL | BBB | Log*P* | Log*S* | Herbs |
| --- | --- | --- | --- | --- | --- | --- | --- | --- |
| Molecule0265* | Vincoside lactam_DG | 27.14 | 0.47 | 0.82 | -0.16 | 2.42 | -2.52 | *U. rhynchophylla* |
| Molecule0266 | Hirsutaside C | 34.27 | 0.13 | 0.75 | -1.02 | 5.06 | -6.45 | *U. rhynchophylla* |
| Molecule0267 | β-yohimbine | 49.15 | 0.44 | 0.81 | -0.10 | 2.36 | -3.01 | *U. rhynchophylla* |
| Molecule0268 | Isoformosanine | 122.86 | 0.34 | 0.75 | 0.06 | 2.31 | -3.06 | *U. rhynchophylla* |
| Molecule0269 | Isopteropodine | 69.54 | 0.38 | 0.75 | 0.09 | 2.31 | -3.06 | *U. rhynchophylla* |
| Molecule0270 | Isorhynchophylline | 49.31 | 0.29 | 0.52 | -0.21 | 2.47 | -3.36 | *U. rhynchophylla* |
| Molecule0271 | Isoquercitrin | 35.78 | -1.47 | 0.77 | -2.13 | 0.02 | -1.98 | *U. rhynchophylla* |
| Molecule0272 | Geissoschizine methyl ether | 32.70 | 0.97 | 0.64 | 0.66 | 3.84 | -4.12 | *U. rhynchophylla* |
| Molecule0273 | Oleamide | 31.20 | 1.26 | 0.14 | 1.11 | 7.19 | -6.59 | *U. rhynchophylla* |
| Molecule0274 | Quercetin 3-β-D-glucoside | 8.44 | -1.47 | 0.77 | -2.13 | 0.02 | -1.98 | *U. rhynchophylla* |
| Molecule0275 | Corynantheine | 56.27 | 0.93 | 0.64 | 0.51 | 3.33 | -4.04 | *U. rhynchophylla* |
| Molecule0276 | Angustoline | 33.15 | 0.41 | 0.77 | -0.31 | 1.84 | -3.28 | *U.rhynchophylla* |
| Molecule0277 | Cadambine | 17.81 | -1.02 | 0.34 | -1.53 | 0.15 | -2.20 | *U. rhynchophylla* |
| Molecule0278* | Cadambine_DG | 99.89 | 0.15 | 0.84 | -0.34 | 1.81 | -2.39 | *U. rhynchophylla* |
| Molecule0279 | Isorhychophylline | 5.02 | 0.50 | 0.57 | 0.24 | 2.85 | -3.66 | *U. rhynchophylla* |
| Molecule0280 | Uncarine F | 68.17 | 0.49 | 0.75 | 0.17 | 2.31 | -3.06 | *U. rhynchophylla* |
| Molecule0281 | Speciophylline | 15.08 | 0.12 | 0.75 | 0.00 | 2.31 | -3.06 | *U. rhynchophylla* |
| Molecule0282 | Angustine | 32.91 | 1.14 | 0.71 | 0.46 | 2.79 | -3.97 | *U. rhynchophylla* |
| Molecule0283 | Isorhynchophyllic acid | 46.90 | 0.29 | 0.52 | -0.21 | 2.47 | -3.36 | *U. rhynchophylla* |
| Molecule0284 | 3α-dihydrocadambine | 8.95 | -0.93 | 0.52 | -1.51 | -0.15 | -2.14 | *U. rhynchophylla* |
| Molecule0285* | 3α-dihydrocadambine_DG | 65.70 | 0.12 | 0.88 | -0.27 | 1.36 | -2.20 | *U. rhynchophylla* |
| Molecule0286 | Isocorynantheic acid | 50.32 | 0.77 | 0.60 | 0.26 | 3.32 | -3.63 | *U. rhynchophylla* |
| Molecule0287 | 3β-isodihydrocadambine | 11.65 | -1.14 | 0.54 | -2.14 | -0.13 | -2.28 | *U. rhynchophylla* |
| Molecule0288* | 3β-isodihydrocadambine_DG | 28.98 | 0.04 | 0.87 | -0.72 | 1.34 | -2.27 | *U. rhynchophylla* |
| Molecule0289 | 3β,6β-23-trihydroxy-urs-12-en-28-oic acid | 29.53 | -0.19 | 0.72 | -0.82 | 4.54 | -4.59 | *U. rhynchophylla* |
| Molecule0290 | Uncarinic acid A | 17.77 | -0.01 | 0.45 | -0.86 | 6.57 | -5.76 | *U. rhynchophylla* |
| Molecule0291 | Uncarinic acid B | 22.30 | 0.12 | 0.39 | -0.68 | 6.81 | -6.08 | *U. rhynchophylla* |
| Molecule0292 | Uncarinic acid C | 22.66 | 0.01 | 0.39 | -1.15 | 7.30 | -6.09 | *U. rhynchophylla* |
| Molecule0293 | Uncarinic acid D | 22.66 | 0.07 | 0.39 | -0.81 | 7.30 | -6.09 | *U. rhynchophylla* |
| Molecule0294 | Uncarinic acid E | 22.94 | 0.20 | 0.45 | -0.79 | 6.88 | -6.04 | *U. rhynchophylla* |
| Molecule0295 | Hyperin | 21.84 | -1.22 | 0.77 | -2.09 | 0.02 | -1.98 | *U. rhynchophylla* |
| Molecule0296 | Arzelin | 2.98 | -0.71 | 0.70 | -1.58 | 0.77 | -2.17 | *U. rhynchophylla* |
| Molecule0297* | Arzelin_DG | 71.18 | 0.25 | 0.24 | -0.47 | 1.23 | -2.47 | *U. rhynchophylla* |
| Molecule0298 | NcariasideA | 6.03 | -1.84 | 0.14 | -2.74 | 1.54 | -3.53 | *U. rhynchophylla* |

Table S1: Continued

| Number | Compound | OB | Caco-2 | DL | BBB | Log*P* | Log*S* | Herbs |
| --- | --- | --- | --- | --- | --- | --- | --- | --- |
| Molecule0299* | NcariasideA_DG | 19.39 | 0.29 | 0.78 | -0.25 | 4.68 | -4.91 | *U. rhynchophylla* |
| Molecule0300 | Manghaslin | 3.22 | -2.85 | 0.41 | -3.60 | -0.05 | -1.67 | *U. rhynchophylla* |
| Molecule0301* | Manghaslin_DG | 8.22 | 0.06 | 0.28 | -0.64 | 1.07 | -2.42 | *U. rhynchophylla* |
| Molecule0302 | Isoscutellarein | 42.79 | 0.83 | 0.02 | 0.03 | 2.68 | -3.25 | *S. baicalensis* |
| Molecule0303 | ( tans )-5,7,2',6'-Tetrahydroxyflavanonol | 59.25 | -0.33 | 0.27 | -0.84 | 1.01 | -2.21 | *S. baicalensis* |
| Molecule0304 | 2,6,2',4'-Tetrahydroxy-6'-methoxychalcone | 83.46 | 0.22 | 0.22 | -0.43 | 2.71 | -3.62 | *S. baicalensis* |
| Molecule0305 | Leucosceptoside A | 4.17 | -1.78 | 0.61 | -2.90 | 1.23 | -2.92 | *S. baicalensis* |
| Molecule0306 | Martynoside | 12.91 | -1.59 | 0.58 | -2.51 | 1.07 | -3.10 | *S. baicalensis* |
| Molecule0307 | 4-hydroxy-β-phenylethyl-β-d-glucopyranoside | 7.01 | -0.74 | 0.20 | -1.31 | -1.16 | -1.36 | *S. baicalensis* |
| Molecule0308* | 4-hydroxy-β-phenylethyl-β-D-glucopyranoside_DG | 24.79 | 0.63 | 0.02 | 0.33 | 0.85 | -0.74 | *S. baicalensis* |
| Molecule0309 | Catalpol | 2.83 | -1.89 | 0.44 | -2.70 | -2.19 | -0.19 | *S. baicalensis* |
| Molecule0310* | Catalpol_DG | 27.26 | -0.64 | 0.10 | -1.36 | -1.49 | 0.32 | *S. baicalensis* |
| Molecule0311 | (+)-pulegone | 51.25 | 1.38 | 0.03 | 1.64 | 2.36 | -1.82 | *S. baicalensis* |
| Molecule0312* | Dihydrobaicalin_DG | 40.24 | 0.55 | 0.75 | 0.18 | 2.66 | -2.98 | *S. baicalensis* |
| Molecule0313* | Darendoside A_DG | 65.04 | 0.61 | 0.67 | 0.21 | 0.74 | -1.12 | *S. baicalensis* |
| Molecule0314* | Darendoside B_DG | 34.44 | -0.76 | 0.59 | -1.11 | -0.16 | -1.28 | *S. baicalensis* |
| Molecule0315* | Salidroside_DG | 26.77 | 0.65 | 0.20 | 0.25 | 0.85 | -0.74 | *S. baicalensis* |
| Molecule0316 | Scutellarin | 45.56 | 0.31 | 0.21 | -0.36 | 2.22 | -2.97 | *E. breviscapu, S. baicalensis* |
| Molecule0317 | 2',5,8-trihydroxy-6,7-dimethoxyflavone | 63.69 | 0.47 | 0.33 | -0.12 | 3.01 | -3.34 | *S. baicalensis* |
| Molecule0318 | 5,2',6'-trihydroxy-7,8-dimethoxyflavone | 48.71 | 0.45 | 0.33 | -0.10 | 2.47 | -3.29 | *S. baicalensis* |
| Molecule0319 | 5,7,2'-trihydroxy-8,6'-dimethoxyflavone | 43.84 | 0.44 | 0.34 | -0.15 | 2.48 | -3.32 | *S. baicalensis* |
| Molecule0320 | 5,2',5'-trihydroxy-6,7,8-trimethoxyflavone | 34.47 | 0.34 | 0.40 | -0.48 | 2.29 | -3.36 | *S. baicalensis* |
| Molecule0321 | (2S)-7,2',6'-trihydroxy-5-methoxyflavanone | 63.69 | 0.26 | 0.27 | -0.26 | 2.43 | -3.19 | *S. baicalensis* |
| Molecule0322 | 5,7,4'-trihydroxy-6-methoxyflavanone | 55.41 | 0.34 | 0.27 | -0.32 | 2.56 | -3.31 | *S. baicalensis* |
| Molecule0323 | 5,8,2'-trihydroxy-7-methoxyflavone | 61.85 | 0.60 | 0.27 | -0.01 | 2.51 | -3.12 | *S. baicalensis* |
| Molecule0324 | 5,7,2'-trihydroxy-8-methoxyflavone | 44.85 | 0.57 | 0.27 | 0.08 | 2.51 | -3.15 | *S. baicalensis* |
| Molecule0325 | (2R,3R)-3,5,7-trihydroxyflavanone | 27.01 | 0.20 | 0.21 | -0.24 | 1.46 | -2.45 | *S. baicalensis* |
| Molecule0326 | 5,7,2'-trihydroxyflavone | 22.24 | 0.56 | 0.21 | -0.05 | 2.45 | -2.95 | *S. baicalensis* |
| Molecule0327 | Oroxylin-A-7-O-glucuronide | 26.01 | -0.78 | 0.78 | -1.50 | 0.44 | -2.33 | *S. baicalensis* |

Table S1: Continued

| Number | Compound | OB | Caco-2 | DL | BBB | Log*P* | Log*S* | Herbs |
| --- | --- | --- | --- | --- | --- | --- | --- | --- |
| Molecule0328 | 3,5,7,2',6'-pentahydroxyflavanone | 68.45 | -0.27 | 0.27 | -0.80 | 1.01 | -2.21 | *S. baicalensis* |
| Molecule0329 | Norwogonin | 20.53 | 0.62 | 0.21 | 0.04 | 2.59 | -2.92 | *S. baicalensis* |
| Molecule0330 | Rehderianin I | 36.31 | 0.44 | 0.33 | -0.25 | 2.50 | -3.37 | *S. baicalensis* |
| Molecule0331 | Ganhuangenin | 47.07 | 0.26 | 0.37 | -0.39 | 2.19 | -3.13 | *S. baicalensis* |
| Molecule0332 | 7-methoxybaicalein | 32.65 | 0.78 | 0.23 | 0.34 | 3.09 | -3.37 | *S. baicalensis* |
| Molecule0333 | (2R,3R)-2',3,5,7-tetrahydroxyflavanone | 33.88 | -0.29 | 0.24 | -0.70 | 1.18 | -2.30 | *S. baicalensis* |
| Molecule0334 | (2S)-2',5,6',7-tetrahydroxyflavanone | 63.59 | 0.16 | 0.24 | -0.29 | 2.04 | -2.84 | *S. baicalensis* |
| Molecule0335 | 5,7,2',3'-tetrahydroxyflavone | 1.32 | 0.23 | 0.24 | -0.29 | 2.09 | -2.88 | *S. baicalensis* |
| Molecule0336 | 5,7,2',5'-tetrahydroxyflavone | 19.02 | 0.26 | 0.24 | -0.59 | 2.04 | -2.89 | *S. baicalensis* |
| Molecule0337 | Rivularin (flavone) | 50.09 | 0.67 | 0.37 | 0.12 | 2.10 | -3.49 | *S. baicalensis* |
| Molecule0338 | Wogonoside | 8.07 | -1.16 | 0.63 | -1.78 | 0.44 | -2.45 | *S. baicalensis* |
| Molecule0339 | Wogonin-5-β-D-glucoside | 20.13 | -0.52 | 0.75 | -1.47 | 0.34 | -2.45 | *S. baicalensis* |
| Molecule0340 | Moslosooflavone/5-Hydroxy-7,8-dimethoxyflavone | 40.78 | 1.00 | 0.25 | 0.57 | 3.00 | -3.70 | *S. baicalensis* |
| Molecule0341 | Skullcapflavone II | 61.22 | 0.63 | 0.44 | 0.00 | 1.76 | -3.45 | *S. baicalensis* |
| Molecule0342 | Scuteamoenoside | 8.03 | -1.30 | 0.84 | -1.96 | 0.10 | -2.46 | *S. baicalensis* |
| Molecule0343* | Scuteamoenoside_DG | 69.58 | 0.34 | 0.27 | -0.08 | 2.45 | -3.16 | *S. baicalensis* |
| Molecule0344 | Scuteamoenin | 3.04 | -3.08 | 0.16 | -4.41 | -1.61 | -4.95 | *S. baicalensis* |
| Molecule0345 | 6-hydroxyflavone | 23.59 | 0.91 | 0.16 | 0.40 | 3.31 | -3.34 | *S. baicalensis* |
| Molecule0346 | Tenaxin-II | 29.43 | 0.35 | 0.27 | -0.40 | 2.53 | -3.13 | *S. baicalensis* |
| Molecule0347 | 5,7,2'-trihydroxy-6-methoxyflavone | 34.97 | 0.49 | 0.27 | 0.04 | 2.53 | -3.13 | *S. baicalensis* |
| Molecule0348 | 5,2',6'-trihydroxy-6,7,8-trimethoxyflavone-2'-O-D-glucoside | 5.66 | -1.20 | 0.85 | -1.98 | 1.11 | -2.51 | *S. baicalensis* |
| Molecule0349* | 5,2',6'-trihydroxy-6,7,8-trimethoxyflavone-2'-O-D-glucoside_DG | 46.10 | 0.38 | 0.40 | -0.23 | 2.22 | -3.28 | *S. baicalensis* |
| Molecule0350 | 5,2',6'-trihydroxy-6,7-dimethoxyflavone-2'-O- D-glucoside | 4.08 | -1.28 | 0.86 | -2.15 | 0.87 | -2.40 | *S. baicalensis* |
| Molecule0351* | 5,2',6'-Trihydroxy-6,7-dimethoxyflavone-2'-O- D-glucoside_DG | 77.59 | 0.40 | 0.33 | -0.23 | 2.49 | -3.31 | *S. baicalensis* |
| Molecule0352 | Savligenin | 48.73 | 0.85 | 0.33 | -0.08 | 1.95 | -3.87 | *S. baicalensis* |
| Molecule0353 | 5,7-dihydroxy-6,8,2',3'-tetramethoxyflavone | 69.65 | 0.70 | 0.44 | 0.10 | 1.80 | -3.58 | *S. baicalensis* |
| Molecule0354 | 6,2'-dihydroxy-5,7,8,6'-tetramethoxyflavone | 38.58 | 0.58 | 0.44 | 0.11 | 1.75 | -3.49 | *S. baicalensis* |
| Molecule0355 | Isoscutellarein-8-O-D-glucuronide | 31.81 | -1.08 | 0.72 | -1.76 | 0.15 | -2.72 | *S. baicalensis* |
| Molecule0356 | 8-methoxy-5-O-glucoside flavone | 10.96 | -0.54 | 0.72 | -1.12 | 0.71 | -2.54 | *S. baicalensis* |

Table S1: Continued

| Number | Compound | OB | Caco-2 | DL | BBB | Log*P* | Log*S* | Herbs |
| --- | --- | --- | --- | --- | --- | --- | --- | --- |
| Molecule0357* | 8-methoxy-5-O-glucoside flavone_DG | 24.54 | 1.06 | 0.20 | 0.70 | 3.43 | -3.42 | *S. baicalensis* |
| Molecule0358 | 3,5,7,2',6'-Pentahydroxyflavone | 9.76 | 0.04 | 0.27 | -0.47 | 1.01 | -2.21 | *S. baicalensis* |
| Molecule0359 | Isocarthamidin-7-O-D-glucuronide | 24.87 | -1.26 | 0.79 | -1.95 | 1.21 | -2.22 | *S. baicalensis* |
| Molecule0360 | Dihydrohispidulin | 17.19 | 0.51 | 0.27 | -0.37 | 2.56 | -3.31 | *S. baicalensis* |
| Molecule0361 | 3,6,7,2',6'-pentahydroxyflavanone | 64.47 | -0.30 | 0.27 | -0.74 | 1.00 | -2.28 | *S. baicalensis* |
| Molecule0362 | 2',5,8,-trihydroxy-7-methoxyflavone | 27.22 | 0.75 | 0.27 | -0.07 | 2.51 | -3.12 | *S. baicalensis* |
| Molecule0363 | 5,7,2,5-tetrahydroxy-8,6-dimethoxyflavone | 23.71 | 0.34 | 0.24 | -0.59 | 2.16 | -3.19 | *S. baicalensis* |
| Molecule0364 | Acacetin | 34.93 | 0.62 | 0.35 | 0.00 | 2.95 | -3.51 | *S. baicalensis* |
| Molecule0365 | Carthamidin | 43.78 | 0.16 | 0.72 | -0.42 | 2.22 | -2.97 | *S. baicalensis* |
| Molecule0366 | Cedrene | 54.78 | 1.79 | 0.06 | 2.14 | 5.18 | -5.11 | *S. baicalensis, R. chuanxiong* |
| Molecule0367 | Chrysin | 22.16 | 0.70 | 0.10 | 0.01 | 2.85 | -3.14 | *S. baicalensis* |
| Molecule0368 | 2,6,2',4'-tetrahydroxy-6'-methoxychaleone | 1.32 | -0.03 | 0.22 | -0.32 | 2.71 | -3.62 | *S. baicalensis* |
| Molecule0369 | Dihydrobaicalin | 40.24 | 0.55 | 0.75 | 0.18 | 2.66 | -2.98 | *S. baicalensis* |
| Molecule0370 | Eriodictyol | 60.85 | 0.07 | 0.23 | -0.66 | 2.15 | -2.90 | *S. baicalensis, E. breviscapu* |
| Molecule0371 | Isoborneol | 86.98 | 1.27 | 0.06 | 1.52 | 2.83 | -2.28 | *S. baicalensis* |
| Molecule0372 | Isocarthamidin | 18.97 | 0.31 | 0.05 | -0.54 | 2.22 | -2.97 | *S. baicalensis* |
| Molecule0373 | Salvigenin | 43.46 | 0.85 | 0.02 | -0.03 | 1.95 | -3.87 | *S. baicalensis* |
| Molecule0374 | Tenaxin-I | 24.00 | 0.96 | 0.72 | 0.19 | 2.04 | -3.50 | *S. baicalensis* |
| Molecule0375 | Viscidulin I | 41.43 | 0.02 | 0.35 | -0.42 | 1.01 | -2.21 | *S. baicalensis* |
| Molecule0376 | Viscidulin II | 73.67 | 0.48 | 0.27 | -0.11 | 2.47 | -3.29 | *S. baicalensis* |
| Molecule0377 | 3',5,6',7-tetrahydroxy-2',8-dimethoxyflavone | 53.58 | 0.25 | 0.23 | -0.57 | 2.19 | -3.13 | *S. baicalensis* |
| Molecule0378 | Viscidulin III | 9.91 | 0.35 | 0.33 | -0.27 | 1.64 | -2.74 | *S. baicalensis* |
| Molecule0379 | Alpha-guaiene | 28.05 | 1.84 | 0.24 | 2.03 | 5.80 | -3.94 | *S. baicalensis* |
| Molecule0380 | Beta-patchoulene | 51.60 | 1.82 | 0.02 | 2.18 | 5.68 | -4.23 | *S. baicalensis* |
| Molecule0381 | Campesterol | 5.97 | 1.60 | 0.75 | 1.41 | 6.72 | -7.42 | *S. baicalensis* |
| Molecule0382 | Darendoside A | 6.18 | -1.77 | 0.05 | -2.27 | -1.27 | -1.68 | *S. baicalensis* |
| Molecule0383 | Darendoside B | 8.60 | -1.73 | 0.04 | -2.00 | -0.58 | -1.49 | *S. baicalensis* |
| Molecule0384 | 5,7,2',6'-tetrahydroxyflavone | 59.28 | 0.20 | 0.37 | -0.56 | 2.04 | -2.84 | *S. baicalensis* |
| Molecule0385 | Dihydrooroxylin A | 5.53 | 0.71 | 0.21 | 0.03 | 2.98 | -3.31 | *S. baicalensis* |
| Molecule0386 | Menthone | 57.79 | 1.35 | 0.24 | 1.70 | 2.65 | -2.77 | *S. baicalensis* |
| Molecule0387 | Oroxylin A | 45.59 | 0.77 | 0.44 | 0.13 | 3.01 | -3.34 | *S. baicalensis* |

Table S1: Continued

| Number | Compound | OB | Caco-2 | DL | BBB | Log*P* | Log*S* | Herbs |
| --- | --- | --- | --- | --- | --- | --- | --- | --- |
| Molecule0388 | Salidroside | 16.31 | -0.81 | 0.02 | -1.41 | -1.16 | -1.36 | *S. baicalensis* |
| Molecule0389 | Scutevurin | 2.64 | -1.10 | 0.33 | -2.13 | 1.21 | -2.22 | *S. baicalensis, E. breviscapu* |
| Molecule0390 | Skullcapflavone I | 18.68 | 0.84 | 0.24 | 0.31 | 2.67 | -3.49 | *S. baicalensis* |
| Molecule0391 | Syringaresinol | 3.29 | 0.52 | 0.29 | -0.32 | 2.23 | -3.94 | *S. baicalensis, E. breviscapu* |
| Molecule0392 | 5,7,4'-trihydroxy-8-methoxyflavone | 38.33 | 0.45 | 0.45 | -0.40 | 2.54 | -3.30 | *S. baicalensis* |
| Molecule0393 | Wogonin | 20.71 | 0.79 | 0.02 | 0.10 | 2.97 | -3.36 | *S. baicalensis* |
| Molecule0394 | Baicalein | 23.77 | 0.58 | 0.44 | -0.05 | 2.66 | -2.98 | *S. baicalensis, E. breviscapu* |
| Molecule0395 | 5,7,8-trihydroxyflavone | 20.38 | 0.59 | 0.27 | -0.16 | 2.59 | -2.92 | *S. baicalensis* |
| Molecule0396 | 5,8-dihydroxy-6,7-dimethoxyflavone | 44.41 | 0.96 | 0.23 | 0.09 | 2.71 | -3.40 | *S. baicalensis* |
| Molecule0397 | 5-methoxy-7-hydroxyflaconone | 45.49 | 0.82 | 0.29 | 0.26 | 3.38 | -3.45 | *S. baicalensis* |
| Molecule0398 | 1,2,5,6-tetrahydrotanshinone | 45.68 | 1.00 | 0.36 | 0.40 | 3.33 | -4.37 | *S. miltiorrhiza* |
| Molecule0399 | 1,2-cis-2-(3,4-dimethoxy-hydroxypheny1)-acrylic acid | 64.54 | 0.55 | 0.08 | -0.04 | 1.60 | -2.56 | *S. miltiorrhiza* |
| Molecule0400 | 1,2-dihydro-4,4-dimcthyi-11,12-dihydroxy-13-isopropyl anthracone | 28.88 | 1.18 | 0.29 | 0.80 | 4.34 | -4.25 | *S. miltiorrhiza* |
| Molecule0401 | 1,2-dihydrotan-shinqiunone | 42.57 | 1.07 | 0.36 | 0.46 | 3.65 | -3.99 | *S. miltiorrhiza* |
| Molecule0402 | 1,5-dihydroxy-3-methylanthraquinone | 18.87 | 0.61 | 0.21 | -0.22 | 3.07 | -3.29 | *S. miltiorrhiza* |
| Molecule0403 | 1-dehy-dromiltirone | 24.33 | 1.13 | 0.26 | 0.74 | 5.05 | -5.08 | *S. miltiorrhiza* |
| Molecule0404 | 1-heneicosyl formate | 15.79 | 1.38 | 0.26 | 0.75 | 9.60 | -7.32 | *S. miltiorrhiza* |
| Molecule0405 | 1-ketoisocryptotanshinone | 17.98 | 0.48 | 0.44 | -0.01 | 3.28 | -4.12 | *S. miltiorrhiza* |
| Molecule0406 | 2-isopropyl-8-methylphenanthrene-3,4-dione | 37.63 | 1.24 | 0.23 | 0.81 | 4.07 | -5.40 | *S. miltiorrhiza* |
| Molecule0407 | 2α,3α,19-trihydroxy urs-12-en-28-oic acid | 16.31 | -0.22 | 0.71 | -0.81 | 4.87 | -4.78 | *S. miltiorrhiza* |
| Molecule0408 | 2α,3α-dihydroxy urs-12-en-28-oic acid | 15.17 | 0.01 | 0.74 | -0.64 | 5.49 | -5.01 | *S. miltiorrhiza* |
| Molecule0409 | 3,4-dihydroxyphenyl ethanol ketone | 29.63 | -0.11 | 0.04 | -0.61 | 0.51 | -1.28 | *S. miltiorrhiza* |
| Molecule0410 | 3,7-dimethylocta-2,6-dien-1-yl formate | 60.37 | 0.66 | 0.03 | 0.47 | 2.23 | -2.16 | *S. miltiorrhiza* |
| Molecule0411 | 3-O-acetyloleanolicacid | 11.39 | 0.51 | 0.70 | -0.09 | 7.48 | -6.47 | *S. miltiorrhiza* |
| Molecule0412 | 3α-hydroxytanshinone IIa | 50.10 | 0.52 | 0.44 | 0.22 | 3.14 | -3.97 | *S. miltiorrhiza* |
| Molecule0413 | 3β-hydroxytanshinone IIa | 47.17 | 0.48 | 0.45 | -0.13 | 3.20 | -3.95 | *S. miltiorrhiza* |
| Molecule0414 | Cis-sabinenehydrate | 28.43 | 1.21 | 0.05 | 1.50 | 2.12 | -2.28 | *S. miltiorrhiza, R. chuanxiong* |

Table S1: Continued

| Number | Compound | OB | Caco-2 | DL | BBB | Log*P* | Log*S* | Herbs |
| --- | --- | --- | --- | --- | --- | --- | --- | --- |
| Molecule0415 | 4-hydroxy-1-vinyl carboxy-7-(3,4-dihydroxypheny1)benzo[b]furan | 77.75 | 0.19 | 0.31 | -0.89 | 2.87 | -3.35 | *S. miltiorrhiza* |
| Molecule0416 | 4-methylenemiltirone | 34.47 | 1.25 | 0.23 | 0.87 | 3.37 | -4.87 | *S. miltiorrhiza* |
| Molecule0417 | 5-(3-hydroxypropyl)-7-methoxy-2-(3-methoxy-4-hydroxyphenyl)-3-benzo[b]furancarbaldehyde | 15.35 | 0.35 | 0.40 | -0.73 | 3.24 | -3.78 | *S. miltiorrhiza* |
| Molecule0418 | 6-o-syringyl-8-o-acetyl shanzhiside methyl ester | 51.69 | -1.72 | 0.71 | -2.08 | 0.62 | -2.61 | *S. miltiorrhiza* |
| Molecule0419* | 6-o-syringyl-8-o-acetyl shanzhiside methyl ester_DG | 12.70 | -0.58 | 0.67 | -1.30 | 2.25 | -2.99 | *S. miltiorrhiza* |
| Molecule0420 | 7-carbonyl-12-hydroxy-dehydroabietane | 36.99 | 1.03 | 0.28 | 0.84 | 5.36 | -4.93 | *S. miltiorrhiza* |
| Molecule0421 | 7-oxoroyleanone2 | 15.42 | 0.48 | 0.34 | 0.04 | 3.17 | -4.30 | *S. miltiorrhiza* |
| Molecule0422 | 7β-hydroxy-8-13-abietadiene-11,12-dione | 26.25 | 0.41 | 0.31 | -0.06 | 3.47 | -3.88 | *S. miltiorrhiza* |
| Molecule0423 | 9-methyl lithospermate b | 3.01 | -1.01 | 0.39 | -2.01 | 3.93 | -4.72 | *S. miltiorrhiza* |
| Molecule0424 | 9-octadecen-1-ol | 27.27 | 1.38 | 0.11 | 0.99 | 7.91 | -6.76 | *S. miltiorrhiza* |
| Molecule0425 | Alanine | 86.91 | -0.24 | 0.01 | -0.59 | -3.05 | 0.70 | *S. miltiorrhiza* |
| Molecule0426 | Arginine | 62.31 | -0.47 | 0.03 | -0.97 | -3.49 | -1.88 | *S. miltiorrhiza* |
| Molecule0427 | Aspartate | 70.61 | -1.11 | 0.02 | -1.70 | -3.52 | 0.03 | *S. miltiorrhiza* |
| Molecule0428 | Cystine | 27.29 | -1.14 | 0.05 | -1.88 | -3.16 | -1.16 | *S. miltiorrhiza* |
| Molecule0429 | Formyltanshinone | 74.09 | 0.53 | 0.42 | -0.28 | 3.85 | -4.11 | *S. miltiorrhiza* |
| Molecule0430 | Germacrene D | 30.21 | 1.86 | 0.06 | 2.15 | 5.64 | -4.98 | *S. miltiorrhiza, R. chuanxiong, S. baicalensis* |
| Molecule0431 | Glutamate | 105.51 | -1.00 | 0.02 | -1.57 | -3.54 | -0.26 | *S. miltiorrhiza* |
| Molecule0432 | Glycine | 54.15 | -0.53 | 0.00 | -0.99 | -3.34 | 0.87 | *S. miltiorrhiza* |
| Molecule0433 | Histidine | 96.62 | -0.32 | 0.03 | -0.54 | -2.67 | -0.34 | *S. miltiorrhiza, U. rhynchophylla* |
| Molecule0434 | Hydroxymethylenetanshinquinone | 37.36 | 0.38 | 0.41 | -0.48 | 2.45 | -3.63 | *S. miltiorrhiza* |
| Molecule0435 | Isoimperatorin | 45.69 | 0.99 | 0.23 | 0.51 | 3.70 | -3.67 | *S. miltiorrhiza* |
| Molecule0436 | Isoleucine | 56.04 | 0.08 | 0.01 | -0.13 | -1.73 | -0.06 | *S. miltiorrhiza, G. biloba* |

Table S1: Continued

| Number | Compound | OB | Caco-2 | DL | BBB | Log*P* | Log*S* | Herbs |
| --- | --- | --- | --- | --- | --- | --- | --- | --- |
| Molecule0437 | Leucine | 62.71 | 0.15 | 0.01 | 0.03 | -1.82 | -0.27 | *S. miltiorrhiza* |
| Molecule0438 | Lithospermic acid b | 3.01 | -1.88 | 0.41 | -2.62 | 3.93 | -4.51 | *S. miltiorrhiza* |
| Molecule0439 | Lysine | 44.06 | -0.87 | 0.02 | -1.68 | -3.76 | -0.14 | *S. miltiorrhiza* |
| Molecule0440 | Methylenetanshinone | 42.36 | 1.03 | 0.36 | 0.46 | 3.12 | -4.05 | *S. miltiorrhiza* |
| Molecule0441 | Neo-przewaquinone a | 17.75 | 0.71 | 0.19 | -0.67 | 5.87 | -5.20 | *S. miltiorrhiza* |
| Molecule0442 | Phenylalanine | 51.40 | 0.24 | 0.04 | 0.04 | -1.35 | -1.60 | *S. miltiorrhiza* |
| Molecule0443 | Proline | 82.82 | 0.22 | 0.01 | 0.37 | -2.71 | 0.50 | *S. miltiorrhiza* |
| Molecule0444 | Przewalskin a | 37.11 | -0.26 | 0.65 | -0.69 | 1.99 | -2.97 | *S. miltiorrhiza* |
| Molecule0445 | Przewalskin b | 22.18 | 0.34 | 0.44 | 0.22 | 3.39 | -3.99 | *S. miltiorrhiza* |
| Molecule0446 | Przewalskin c | 18.97 | 1.08 | 0.35 | 0.69 | 5.81 | -4.38 | *S. miltiorrhiza* |
| Molecule0447 | Przewalskin d | 17.77 | 1.45 | 0.29 | 1.34 | 5.79 | -4.23 | *S. miltiorrhiza* |
| Molecule0448 | Przewaquinone a | 22.19 | 0.38 | 0.45 | -0.38 | 3.64 | -4.07 | *S. miltiorrhiza* |
| Molecule0449 | Przewaquinone b | 34.29 | 0.39 | 0.41 | -0.45 | 2.72 | -3.95 | *S. miltiorrhiza* |
| Molecule0450 | Przewaquinone c | 60.99 | 0.41 | 0.40 | -0.30 | 2.89 | -3.82 | *S. miltiorrhiza* |
| Molecule0451 | Przewaquinone d | 41.31 | -0.06 | 0.45 | -0.68 | 2.11 | -3.25 | *S. miltiorrhiza* |
| Molecule0452 | Przewaquinone f | 26.08 | -0.10 | 0.46 | -0.90 | 1.99 | -3.23 | *S. miltiorrhiza* |
| Molecule0453 | R-(+)-β-d-(3,4-dihydroxypheny1)-lactic acid | 59.42 | -0.29 | 0.06 | -0.60 | 0.77 | -1.47 | *S. miltiorrhiza* |
| Molecule0454 | Saloilenone | 8.44 | 1.37 | 0.43 | 1.04 | 4.95 | -6.00 | *S. miltiorrhiza* |
| Molecule0455 | Salvianolic acid B | 3.01 | -1.68 | 0.41 | -2.52 | 3.93 | -4.51 | *S. miltiorrhiza* |
| Molecule0456 | Salvianolic acid n | 1.46 | -0.44 | 0.83 | -1.52 | 2.74 | -4.60 | *S. miltiorrhiza* |
| Molecule0457 | Saprorthoquinone | 18.26 | 1.33 | 0.21 | 0.97 | 4.76 | -5.20 | *S. miltiorrhiza* |
| Molecule0458 | Sclareol | 49.97 | 0.84 | 0.21 | 0.51 | 4.39 | -5.42 | *S. miltiorrhiza* |
| Molecule0459 | Serine | 105.33 | -1.06 | 0.01 | -1.73 | -3.42 | 0.66 | *S. miltiorrhiza* |
| Molecule0460 | Tannin | 7.30 | -5.51 | 0.03 | -7.38 | 4.73 | -3.62 | *S. miltiorrhiza* |
| Molecule0461* | Tannin_DG | 49.23 | -0.78 | 0.26 | -1.61 | 2.28 | -3.10 | *S. miltiorrhiza* |
| Molecule0462 | Tanshinal dehyde | 57.72 | 0.53 | 0.45 | -0.07 | 3.80 | -4.21 | *S. miltiorrhiza* |
| Molecule0463 | Tanshinol a | 59.66 | 0.36 | 0.41 | -0.51 | 2.82 | -3.91 | *S. miltiorrhiza* |
| Molecule0464 | Tanshinol II | 63.17 | 0.55 | 0.56 | 0.11 | 3.31 | -4.51 | *S. miltiorrhiza* |
| Molecule0465 | Tanshinol I | 86.16 | 0.34 | 0.52 | -0.01 | 2.51 | -4.23 | *S. miltiorrhiza* |
| Molecule0466 | Threonine | 70.99 | -0.84 | 0.01 | -1.88 | -3.01 | 0.60 | *S. miltiorrhiza* |
| Molecule0467 | Tyrosine | 70.52 | -0.27 | 0.05 | -0.64 | -2.39 | -1.37 | *S. miltiorrhiza* |
| Molecule0468 | Ursolic acid | 17.46 | 0.63 | 0.75 | 0.04 | 6.35 | -5.89 | *S. miltiorrhiza, U. rhynchophylla* |

Table S1: Continued

| Number | Compound | OB | Caco-2 | DL | BBB | Log*P* | Log*S* | Herbs |
| --- | --- | --- | --- | --- | --- | --- | --- | --- |
| Molecule0469 | Valine | 65.05 | 0.01 | 0.01 | -0.03 | -2.29 | 0.26 | *S. miltiorrhiza* |
| Molecule0470 | Z-8-hexadecen-1-ol acetate2 | 40.00 | 1.38 | 0.14 | 1.14 | 7.38 | -6.81 | *S. miltiorrhiza* |
| Molecule0471 | Aethiopinone | 20.93 | 1.27 | 0.21 | 0.90 | 3.94 | -5.31 | *S. miltiorrhiza* |
| Molecule0472 | Arucadiol | 35.39 | 1.46 | 0.38 | 1.07 | 5.16 | -5.17 | *S. miltiorrhiza* |
| Molecule0473 | Baicalin | 13.16 | -1.05 | 0.23 | -1.97 | 0.55 | -2.23 | *S. baicalensis, S. miltiorrhiza* |
| Molecule0474 | Borneol | 83.54 | 1.21 | 0.05 | 1.43 | 2.83 | -2.28 | *S. miltiorrhiza* |
| Molecule0475 | Carnosol | 14.90 | 0.80 | 0.43 | 0.56 | 4.35 | -3.92 | *S. miltiorrhiza* |
| Molecule0476 | Corosolic acid | 19.40 | -0.08 | 0.74 | -0.74 | 5.49 | -5.01 | *S. miltiorrhiza* |
| Molecule0477 | Cryptotanshinone | 57.44 | 0.95 | 0.40 | 0.51 | 4.32 | -4.48 | *S. miltiorrhiza* |
| Molecule0478 | Cyclotetradecane | 27.72 | 1.80 | 0.06 | 2.11 | 7.43 | -6.52 | *S. miltiorrhiza* |
| Molecule0479 | Cynaroside | 6.85 | -1.24 | 0.78 | -2.51 | 0.00 | -2.40 | *S. miltiorrhiza* |
| Molecule0480 | Dan-shexinkum a | 24.93 | 0.24 | 0.30 | -0.45 | 2.22 | -3.86 | *S. miltiorrhiza* |
| Molecule0481 | Dan-shexinkum b | 48.09 | 0.83 | 0.26 | 0.18 | 3.29 | -4.44 | *S. miltiorrhiza* |
| Molecule0482 | Dan-shexinkum c | 22.71 | 0.75 | 0.21 | 0.03 | 2.30 | -3.95 | *S. miltiorrhiza* |
| Molecule0483 | Dan-shexinkum d | 38.88 | 0.67 | 0.55 | -0.15 | 3.03 | -4.49 | *S. miltiorrhiza* |
| Molecule0484 | Danshenspiroketallactone | 62.64 | 0.88 | 0.31 | 0.51 | 3.16 | -5.41 | *S. miltiorrhiza* |
| Molecule0485 | Danshenspiroketallactone II | 97.71 | 1.05 | 0.29 | 0.84 | 3.96 | -4.93 | *S. miltiorrhiza* |
| Molecule0486 | Dehydrouvaol | 16.45 | 0.86 | 0.77 | 0.21 | 5.88 | -5.68 | *S. miltiorrhiza* |
| Molecule0487 | Deoxyneocryptotanshinone | 54.51 | 0.85 | 0.29 | 0.24 | 3.94 | -4.49 | *S. miltiorrhiza* |
| Molecule0488 | DihydroisotanshinoneⅠ | 43.39 | 0.92 | 0.36 | 0.20 | 3.51 | -4.44 | *S. miltiorrhiza* |
| Molecule0489 | Dihydrotanshinlactone | 44.39 | 1.26 | 0.32 | 0.81 | 3.17 | -4.82 | *S. miltiorrhiza* |
| Molecule0490 | DihydrotanshinoneⅠ | 50.04 | 0.96 | 0.36 | 0.43 | 3.63 | -4.56 | *S. miltiorrhiza* |
| Molecule0491 | Diisopro-penyl methyl vinyl cyclohexane | 53.43 | 1.81 | 0.07 | 2.07 | 5.57 | -4.89 | *S. miltiorrhiza* |
| Molecule0492 | Dimetbyl lithosper-mate b | 3.01 | -0.76 | 0.38 | -1.73 | 4.25 | -4.90 | *S. miltiorrhiza* |
| Molecule0493 | Dimethyllithospermate | 2.66 | -0.71 | 0.73 | -1.53 | 3.71 | -4.88 | *S. miltiorrhiza* |
| Molecule0494 | Elemene | 27.28 | 1.85 | 0.06 | 2.13 | 5.40 | -4.71 | *S. miltiorrhiza, R. chuanxiong* |
| Molecule0495 | Epicedrol | 88.02 | 1.31 | 0.12 | 1.42 | 3.53 | -4.46 | *S. miltiorrhiza* |
| Molecule0496 | Epidanshenspiroketallactone | 73.56 | 0.90 | 0.31 | 0.61 | 2.63 | -3.99 | *S. miltiorrhiza* |
| Molecule0497 | Ethyl lithospermate | 26.37 | -0.08 | 0.82 | -0.74 | 5.24 | -5.28 | *S. miltiorrhiza* |
| Molecule0498 | Ferruginol | 36.07 | 1.63 | 0.25 | 1.54 | 6.56 | -5.74 | *S. miltiorrhiza* |
| Molecule0499 | Germacrene b | 17.49 | 1.89 | 0.06 | 2.12 | 5.87 | -3.51 | *S. miltiorrhiza* |
| Molecule0500 | Glutamic acid | 6.93 | -1.05 | 0.02 | -1.63 | -3.54 | -0.26 | *S. miltiorrhiza* |
| Molecule0501 | Heptacosane | 8.18 | 1.91 | 0.35 | 1.69 | 10.74 | -7.97 | *S. miltiorrhiza* |

Table S1: Continued

| Number | Compound | OB | Caco-2 | DL | BBB | Log*P* | Log*S* | Herbs |
| --- | --- | --- | --- | --- | --- | --- | --- | --- |
| Molecule0502 | Isocryptotanshinone | 60.08 | 0.93 | 0.39 | 0.34 | 4.18 | -4.42 | *S. miltiorrhiza* |
| Molecule0503 | Isoferulic acid | 67.69 | 0.50 | 0.06 | 0.08 | 1.56 | -2.35 | *S. miltiorrhiza* |
| Molecule0504 | Isosalvianolic acid c | 2.48 | -0.42 | 0.83 | -1.00 | 3.88 | -4.75 | *S. miltiorrhiza* |
| Molecule0505 | Isotanshinone IIb | 61.64 | 0.37 | 0.45 | -0.33 | 3.10 | -3.86 | *S. miltiorrhiza* |
| Molecule0506 | Isotanshinone IIa | 55.09 | 1.02 | 0.40 | 0.45 | 4.11 | -4.34 | *S. miltiorrhiza* |
| Molecule0507 | IsotanshinoneⅠ | 29.76 | 1.01 | 0.36 | 0.19 | 3.40 | -4.23 | *S. miltiorrhiza* |
| Molecule0508 | Lithospermic acid monomethyl ester | 2.67 | -1.05 | 0.75 | -1.81 | 3.48 | -4.62 | *S. miltiorrhiza* |
| Molecule0509 | Lithospermic acid | 2.67 | -1.38 | 0.76 | -2.49 | 3.58 | -4.41 | *S. miltiorrhiza* |
| Molecule0510 | Manool | 54.95 | 1.28 | 0.20 | 1.16 | 5.80 | -6.23 | *S. miltiorrhiza* |
| Molecule0511 | Methylrosmarinate | 1.37 | 0.00 | 0.37 | -0.72 | 2.64 | -4.30 | *S. miltiorrhiza* |
| Molecule0512 | Methyltanshinonate | 19.28 | 0.55 | 0.55 | 0.09 | 3.46 | -4.28 | *S. miltiorrhiza* |
| Molecule0513 | Microstegiol | 30.94 | 1.05 | 0.28 | 0.99 | 4.44 | -4.76 | *S. miltiorrhiza* |
| Molecule0514 | Miltiodiol | 38.84 | 1.11 | 0.29 | 0.75 | 4.34 | -4.25 | *S. miltiorrhiza* |
| Molecule0515 | Miltionone Ⅰ | 54.75 | 0.34 | 0.32 | -0.11 | 2.98 | -4.24 | *S. miltiorrhiza* |
| Molecule0516 | Miltionone II | 70.17 | 0.62 | 0.44 | 0.03 | 2.59 | -3.68 | *S. miltiorrhiza* |
| Molecule0517 | Miltipolone | 36.56 | 0.50 | 0.37 | 0.17 | 2.49 | -4.00 | *S. miltiorrhiza* |
| Molecule0518 | Miltirone i | 38.92 | 1.23 | 0.25 | 0.87 | 4.84 | -5.26 | *S. miltiorrhiza* |
| Molecule0519 | Miltirone II | 49.95 | 0.04 | 0.24 | -0.25 | 2.31 | -2.79 | *S. miltiorrhiza* |
| Molecule0520 | Neocryptotanshinone II | 44.51 | 0.76 | 0.23 | 0.16 | 3.24 | -3.97 | *S. miltiorrhiza* |
| Molecule0521 | Neocryptotanshinone | 62.85 | 0.35 | 0.32 | -0.13 | 2.78 | -3.87 | *S. miltiorrhiza* |
| Molecule0522 | Neryl acetate | 62.67 | 1.34 | 0.04 | 1.39 | 4.11 | -3.01 | *S. miltiorrhiza* |
| Molecule0523 | Norsalvioxide | 24.55 | 1.23 | 0.28 | 0.98 | 4.36 | -4.86 | *S. miltiorrhiza* |
| Molecule0524 | Nortanshinone | 40.00 | 0.48 | 0.37 | -0.27 | 3.18 | -3.70 | *S. miltiorrhiza* |
| Molecule0525 | Octadecanol | 12.66 | 1.36 | 0.11 | 1.12 | 8.27 | -6.71 | *S. miltiorrhiza* |
| Molecule0526 | Oleanolic acid | 29.02 | 0.59 | 0.76 | 0.07 | 7.09 | -5.75 | *S. miltiorrhiza, U. rhynchophylla* |
| Molecule0527 | Paramiltioic acid | 25.89 | 0.05 | 0.37 | -0.29 | 2.82 | -3.73 | *S. miltiorrhiza* |
| Molecule0528 | Potassium salvianolate d | 1.57 | -0.40 | 0.50 | -1.33 | 2.55 | -4.04 | *S. miltiorrhiza* |
| Molecule0529 | Prolithospermic acid | 72.35 | 0.10 | 0.31 | -0.75 | 2.38 | -3.86 | *S. miltiorrhiza* |
| Molecule0530 | Przewalskin | 34.62 | 1.21 | 0.28 | 1.06 | 4.36 | -4.86 | *S. miltiorrhiza* |
| Molecule0531 | Przewalskinic acid a | 20.33 | -0.69 | 0.41 | -1.33 | 2.71 | -3.53 | *S. miltiorrhiza* |
| Molecule0532 | Przewalskinone b | 81.61 | 0.53 | 0.27 | -0.35 | 2.95 | -3.52 | *S. miltiorrhiza* |
| Molecule0533 | Rosmarinicacid | 1.38 | -0.34 | 0.35 | -1.02 | 2.57 | -3.95 | *S. miltiorrhiza* |
| Molecule0534 | Salviacoccin | 14.02 | 0.11 | 0.63 | -0.30 | 2.17 | -3.45 | *S. miltiorrhiza* |
| Molecule0535 | Salvianic acid a | 78.22 | -0.27 | 0.06 | -0.62 | 0.77 | -1.47 | *S. miltiorrhiza* |

Table S1: Continued

| Number | Compound | OB | Caco-2 | DL | BBB | Log*P* | Log*S* | Herbs |
| --- | --- | --- | --- | --- | --- | --- | --- | --- |
| Molecule0536 | Salvianic acid c | 1.45 | -0.70 | 0.37 | -1.39 | 1.41 | -3.08 | *S. miltiorrhiza* |
| Molecule0537 | Salvianolic acid a | 2.96 | -0.56 | 0.70 | -1.62 | 3.58 | -4.76 | *S. miltiorrhiza* |
| Molecule0538 | Salvianolic acid c | 2.50 | -0.23 | 0.81 | -1.02 | 3.45 | -3.91 | *S. miltiorrhiza* |
| Molecule0539 | Salvianolic acid d | 1.57 | -0.78 | 0.50 | -1.73 | 2.55 | -4.04 | *S. miltiorrhiza* |
| Molecule0540 | Salvianolic acid e | 3.01 | -1.51 | 0.39 | -2.46 | 3.94 | -4.87 | *S. miltiorrhiza* |
| Molecule0541 | Salvianolic acid f | 1.39 | -0.08 | 0.26 | -0.77 | 2.68 | -4.16 | *S. miltiorrhiza* |
| Molecule0542 | Salvianolic acid g | 51.90 | -0.14 | 0.61 | -0.97 | 2.38 | -3.40 | *S. miltiorrhiza* |
| Molecule0543 | Salvianolic acid j | 57.18 | -0.83 | 0.72 | -2.14 | 3.65 | -4.53 | *S. miltiorrhiza* |
| Molecule0544 | Salvilenone Ⅰ | 38.74 | 1.13 | 0.23 | 0.77 | 3.82 | -3.77 | *S. miltiorrhiza* |
| Molecule0545 | Salviol | 24.31 | 0.94 | 0.28 | 0.42 | 5.35 | -4.63 | *S. miltiorrhiza* |
| Molecule0546 | Salviolone | 32.51 | 1.03 | 0.24 | 0.72 | 3.88 | -4.43 | *S. miltiorrhiza* |
| Molecule0547 | Salvipisone | 10.52 | 0.89 | 0.24 | 0.31 | 3.35 | -4.91 | *S. miltiorrhiza* |
| Molecule0548 | Shanzhiside methyl ester | 6.44 | -2.41 | 0.47 | -5.83 | -1.74 | -0.85 | *S. miltiorrhiza* |
| Molecule0549* | Shanzhiside methyl ester_DG | 109.77 | -1.05 | 0.12 | -3.18 | -0.93 | -0.32 | *S. miltiorrhiza* |
| Molecule0550 | Sugiol | 34.49 | 1.07 | 0.28 | 0.63 | 5.36 | -4.93 | *S. miltiorrhiza* |
| Molecule0551 | Tanshindiol a | 74.54 | 0.02 | 0.46 | -0.74 | 2.06 | -3.21 | *S. miltiorrhiza* |
| Molecule0552 | Tanshindiol b | 47.58 | 0.07 | 0.45 | -0.63 | 2.11 | -3.25 | *S. miltiorrhiza* |
| Molecule0553 | Tanshindiol c | 47.87 | -0.04 | 0.45 | -0.65 | 2.11 | -3.25 | *S. miltiorrhiza* |
| Molecule0554 | Tanshinlactone | 33.89 | 1.26 | 0.32 | 0.75 | 4.08 | -4.19 | *S. miltiorrhiza* |
| Molecule0555 | Tanshinone IIa | 20.32 | 1.04 | 0.40 | 0.70 | 4.10 | -4.45 | *S. miltiorrhiza* |
| Molecule0556 | Tanshinone IIb | 70.19 | 0.43 | 0.45 | -0.30 | 3.31 | -4.01 | *S. miltiorrhiza* |
| Molecule0557 | Tanshinone Ⅵ | 49.59 | 0.47 | 0.30 | -0.28 | 2.21 | -3.86 | *S. miltiorrhiza* |
| Molecule0558 | Tanshinonev | 6.26 | 1.41 | 0.38 | 1.03 | 5.16 | -5.17 | *S. miltiorrhiza* |
| Molecule0559 | TanshinoneⅠ | 29.32 | 1.04 | 0.36 | 0.53 | 3.38 | -4.38 | *S. miltiorrhiza* |
| Molecule0560 | Tigogenin | 13.16 | 0.84 | 0.81 | 0.25 | 4.52 | -7.10 | *S. miltiorrhiza* |
| Molecule0561 | Uvaol | 42.49 | 0.91 | 0.76 | 0.33 | 6.13 | -6.21 | *S. miltiorrhiza* |
| Molecule0562 | Vitamin e | 14.26 | 1.64 | 0.54 | 1.28 | 8.84 | -7.79 | *S. miltiorrhiza* |
| Molecule0563 | △1-dehydrotanshinone | 48.67 | 1.07 | 0.40 | 0.60 | 4.12 | -4.50 | *S. miltiorrhiza* |
| Molecule0564 | δ1-dehydromiltirone | 41.13 | 1.22 | 0.26 | 0.88 | 5.05 | -5.08 | *S. miltiorrhiza* |
| Molecule0565 | α-amyrin | 28.40 | 1.44 | 0.76 | 1.34 | 6.90 | -7.00 | *S. miltiorrhiza* |
| Molecule0566 | α-carypohyllene | 28.12 | 1.87 | 0.06 | 2.14 | 6.07 | -4.28 | *S. miltiorrhiza* |
| Molecule0567 | α-thujene | 52.13 | 1.81 | 0.04 | 2.14 | 4.07 | -2.92 | *S. miltiorrhiza* |
| Molecule0568 | β-cadinol | 37.76 | 1.29 | 0.09 | 1.34 | 3.40 | -4.05 | *S. miltiorrhiza* |
| Molecule0569 | β-chamigrene | 38.02 | 1.83 | 0.08 | 2.15 | 6.03 | -5.08 | *S. miltiorrhiza* |
| Molecule0570 | β-phellandrene | 45.47 | 1.82 | 0.02 | 2.04 | 3.98 | -3.60 | *S. miltiorrhiza* |

Table S1: Continued

| Number | Compound | OB | Caco-2 | DL | BBB | Log*P* | Log*S* | Herbs |
| --- | --- | --- | --- | --- | --- | --- | --- | --- |
| Molecule0571 | β-caryophyllene | 30.56 | 1.82 | 0.09 | 2.03 | 5.35 | -4.83 | *S. miltiorrhiza* |
| Molecule0572 | 1,2,3,4-tetrahydro-1,1,6-trimethyl-naphthalene | 29.53 | 1.92 | 0.06 | 2.08 | 5.24 | -5.25 | *E. herba* |
| Molecule0573 | 1-(2,6,6-trimethyl-1,3-cyclohexadien-1-yl)-2-Buten-1-one | 36.43 | 1.35 | 0.05 | 1.59 | 4.02 | -3.00 | *E. herba* |
| Molecule0574 | 2,3,4-trimethyl-5-phenyloxazolidine | 80.03 | 1.56 | 0.06 | 1.51 | 1.85 | -1.90 | *E. herba* |
| Molecule0575 | Bornyl acetate | 65.46 | 1.28 | 0.08 | 1.57 | 3.50 | -3.51 | *E. herba, R. chuanxiong* |
| Molecule0576 | Linalyl acetate | 24.42 | 1.43 | 0.04 | 1.52 | 4.03 | -3.51 | *E. herba, S. miltiorrhiza* |
| Molecule0577 | Isoledene | 54.47 | 1.83 | 0.10 | 2.15 | 4.98 | -4.18 | *E. herba* |
| Molecule0578 | Isopinocampheol | 53.95 | 1.21 | 0.06 | 1.36 | 2.22 | -2.22 | *E. herba* |
| Molecule0579 | Guaiazulene | 15.12 | 1.96 | 0.07 | 1.90 | 5.72 | -6.29 | *E. herba* |
| Molecule0580 | Triacontanol | 10.46 | 1.47 | 0.45 | 0.54 | 10.47 | -7.59 | *E. herba* |
| Molecule0581 | 2-pentadecanone | 7.41 | 1.47 | 0.06 | 1.63 | 6.26 | -6.08 | *E. herba* |
| Molecule0582 | (E)-2-methyl-2-penten-1-ol | 99.80 | 1.12 | 0.01 | 1.28 | 1.47 | -0.47 | *E. herba* |
| Molecule0583 | (E)-3-octen-1-yne | 44.97 | 1.85 | 0.01 | 2.03 | -1.31 | 1.08 | *E. herba* |
| Molecule0584 | (E)-cinnamaldehyde | 25.57 | 1.36 | 0.02 | 1.56 | 2.00 | -2.51 | *E. herba* |
| Molecule0585 | 1,2,3-trimethylcyclohexane | 52.94 | 1.78 | 0.02 | 2.23 | 4.06 | -4.47 | *E. herba* |
| Molecule0586 | 1,2-heptanediol | 36.51 | 0.45 | 0.01 | 0.47 | 1.06 | -0.34 | *E. herba* |
| Molecule0587 | 1,3,4-trimethyl-3-cyclohexene-1-carboxaldehyde | 56.63 | 1.39 | 0.03 | 1.67 | 2.31 | -1.89 | *E. herba* |
| Molecule0588 | 1,3-dimethylpyrazole | 50.63 | 1.52 | 0.01 | 1.77 | 0.57 | -1.29 | *E. herba* |
| Molecule0589 | 1-Phenyl-1,2-Propanedione | 35.93 | 0.92 | 0.03 | 0.93 | 0.94 | -2.21 | *E. herba* |
| Molecule0590 | 1-phenyltetrazoline-5-thione | 69.23 | 0.59 | 0.04 | 0.53 | 1.60 | -2.46 | *E. herba* |
| Molecule0591 | 2-octyldodecan-1-ol | 15.08 | 1.33 | 0.14 | 0.84 | 8.90 | -6.99 | *E. herba* |
| Molecule0592 | 1-heptanol | 21.45 | 1.12 | 0.01 | 1.17 | 2.53 | -1.70 | *E. herba* |
| Molecule0593 | 1-methyl-4-nitroso-benzene | 55.71 | 1.74 | 0.02 | 1.73 | 2.28 | -2.53 | *E. herba* |
| Molecule0594 | 1-octen-3-ol | 37.26 | 1.22 | 0.01 | 1.35 | 2.43 | -1.99 | *E. herba, S. baicalensis* |
| Molecule0595 | 1-ethoxy-2-propanol | 38.46 | 0.64 | 0.01 | -0.11 | 0.14 | 0.41 | *E. herba* |
| Molecule0596 | 2,3-dimethyl-1-pentene | 37.02 | 1.77 | 0.01 | 2.15 | 3.96 | -2.18 | *E. herba* |
| Molecule0597 | 2,3-dimethyl-2-pentene | 44.64 | 1.80 | 0.01 | 2.05 | 3.51 | -1.10 | *E. herba* |
| Molecule0598 | 2,3-dihydro-3,5-dihydroxy-6-methyl-4H-pyran-4-on | 53.97 | 0.04 | 0.03 | -0.03 | -1.16 | 0.42 | *E. herba* |
| Molecule0599 | 2,4-pentanediol | 50.30 | 0.24 | 0.01 | -0.26 | -0.34 | 0.58 | *E. herba* |

Table S1: Continued

| Number | Compound | OB | Caco-2 | DL | BBB | Log*P* | Log*S* | Herbs |
| --- | --- | --- | --- | --- | --- | --- | --- | --- |
| Molecule0600 | 2,6-dimethyl-1,3,5,7-octatetraene | 11.66 | 1.85 | 0.02 | 1.89 | 4.05 | -3.17 | *E. herba* |
| Molecule0601 | 3,3,6,9,9,10-hexamethyl-1-decene | 16.29 | 1.81 | 0.06 | 2.00 | 6.93 | -6.45 | *E. herba* |
| Molecule0602 | 2-methyl-butanol-1-ol | 84.22 | 0.49 | 0.01 | 0.54 | 0.04 | 0.45 | *E. herba* |
| Molecule0603 | 2-propenoic acid-3-phenyl | 20.11 | 0.94 | 0.03 | 0.94 | 2.38 | -2.38 | *E. herba* |
| Molecule0604 | 2-hexen-1-ol | 64.54 | 1.05 | 0.01 | 1.18 | 1.79 | -0.87 | *E. herba* |
| Molecule0605 | 2-methyl-butanol | 80.65 | 1.08 | 0.00 | 1.24 | 1.24 | -0.32 | *E. herba* |
| Molecule0606 | 2-methylcrotonaldehyde | 75.53 | 1.27 | 0.00 | 1.68 | 0.93 | -0.32 | *E. herba* |
| Molecule0607 | 2-methyl-3-pentanone | 73.75 | 1.26 | 0.01 | 1.73 | 1.45 | -0.80 | *E. herba* |
| Molecule0608 | 2-methylpropanal | 27.24 | 1.14 | 0.00 | 1.51 | 0.60 | -0.13 | *E. herba* |
| Molecule0609 | 2-caren-10-al | 44.74 | 1.37 | 0.05 | 1.63 | 2.16 | -2.22 | *E. herba* |
| Molecule0610 | 2-penten-1-ol | 69.59 | 1.03 | 0.00 | 1.20 | 1.20 | -0.20 | *E. herba* |
| Molecule0611 | 3,7,1,5-tetramethyl-2-Hexadecen-1-ol | 32.89 | 1.29 | 0.13 | 0.98 | 7.96 | -6.06 | *E. herba* |
| Molecule0612 | 2-pentenoic acid | 70.06 | 0.76 | 0.01 | 1.04 | 1.19 | -0.37 | *E. herba* |
| Molecule0613 | 2-octen-4-one | 51.96 | 1.36 | 0.01 | 1.50 | 2.57 | -1.91 | *E. herba* |
| Molecule0614 | 2-ethylidene-1,1-dimethyl-cyclopentane | 49.88 | 1.81 | 0.02 | 2.23 | 4.40 | -2.54 | *E. herba* |
| Molecule0615 | 3,5,5-trimethyl-2-cyclohexen-1-one | 44.98 | 1.28 | 0.03 | 1.61 | 1.90 | -1.86 | *E. herba* |
| Molecule0616 | 3,7-dimethyl-cyclopenta cyclooctene | 46.63 | 1.91 | 0.06 | 1.96 | 4.47 | -3.08 | *E. herba* |
| Molecule0617 | 3,8-menthadiene | 40.32 | 1.84 | 0.02 | 2.17 | 4.53 | -2.84 | *E. herba* |
| Molecule0618 | 3-methyl-butanol | 76.16 | 1.04 | 0.00 | 1.15 | 1.47 | -0.37 | *E. herba* |
| Molecule0619 | 3-methyl-2,4-hexadiene | 48.23 | 1.84 | 0.01 | 2.07 | 3.55 | -1.84 | *E. herba* |
| Molecule0620 | 3-methyl-4-hydroxynapthalene | 61.00 | 1.66 | 0.05 | 1.74 | 3.21 | -2.66 | *E. herba* |
| Molecule0621 | 3-methyl-6-(1-methylethyl-idene)-cyclohexene | 30.03 | 1.87 | 0.02 | 2.10 | 4.29 | -2.49 | *E. herba* |
| Molecule0622 | 3-methylbutanoic acid 3-methylbutyl ester | 17.89 | 1.23 | 0.03 | 1.32 | 3.38 | -2.78 | *E. herba* |
| Molecule0623 | 3-methyl phenanthrene | 17.11 | 1.93 | 0.11 | 1.84 | 5.07 | -6.26 | *E. herba* |
| Molecule0624 | 3-ethylxylene | 50.24 | 1.89 | 0.02 | 2.07 | 4.35 | -3.84 | *E. herba* |
| Molecule0625 | 4,6-dihydroxy-2-quinoline carboxylic acid | 48.01 | -0.08 | 0.09 | -0.75 | 1.39 | -1.53 | *E. herba* |
| Molecule0626 | 4-methyl-2-pentanone | 17.74 | 1.36 | 0.01 | 1.77 | 1.31 | -0.96 | *E. herba* |
| Molecule0627 | 4-methyl benzoyl chloride | 54.03 | 1.40 | 0.02 | 1.44 | 2.28 | -3.23 | *E. herba* |
| Molecule0628 | 4-hydroxy-2–quinoline carboxylic acid | 44.72 | 0.33 | 0.08 | -0.03 | 2.40 | -2.14 | *E. herba* |
| Molecule0629 | 5-methylhexanoic acid methyl ester | 48.64 | 1.23 | 0.02 | 1.41 | 2.76 | -2.17 | *E. herba* |
| Molecule0630 | 5-(hydroxymethyl)-2-Furancarboxaldehyde | 26.73 | 0.07 | 0.02 | 0.19 | -0.17 | -0.61 | *E. herba* |
| Molecule0631 | 6-methyl-2-heptanone | 39.99 | 1.34 | 0.01 | 1.57 | 2.38 | -1.95 | *E. herba* |

Table S1: Continued

| Number | Compound | OB | Caco-2 | DL | BBB | Log*P* | Log*S* | Herbs |
| --- | --- | --- | --- | --- | --- | --- | --- | --- |
| Molecule0632 | 3-undecyne | 20.68 | 1.93 | 0.02 | 1.96 | 4.73 | -4.53 | *E. herba* |
| Molecule0633 | 6-methyl-5-hepten-2-one | 26.36 | 1.35 | 0.01 | 1.52 | 2.01 | -1.55 | *E. herba* |
| Molecule0634 | E, E-2,4-decadienal | 51.01 | 1.40 | 0.02 | 1.49 | 3.81 | -3.61 | *E. herba* |
| Molecule0635 | β-cyclocitral | 40.00 | 1.39 | 0.03 | 1.76 | 2.96 | -2.08 | *E. herba* |
| Molecule0636 | Camphene | 45.23 | 1.81 | 0.05 | 2.13 | 3.94 | -3.33 | *E. herba, S. miltiorrhiza, R. chuanxiong* |
| Molecule0637 | γ-terpinene | 33.07 | 1.87 | 0.02 | 2.11 | 4.36 | -2.41 | *E. herba, S. miltiorrhiza, R. chuanxiong* |
| Molecule0638 | Methyl benzoate | 45.93 | 1.25 | 0.02 | 1.41 | 1.98 | -1.70 | *E. herba* |
| Molecule0639 | Isobutyl benzoate | 47.67 | 1.23 | 0.04 | 1.41 | 3.27 | -3.23 | *E. herba* |
| Molecule0640 | 4-hydroxy-7-methoxy-2-quinoline carboxylic acid | 1.08 | 0.89 | 0.05 | 0.97 | 0.91 | -0.44 | *E. herba* |
| Molecule0641 | Styrene | 29.55 | 1.86 | 0.01 | 2.00 | 2.92 | -3.00 | *E. herba* |
| Molecule0642 | Benzylmethylamine | 20.77 | 1.53 | 0.02 | 1.81 | 1.28 | -1.43 | *E. herba* |
| Molecule0643 | Alloocimene | 14.75 | 1.87 | 0.02 | 1.98 | 4.36 | -2.45 | *E. herba* |
| Molecule0644 | Butyrolactone | 76.91 | 1.03 | 0.01 | 1.43 | -0.11 | 0.44 | *E. herba* |
| Molecule0645 | P-cymenol | 33.14 | 1.33 | 0.03 | 1.37 | 2.53 | -2.09 | *E. herba, R. chuanxiong* |
| Molecule0646 | P-mentha-1,3-dien-7-al | 41.71 | 1.40 | 0.03 | 1.65 | 2.99 | -2.18 | *E. herba* |
| Molecule0647 | 5-hepten-2-ol | 31.76 | 1.31 | 0.03 | 1.46 | 3.69 | -2.23 | *E. herba* |
| Molecule0648 | P-vinylanisole | 49.23 | 1.67 | 0.02 | 1.85 | 2.65 | -2.81 | *E. herba* |
| Molecule0649 | Cumic aldehyde | 38.19 | 1.41 | 0.03 | 1.53 | 2.73 | -3.01 | *E. herba* |
| Molecule0650 | Cumic alcohol | 41.92 | 1.20 | 0.03 | 1.24 | 2.63 | -2.36 | *E. herba* |
| Molecule0651 | Dibenzofuran | 37.28 | 1.78 | 0.08 | 1.68 | 3.92 | -3.62 | *E. herba* |
| Molecule0652 | Xylene | 48.74 | 1.88 | 0.01 | 1.98 | 3.15 | -2.72 | *E. herba* |
| Molecule0653 | Dimethylstyrene | 39.65 | 1.90 | 0.02 | 2.02 | 3.76 | -2.77 | *E. herba* |
| Molecule0654 | Dimethyl naphthalene | 38.03 | 1.93 | 0.05 | 1.90 | 4.37 | -4.52 | *E. herba* |
| Molecule0655 | Dichloromethane | 7.57 | 1.80 | 0.00 | 2.41 | 1.12 | -0.69 | *E. herba* |
| Molecule0656 | 6,10-dimethyl-undecan-2-one | 17.27 | 1.36 | 0.04 | 1.40 | 4.96 | -5.26 | *E. herba* |
| Molecule0657 | Trans-2-nonenal | 19.17 | 1.35 | 0.02 | 1.61 | 3.56 | -3.18 | *E. herba* |
| Molecule0658 | Phellandral | 40.36 | 1.36 | 0.03 | 1.61 | 2.90 | -2.71 | *E. herba, R. chuanxiong* |
| Molecule0659 | Methyl furoate | 48.30 | 1.10 | 0.02 | 1.18 | 1.21 | -0.77 | *E. herba* |
| Molecule0660 | Heptanoicacid | 13.37 | 0.93 | 0.01 | 1.00 | 2.41 | -1.64 | *E. herba* |

Table S1: Continued

| Number | Compound | OB | Caco-2 | DL | BBB | Log*P* | Log*S* | Herbs |
| --- | --- | --- | --- | --- | --- | --- | --- | --- |
| Molecule0661 | Heptanoic acid methyl ester | 19.46 | 1.20 | 0.02 | 1.34 | 2.96 | -2.37 | *E. herba* |
| Molecule0662 | Cinnamic acid | 19.83 | 0.92 | 0.03 | 0.94 | 2.38 | -2.38 | *E. herba* |
| Molecule0663 | Piperitone | 54.07 | 1.27 | 0.03 | 1.55 | 2.47 | -2.05 | *E. herba* |
| Molecule0664 | 1, 2-benznendicarboxylicacid-bis(2- methoxyethyl)ester | 125.80 | -0.02 | 0.15 | 0.15 | 1.48 | -3.04 | *E. herba* |
| Molecule0665 | Toluene | 42.58 | 1.84 | 0.01 | 2.08 | 2.56 | -2.26 | *E. herba* |
| Molecule0666 | Methylacetophenone | 22.26 | 1.45 | 0.03 | 1.52 | 2.71 | -2.93 | *E. herba* |
| Molecule0667 | Formic acid | 33.26 | 0.21 | 0.00 | 0.53 | -0.47 | 1.02 | *E. herba* |
| Molecule0668 | Diethylsulfate | 72.36 | 0.93 | 0.02 | 0.66 | -0.29 | -0.90 | *E. herba* |
| Molecule0669 | Ephedroxane | 2.10 | 1.20 | 0.06 | 1.28 | 2.01 | -1.49 | *E. herba* |
| Molecule0670 | Ephedrine | 45.21 | 1.02 | 0.03 | 1.11 | 1.00 | -1.30 | *E. herba* |
| Molecule0671 | Decenoic acid ethyl ester | 19.45 | 1.30 | 0.04 | 1.31 | 4.98 | -4.45 | *E. herba* |
| Molecule0672 | Apigenin-5-rhamnoside | 47.87 | 0.41 | 0.00 | 0.71 | -0.12 | 0.73 | *E. herba* |
| Molecule0673 | Norephedrine | 66.83 | 0.56 | 0.03 | 0.54 | 0.57 | -0.87 | *E. herba* |
| Molecule0674 | Norpseudoephedrine | 74.13 | 0.53 | 0.03 | 0.54 | 0.57 | -0.87 | *E. herba* |
| Molecule0675 | Ligustrazine | 29.64 | 1.19 | 0.03 | 1.06 | 1.46 | -0.96 | *E. herba, R. chuanxiong* |
| Molecule0676 | Myrtenol | 48.76 | 1.15 | 0.06 | 1.29 | 2.71 | -1.96 | *E. herba* |
| Molecule0677 | Pseudoephedrine | 40.82 | 1.10 | 0.03 | 1.23 | 1.00 | -1.30 | *E. herba* |
| Molecule0678 | Sabinene | 44.89 | 1.81 | 0.04 | 2.19 | 3.04 | -3.33 | *E. herba, R. chuanxiong* |
| Molecule0679 | Decenoic acid methyl ester | 19.29 | 1.30 | 0.03 | 1.43 | 4.50 | -4.12 | *E. herba* |
| Molecule0680 | Carvacrol | 43.71 | 1.62 | 0.03 | 1.76 | 3.20 | -2.50 | *E. herba* |
| Molecule0681 | Carvacrol methyl ether | 30.50 | 1.68 | 0.04 | 1.84 | 4.10 | -3.69 | *E. herba* |
| Molecule0682 | Carvenone | 51.37 | 1.36 | 0.03 | 1.59 | 2.29 | -2.13 | *E. herba* |
| Molecule0683 | Octanol | 21.75 | 1.25 | 0.01 | 1.48 | 3.01 | -2.21 | *E. herba* |
| Molecule0684 | Ethanol | 63.21 | 0.76 | 0.00 | 0.50 | -0.40 | 1.10 | *E. herba* |
| Molecule0685 | Acetic acid | 47.87 | 0.41 | 0.00 | 0.71 | -0.12 | 0.73 | *E. herba* |
| Molecule0686 | Methyl acetate | 40.17 | 0.98 | 0.00 | 1.32 | 0.18 | 0.54 | *E. herba* |
| Molecule0687 | Acetic acid,ethyl ester | 45.02 | 1.07 | 0.00 | 1.26 | 0.74 | 0.14 | *E. herba* |
| Molecule0688 | E, E-farnesylacetone | 13.25 | 1.60 | 0.10 | 1.53 | 6.20 | -4.46 | *E. herba* |
| Molecule0689 | Ethane | 55.22 | 1.72 | 0.00 | 2.24 | 1.44 | 0.38 | *E. herba* |
| Molecule0690 | Isobutyric acid | 75.37 | 0.71 | 0.01 | 1.00 | 0.78 | 0.35 | *E. herba* |
| Molecule0691 | Camphor | 67.30 | 1.29 | 0.05 | 1.71 | 2.85 | -2.24 | *E. herba* |
| Molecule0692 | Perillaldehyde | 39.58 | 1.37 | 0.03 | 1.63 | 2.97 | -2.41 | *E. herba* |
| Molecule0693 | N-methylephedrine | 37.82 | 1.03 | 0.04 | 1.28 | 1.73 | -1.07 | *E. herba* |

Table S1: Continued

| Number | Compound | OB | Caco-2 | DL | BBB | Log*P* | Log*S* | Herbs |
| --- | --- | --- | --- | --- | --- | --- | --- | --- |
| Molecule0694 | O-benzoyl-L-(+)-pseudoephedrine | 65.17 | 0.99 | 0.13 | 0.76 | 3.13 | -4.37 | *E. herba* |
| Molecule0695 | Trans-ionone | 40.60 | 1.37 | 0.05 | 1.53 | 4.14 | -3.59 | *E. herba* |
| Molecule0696 | α-bergamotene | 16.73 | 1.86 | 0.09 | 1.99 | 5.91 | -4.48 | *E. herba, R. chuanxiong* |
| Molecule0697 | 1,4-cineole | 61.03 | 1.55 | 0.04 | 1.92 | 2.31 | -3.43 | *E. herba* |
| Molecule0698 | β-lonone | 20.27 | 1.42 | 0.05 | 1.49 | 4.11 | -3.27 | *E. herba, G. biloba* |
| Molecule0699 | δ-cadinene | 16.85 | 1.85 | 0.08 | 2.10 | 4.92 | -3.69 | *E. herba, S. miltiorrhiza* |
| Molecule0700 | α-calacorene | 13.31 | 1.92 | 0.08 | 1.87 | 5.47 | -5.01 | *E. herba* |
| Molecule0701 | Leucopelargonidin | 58.03 | -0.14 | 0.24 | -0.62 | 0.62 | -2.20 | *E. herba* |
| Molecule0702 | Ethanone | 37.05 | -0.90 | 0.19 | -1.37 | -0.67 | -1.47 | *E. herba* |
| Molecule0703* | Ethanone_DG | 31.20 | 0.88 | 0.03 | 0.67 | 1.62 | -1.43 | *E. herba* |
| Molecule0704 | Herbacetin | 37.54 | 0.13 | 0.27 | -0.74 | 1.08 | -2.37 | *E. herba* |
| Molecule0705 | Calamene | 17.57 | 1.87 | 0.08 | 2.04 | 5.60 | -6.11 | *E. herba* |
| Molecule0706 | 1,5,8-trimethyl-1,2-dihydro-naphthalene | 53.88 | 1.91 | 0.06 | 2.00 | 4.96 | -4.91 | *E. herba* |
| Molecule0707 | Clovene | 46.18 | 1.81 | 0.11 | 2.07 | 5.12 | -5.81 | *E. herba* |
| Molecule0708 | Dihydro-β-ionone | 19.27 | 1.46 | 0.05 | 1.56 | 3.86 | -3.20 | *E. herba* |
| Molecule0709 | Dihydrocarveol | 53.80 | 1.32 | 0.03 | 1.63 | 2.70 | -2.07 | *E. herba* |
| Molecule0710 | Nonacosanol | 10.57 | 1.47 | 0.43 | 0.70 | 10.35 | -7.55 | *E. herba* |
| Molecule0711 | Trichloroeicosyl- silane | 7.04 | 2.01 | 0.23 | 2.15 | 9.93 | -8.13 | *E. herba* |
| Molecule0712 | 1,5-dimethyl-4-(2-hexenyl)benzene | 25.66 | 1.96 | 0.05 | 2.02 | 5.73 | -5.68 | *E. herba* |
| Molecule0713 | Eicosanoic acid | 16.66 | 1.18 | 0.19 | 1.09 | 8.53 | -6.80 | *E. herba* |
| Molecule0714 | D-menth-2-en-7-ol | 47.34 | 1.17 | 0.03 | 1.37 | 2.74 | -2.83 | *E. herba* |
| Molecule0715 | Trans, trans-farnesol | 33.81 | 1.31 | 0.06 | 1.02 | 4.84 | -3.58 | *E. herba* |
| Molecule0716 | Trans-caryophyllene | 29.50 | 1.87 | 0.09 | 2.13 | 5.35 | -4.83 | *E. herba* |
| Molecule0717 | Delphinidin | 17.05 | -0.06 | 0.28 | -0.74 | -0.16 | -1.68 | *E. herba* |
| Molecule0718 | Cresol | 51.99 | 1.56 | 0.01 | 1.88 | 1.95 | -0.67 | *E. herba* |
| Molecule0719 | Methylephedrine | 32.87 | 1.04 | 0.04 | 1.37 | 1.73 | -1.07 | *E. herba* |
| Molecule0720 | Methylpseudoephedrine | 40.34 | 1.12 | 0.04 | 1.37 | 1.73 | -1.07 | *E. herba* |
| Molecule0721 | Diethyl phthalate | 64.54 | 0.79 | 0.13 | 0.57 | 2.60 | -2.76 | *E. herba* |
| Molecule0722 | Patchoulane | 52.71 | 1.79 | 0.11 | 2.19 | 5.55 | -6.02 | *E. herba* |
| Molecule0723 | 1-propenyl-2-vinyl-4-methylcycloheptane | 35.13 | 1.84 | 0.04 | 2.10 | 5.45 | -5.35 | *E. herba* |
| Molecule0724 | Geranyl acetate | 26.14 | 1.28 | 0.04 | 1.29 | 4.11 | -3.01 | *E. herba* |

Table S1: Continued

| Number | Compound | OB | Caco-2 | DL | BBB | Log*P* | Log*S* | Herbs |
| --- | --- | --- | --- | --- | --- | --- | --- | --- |
| Molecule0725 | Roseoxide | 37.82 | 1.51 | 0.03 | 1.85 | 3.30 | -2.62 | *E. herba* |
| Molecule0726 | Geraniol | 23.91 | 1.19 | 0.02 | 1.14 | 2.89 | -2.05 | *E. herba* |
| Molecule0727 | Nonanoic acid | 40.51 | 0.92 | 0.02 | 1.08 | 3.47 | -2.75 | *E. herba* |
| Molecule0728 | Isopropyl myristate | 21.01 | 1.42 | 0.11 | 1.33 | 7.02 | -6.39 | *E. herba* |
| Molecule0729 | Octadecanoic acid methyl ester | 16.80 | 1.41 | 0.16 | 1.29 | 8.45 | -6.94 | *E. herba* |
| Molecule0730 | 1-terpineol | 49.71 | 1.22 | 0.03 | 1.34 | 2.91 | -1.90 | *E. herba, R. chuanxiong* |
| Molecule0731 | Methyl dodecanoate | 26.42 | 1.30 | 0.05 | 1.28 | 5.51 | -5.25 | *E. herba* |
| Molecule0732 | Cis-piperitol | 37.53 | 1.26 | 0.03 | -0.74 | 2.64 | -1.90 | *E. herba, R. chuanxiong* |
| Molecule0733 | 2,2,6-trimethyl-6-vinyl-tetra-hydropyran | 36.85 | 1.57 | 0.03 | 2.01 | 3.35 | -3.89 | *E. herba* |
| Molecule0734 | Terpineol | 32.59 | 1.41 | 0.03 | 1.75 | 3.17 | -2.00 | *E. herba, R. chuanxiong* |
| Molecule0735 | Leucocyanidin | 41.03 | -0.30 | 0.27 | -0.95 | 0.44 | -2.14 | *E. herba* |
| Molecule0736 | Citronellol | 36.76 | 1.18 | 0.02 | 1.13 | 3.48 | -2.27 | *E. herba* |
| Molecule0737 | Octanoic acid methyl ester | 18.80 | 1.26 | 0.02 | 1.38 | 3.54 | -3.03 | *E. herba* |
| Molecule0738 | Neocnidilide | 64.88 | 1.27 | 0.07 | 1.39 | 3.28 | -2.82 | *E. herba, R. chuanxiong* |
| Molecule0739 | Cedreneoxide | 82.86 | 1.56 | 0.14 | 1.80 | 3.25 | -5.28 | *E. herba* |
| Molecule0740 | Ginsenoside CK | 6.51 | -3.39 | 0.04 | -0.44 | 3.73 | -4.62 | *P. notoginseng* |
| Molecule0741 | (+)-maalioxide | 55.93 | 1.59 | 0.13 | 1.93 | 4.18 | -5.34 | *P. ginseng* |
| Molecule0742 | 1,2-Benzenedicarboxylic acid diisooctyl ester | 43.59 | 0.87 | 0.78 | 0.33 | 7.02 | -6.58 | *P. ginseng* |
| Molecule0743 | 1-heptadecanol | 12.97 | 1.31 | 0.13 | 0.98 | 7.82 | -6.55 | *P. ginseng* |
| Molecule0744 | 1-hexadecyne | 15.31 | 1.91 | 0.08 | 1.90 | 8.17 | -6.77 | *P. ginseng* |
| Molecule0745 | 1-tetradecanol | 14.19 | 1.25 | 0.22 | 1.06 | 6.21 | -5.68 | *P. ginseng* |
| Molecule0746 | 12-O-nicotinoylisolineolone | 21.71 | -0.53 | 0.04 | -1.14 | 1.84 | -3.66 | *P. ginseng* |
| Molecule0747 | 13-tetradecen-1-ol acetate | 36.76 | 1.36 | 0.81 | 1.06 | 6.38 | -6.29 | *P. ginseng* |
| Molecule0748 | 16-Oxoseratenediol | 15.11 | 0.40 | 0.60 | -0.25 | 5.17 | -5.30 | *P. ginseng* |
| Molecule0749 | 2,2-dimethylbutane | 37.81 | 1.78 | 0.39 | 2.18 | 3.74 | -2.97 | *P. ginseng* |
| Molecule0750 | 2,3,4-trimethyldecane | 16.15 | 1.82 | 0.57 | 2.08 | 5.42 | -6.05 | *P. ginseng* |
| Molecule0751 | 2,3,8-trimethyldecane | 5.51 | 1.79 | 0.78 | 1.94 | 6.75 | -6.07 | *P. ginseng* |
| Molecule0752 | 2,6,10,15-tetramethylheptadecane | 3.81 | 1.84 | 0.77 | 1.85 | 9.38 | -7.74 | *P. ginseng* |
| Molecule0753 | 2-methyltridecane | 17.62 | 1.81 | 0.05 | 1.89 | 7.90 | -6.58 | *P. ginseng* |
| Molecule0754 | 20(R)-ginsenoside-Rh1 | 3.78 | -1.17 | 0.01 | -2.14 | 2.68 | -3.93 | *P. ginseng* |

Table S1: Continued

| Number | Compound | OB | Caco-2 | DL | BBB | Log*P* | Log*S* | Herbs |
| --- | --- | --- | --- | --- | --- | --- | --- | --- |
| Molecule0755 | 20(S)-ginsenoside-Rh1 | 3.86 | -1.10 | 0.07 | -2.04 | 2.68 | -3.93 | *P. ginseng* |
| Molecule0756 | 20(S)-ginsenoside-Rg2 | 10.09 | -1.93 | 0.16 | -2.78 | 1.88 | -3.66 | *P. ginseng* |
| Molecule0757 | 20(S)-protopanaxadiol | 29.69 | 0.56 | 0.04 | -0.07 | 5.36 | -5.12 | *P. ginseng* |
| Molecule0758 | 20-(R)-ginsenoside-Rg2 | 10.09 | -1.96 | 0.33 | -2.95 | 1.88 | -3.66 | *P. ginseng* |
| Molecule0759 | 20(S)-ginsenoside-Rg3 | 13.69 | -1.65 | 0.09 | -2.47 | 2.27 | -3.90 | *P. ginseng* |
| Molecule0760 | 20-hexadecanoylingenol | 28.20 | 0.44 | 0.07 | 0.19 | 6.46 | -6.05 | *P. ginseng* |
| Molecule0761 | 2,6-dimethyl-3,7-octadiene-2,6-diol | 39.01 | 0.51 | 0.03 | 0.60 | 1.62 | -1.87 | *P. ginseng* |
| Molecule0762 | 3,4-dimethylheptane | 46.67 | 1.76 | 0.10 | 2.03 | 4.95 | -4.70 | *P. ginseng* |
| Molecule0763 | 3,5-dimethyl-4-methoxybenzoic acid | 61.69 | 0.75 | 0.04 | 0.56 | 2.18 | -2.28 | *P. ginseng* |
| Molecule0764 | 3-O-beta-D-glucuronopyranosyl gypsogenin | 8.67 | -0.78 | 0.01 | -1.37 | 4.25 | -4.78 | *P. ginseng* |
| Molecule0765 | 3-ethyl-3-methylheptane | 37.33 | 1.79 | 0.75 | 2.13 | 5.80 | -4.92 | *P. ginseng* |
| Molecule0766 | 3-methylheptane | 37.10 | 1.78 | 0.05 | 2.15 | 4.82 | -4.38 | *P. ginseng* |
| Molecule0767 | 3-methylundecane | 6.57 | 1.79 | 0.03 | 1.96 | 6.85 | -5.95 | *P. ginseng* |
| Molecule0768 | 4-methyldodecane | 6.39 | 1.81 | 0.78 | 2.06 | 7.41 | -6.28 | *P. ginseng* |
| Molecule0769 | 5-(2-methylpropyl)nonane | 14.57 | 1.81 | 0.78 | 2.10 | 7.29 | -6.21 | *P. ginseng* |
| Molecule0770 | 5-(heptadec-12-enyl) resorcinol | 3.29 | 1.38 | 0.12 | 0.79 | 8.74 | -6.32 | *P. ginseng* |
| Molecule0771 | 5-methyl-tetradecane | 4.57 | 1.84 | 0.01 | 2.04 | 8.27 | -6.84 | *P. ginseng* |
| Molecule0772 | 6"-malonylginsenoside Rd1 | 6.70 | -4.21 | 0.78 | -5.39 | -0.63 | -3.27 | *P. ginseng* |
| Molecule0773 | 7-(beta-xylosyl)cephalomannine | 27.33 | -1.62 | 0.78 | -2.25 | 2.87 | -4.49 | *P. ginseng* |
| Molecule0774 | 7-tetradecyne | 20.07 | 1.94 | 0.01 | 1.84 | 6.76 | -5.13 | *P. ginseng* |
| Molecule0775 | 7alpha-L-rhamnosyl-6-methoxylutcolin | 12.66 | -0.71 | 0.77 | -1.99 | 0.89 | -2.55 | *P. ginseng* |
| Molecule0776* | 7alpha-L-rhamnosyl-6-methoxylutcolin_DG | 37.13 | 0.37 | 0.20 | -0.78 | 2.24 | -3.17 | *P. ginseng* |
| Molecule0777 | Acetal | 26.40 | 1.25 | 0.83 | 1.46 | 1.19 | -0.64 | *P. ginseng* |
| Molecule0778 | Adenosine triphosphate | 8.23 | -3.10 | 0.13 | -3.75 | -0.87 | -2.04 | *P. ginseng* |
| Molecule0779 | Alloaromadendrene | 53.02 | 1.82 | 0.02 | 2.13 | 3.70 | -4.94 | *P. ginseng, E. herba* |
| Molecule0780 | Aposiopolamine | 66.65 | 0.66 | 0.05 | 0.40 | 1.52 | -3.03 | *P. ginseng* |
| Molecule0781 | Araloside A | 17.01 | -3.33 | 0.03 | -3.74 | 2.44 | -3.89 | *P. ginseng* |
| Molecule0782* | Araloside A_DG | 14.36 | 0.60 | 0.03 | 0.10 | 7.09 | -5.75 | *P. ginseng* |
| Molecule0783 | Argininyl-fructosyl-glucose | 0.74 | -3.12 | 0.13 | -3.75 | -3.41 | -1.34 | *P. ginseng* |
| Molecule0784* | Argininyl-fructosyl-glucose_DG | 11.24 | -1.74 | 0.04 | -2.30 | -3.04 | -2.03 | *P. ginseng* |
| Molecule0785 | Bicylogermacrene | 28.00 | 1.85 | 0.77 | 2.11 | 5.09 | -4.39 | *P. ginseng, R. chuanxiong* |
| Molecule0786 | Biotin | 75.75 | -0.04 | 0.02 | -0.28 | 0.17 | -2.30 | *P. ginseng* |

Table S1: Continued

| Number | Compound | OB | Caco-2 | DL | BBB | Log*P* | Log*S* | Herbs |
| --- | --- | --- | --- | --- | --- | --- | --- | --- |
| Molecule0787 | Campesteryl ferulate | 22.10 | 1.12 | 0.04 | 0.17 | 7.93 | -7.89 | *P. ginseng* |
| Molecule0788 | Celabenzine | 20.75 | 0.77 | 0.28 | 0.05 | 2.30 | -4.34 | *P. ginseng* |
| Molecule0789 | Chrysanthemaxanthin | 38.72 | 0.49 | 0.04 | -0.99 | 8.13 | -5.87 | *P. ginseng* |
| Molecule0790 | Citronellal | 50.78 | 1.38 | 0.01 | 1.61 | 3.25 | -2.63 | *P. ginseng* |
| Molecule0791 | D-mannuronic acid | 56.17 | -2.13 | 0.68 | -4.77 | -2.30 | 0.18 | *P. ginseng* |
| Molecule0792 | Dauricine | 23.65 | 0.96 | 0.77 | 0.10 | 5.97 | -5.45 | *P. ginseng* |
| Molecule0793 | Deoxyharringtonine | 39.27 | 0.19 | 0.31 | -0.25 | 2.96 | -3.95 | *P. ginseng* |
| Molecule0794 | Dianthramine | 41.10 | -0.22 | 0.02 | -0.97 | 2.62 | -3.00 | *P. ginseng* |
| Molecule0795 | Dodecanol | 18.42 | 1.25 | 0.77 | 1.15 | 5.36 | -4.86 | *P. ginseng* |
| Molecule0796 | Eicosanetetraenoic acid | 45.57 | 1.27 | 0.04 | 0.58 | 6.80 | -6.30 | *P. ginseng* |
| Molecule0797 | Elemicin | 24.77 | 1.45 | 0.31 | 1.34 | 3.03 | -3.16 | *P. ginseng* |
| Molecule0798 | Folinic acid | 23.60 | -1.74 | 0.77 | -2.59 | 0.44 | -2.95 | *P. ginseng* |
| Molecule0799 | Frutinone A | 66.05 | 0.89 | 0.34 | 0.46 | 2.80 | -2.81 | *P. ginseng* |
| Molecule0800 | Gamma-selinene | 22.58 | 1.83 | 0.79 | 2.10 | 5.27 | -4.09 | *P. ginseng* |
| Molecule0801 | Gamma-sitosterol | 36.91 | 1.43 | 0.77 | 1.16 | 7.27 | -7.35 | *P. ginseng* |
| Molecule0802 | Ginsenoside La | 10.07 | -1.90 | 0.04 | -3.25 | 1.99 | -4.07 | *P. ginseng* |
| Molecule0803 | Ginsenoside Ra0 | 7.30 | -5.56 | 0.30 | -6.76 | -0.71 | -2.65 | *P. ginseng* |
| Molecule0804 | Ginsenoside Ra1 | 7.09 | -4.51 | 0.83 | -6.41 | -0.70 | -2.90 | *P. ginseng* |
| Molecule0805 | Ginsenoside Ra2 | 7.62 | -4.43 | 0.07 | -6.28 | -0.82 | -2.87 | *P. ginseng* |
| Molecule0806 | Ginsenoside Ra3 | 7.19 | -4.67 | 0.01 | -6.30 | -0.87 | -2.78 | *P. ginseng* |
| Molecule0807 | Ginsenoside Rh3 | 12.09 | -0.35 | 0.65 | -1.11 | 4.69 | -4.89 | *P. ginseng* |
| Molecule0808 | Ginsenoside Rh4 | 6.76 | -0.73 | 0.63 | -1.65 | 3.49 | -4.39 | *P. ginseng* |
| Molecule0809 | Ginsenoside Rs1 | 6.27 | -3.69 | 0.46 | -5.37 | -0.02 | -3.37 | *P. ginseng* |
| Molecule0810 | Ginsenoside Rs2 | 8.19 | -4.02 | 0.79 | -5.58 | -0.34 | -3.36 | *P. ginseng* |
| Molecule0811 | Ginsenoyne A | 66.22 | 0.99 | 0.03 | 0.02 | 0.48 | 0.17 | *P. ginseng* |
| Molecule0812 | Ginsenoyne B | 39.79 | 0.79 | 0.04 | -0.10 | 0.48 | 0.17 | *P. ginseng* |
| Molecule0813 | Ginsenoyne C | 21.30 | 0.15 | 0.76 | -0.81 | 0.48 | 0.17 | *P. ginseng* |
| Molecule0814 | Ginsenoyne D | 39.23 | 1.07 | 0.06 | 0.28 | 0.66 | 0.42 | *P. ginseng* |
| Molecule0815 | Ginsenoyne E | 47.51 | 1.05 | 0.20 | 0.46 | 0.51 | -0.18 | *P. ginseng* |
| Molecule0816 | Girinimbine | 48.41 | 1.72 | 0.74 | 1.22 | 5.03 | -5.56 | *P. ginseng* |
| Molecule0817 | Gomisin B | 27.71 | 0.60 | 0.13 | 0.18 | 3.70 | -4.46 | *P. ginseng* |
| Molecule0818 | Humulene epoxide I | 33.40 | 1.58 | 0.62 | 1.72 | 5.02 | -4.45 | *P. ginseng* |
| Molecule0819 | Isocitric acid b | 32.92 | -1.46 | 0.78 | -1.71 | -0.35 | -0.56 | *P. ginseng* |
| Molecule0820 | Isocitric acid c | 9.73 | -1.67 | 0.22 | -2.22 | -0.35 | -0.56 | *P. ginseng* |
| Molecule0821 | Isocitric acid d | 8.34 | -1.58 | 0.77 | -1.98 | -0.35 | -0.56 | *P. ginseng* |
| Molecule0822 | Isocnidilide | 85.02 | 1.24 | 0.03 | 1.37 | 3.28 | -2.82 | *P. ginseng* |

Table S1: Continued

| Number | Compound | OB | Caco-2 | DL | BBB | Log*P* | Log*S* | Herbs |
| --- | --- | --- | --- | --- | --- | --- | --- | --- |
| Molecule0823 | Kaempferol-3-arabofuranoside | 2.73 | -1.05 | 0.78 | -1.93 | 0.44 | -2.25 | *P. ginseng* |
| Molecule0824 | Malkangunin | 57.61 | 0.22 | 0.56 | -0.17 | 2.72 | -3.76 | *P. ginseng* |
| Molecule0825 | Malonylginsenoside Rc | 7.84 | -4.27 | 0.08 | -5.65 | 0.00 | -3.41 | *P. ginseng* |
| Molecule0826 | Malonylginsenoside Rd | 6.29 | -3.72 | 0.10 | -4.76 | 1.22 | -3.68 | *P. ginseng* |
| Molecule0827 | Maltose-b | 1.80 | -2.70 | 0.09 | -6.56 | -3.01 | 0.23 | *P. ginseng* |
| Molecule0828 | Malvic acid | 30.99 | 1.22 | 0.77 | 0.81 | 6.00 | -4.89 | *P. ginseng* |
| Molecule0829 | Mannose-b | 43.04 | -1.82 | 0.13 | -4.46 | -2.57 | 0.64 | *P. ginseng* |
| Molecule0830 | Methyl stearate | 16.80 | 1.38 | 0.77 | 1.19 | 8.45 | -6.94 | *P. ginseng* |
| Molecule0831 | Methyl tricosanoate | 14.61 | 1.43 | 0.78 | 1.08 | 9.79 | -7.40 | *P. ginseng* |
| Molecule0832 | Mycosinol | 82.15 | 0.87 | 0.18 | -0.05 | 1.44 | 0.38 | *P. ginseng* |
| Molecule0833 | N,N-Dimethyldecanamide | 55.50 | 1.51 | 0.75 | 1.72 | 3.75 | -3.12 | *P. ginseng* |
| Molecule0834 | N-salicylidene-salicylamine | 70.06 | 0.88 | 0.10 | 0.48 | 2.90 | -3.44 | *P. ginseng* |
| Molecule0835 | Nonacosanediol-6,8 | 17.79 | 0.56 | 0.26 | 0.20 | 2.67 | -2.04 | *P. ginseng* |
| Molecule0836 | Notoginsenoside R6 | 4.77 | -3.43 | 0.19 | -4.39 | 0.17 | -2.92 | *P. ginseng* |
| Molecule0837 | Octadecyl Acetate | 16.27 | 1.39 | 0.77 | 1.06 | 8.86 | -7.13 | *P. ginseng* |
| Molecule0838 | Pancratistatin | 13.09 | -1.17 | 0.77 | -1.79 | -1.59 | -0.59 | *P. ginseng* |
| Molecule0839 | Pandamine | 16.15 | 0.11 | 0.10 | -0.34 | 3.47 | -4.39 | *P. ginseng* |
| Molecule0840 | Protopine | 57.53 | 0.84 | 0.78 | 0.21 | 1.95 | -3.24 | *P. ginseng* |
| Molecule0841 | Pseudohypericin | 19.08 | -0.32 | 0.83 | -1.96 | 3.19 | -4.54 | *P. ginseng* |
| Molecule0842 | Pyrrole-2-aldehyde | 41.58 | 1.11 | 0.09 | 1.44 | 0.52 | 0.50 | *P. ginseng* |
| Molecule0843 | Ramalic acid | 6.00 | 0.34 | 0.77 | -0.13 | 3.36 | -4.08 | *P. ginseng* |
| Molecule0844 | Se-Methyl-L-selenocysteine | 35.74 | 0.01 | 0.57 | -0.13 | -2.83 | -0.07 | *P. ginseng* |
| Molecule0845 | Stigmasterol-beta-D-glucoside | 21.32 | -0.29 | 0.10 | -1.01 | 5.30 | -5.18 | *P. ginseng* |
| Molecule0846 | Suchilactone | 57.52 | 0.82 | 0.24 | 0.28 | 3.45 | -4.89 | *P. ginseng* |
| Molecule0847 | Suffruticoside A | 5.61 | -2.04 | 0.24 | -2.84 | 0.12 | -2.77 | *P. ginseng* |
| Molecule0848 | Tauremisin | 29.22 | 0.03 | 0.08 | -0.27 | 0.75 | -2.30 | *P. ginseng* |
| Molecule0849 | Trans-9-trans-12-linoleic acid | 41.90 | 1.07 | 0.02 | 0.90 | 7.06 | -6.26 | *P. ginseng* |
| Molecule0850 | Trifolirhizin | 7.36 | -0.58 | 0.59 | -1.42 | 0.70 | -2.45 | *P. ginseng* |
| Molecule0851* | Trifolirhizin_DG | 82.10 | 0.88 | 0.64 | 0.13 | 2.19 | -2.75 | *P. ginseng* |
| Molecule0852 | Undecane, 3,6-dimethyl | 12.84 | 1.81 | 0.26 | 2.07 | 7.29 | -6.21 | *P. ginseng* |
| Molecule0853 | Vitamin B15 | 2.68 | -0.92 | 0.77 | -0.96 | 1.35 | -1.34 | *P. ginseng* |
| Molecule0854 | Vitamin B5 | 30.77 | -0.85 | 0.06 | -1.25 | -1.12 | -0.56 | *P. ginseng* |
| Molecule0855 | Alpha-cadinol | 31.69 | 1.30 | 0.22 | 1.27 | 3.52 | -3.60 | *P. ginseng, S. miltiorrhiza* |

Table S1: Continued

| Number | Compound | OB | Caco-2 | DL | BBB | Log*P* | Log*S* | Herbs |
| --- | --- | --- | --- | --- | --- | --- | --- | --- |
| Molecule0856 | Farnesene | 8.47 | 1.93 | 0.01 | 1.87 | 5.70 | -4.60 | *P. ginseng, E. herba, S. miltiorrhiza, R. chuanxiong* |
| Molecule0857 | Alpha-guriunene | 52.27 | 1.85 | 0.02 | 2.11 | 4.45 | -4.22 | *P. ginseng* |
| Molecule0858 | Alpha-guttiferin | 4.43 | 0.59 | 0.76 | 0.24 | 5.06 | -4.80 | *P. ginseng* |
| Molecule0859 | Bata-caryophyllene | 30.56 | 1.82 | 0.03 | 2.07 | 5.35 | -4.83 | *P. ginseng* |
| Molecule0860 | Beta-bisabolene | 31.84 | 1.88 | 0.03 | 2.09 | 6.01 | -4.39 | *P. ginseng, E. herba* |
| Molecule0861 | Beta-elemene | 20.20 | 1.84 | 0.32 | 2.07 | 5.40 | -4.71 | *P. ginseng* |
| Molecule0862 | Beta-humulene | 26.23 | 1.84 | 0.05 | 2.10 | 5.97 | -4.93 | *P. ginseng* |
| Molecule0863 | Beta-santalol | 35.30 | 1.28 | 0.76 | 1.25 | 4.42 | -3.91 | *P. ginseng* |
| Molecule0864 | Beta-eudesmene | 25.10 | 1.84 | 0.03 | 2.02 | 5.26 | -4.79 | *P. ginseng, R. chuanxiong* |
| Molecule0865 | Cis-11-elcosenoic acid methyl ester | 29.49 | 1.42 | 0.04 | 0.98 | 8.64 | -7.30 | *P. ginseng* |
| Molecule0866 | Cis-widdrol alpha-epoxide | 69.04 | 1.07 | 0.59 | 1.00 | 3.17 | -3.77 | *P. ginseng* |
| Molecule0867 | Dammarane | 19.73 | 1.81 | 0.57 | 1.81 | 7.95 | -7.62 | *P. ginseng* |
| Molecule0868 | Delta-elemene | 25.61 | 1.84 | 0.01 | 1.96 | 5.39 | -4.87 | *P. ginseng* |
| Molecule0869 | Delta-guaiene | 32.79 | 1.86 | 0.17 | 2.12 | 5.61 | -3.97 | *P. ginseng* |
| Molecule0870 | Epsilon-cadinene | 25.37 | 1.84 | 0.04 | 2.12 | 4.06 | -4.57 | *P. ginseng* |
| Molecule0871 | Ginsenoside Rg5 | 17.39 | -1.93 | 0.24 | -2.83 | 2.50 | -4.00 | *P. ginseng* |
| Molecule0872 | Methyl heptadecanoate | 17.41 | 1.37 | 0.77 | 1.25 | 7.97 | -6.81 | *P. ginseng* |
| Molecule0873 | Methyl myristate | 14.61 | 1.34 | 0.58 | 1.31 | 6.38 | -5.97 | *P. ginseng* |
| Molecule0874 | Methyl pentadecanoate | 18.82 | 1.37 | 0.20 | 1.28 | 6.86 | -6.28 | *P. ginseng, R. chuanxiong* |
| Molecule0875 | Oleanane | 6.69 | 1.86 | 0.22 | 1.98 | 7.24 | -7.37 | *P. ginseng* |
| Molecule0876 | P-glucosyloxymandelonitrile | 9.14 | -0.93 | 0.04 | -1.61 | -0.89 | -1.52 | *P. ginseng* |
| Molecule0877 | Palmitelaidic acid methyl ester | 34.61 | 1.40 | 0.14 | 1.16 | 6.84 | -6.44 | *P. ginseng* |
| Molecule0878 | (2e,6e)-3,7,11-trimethyl-2,6,10-dodecane triene ol | 41.14 | 1.30 | 0.06 | 1.15 | 4.84 | -3.58 | *P. notoginseng* |
| Molecule0879 | (r)-(1-methoxyethyl)benzene | 47.22 | 1.60 | 0.02 | 1.89 | 2.32 | -2.57 | *P. notoginseng* |
| Molecule0880 | 1,3-cyclooctadiene | 42.82 | 1.82 | 0.01 | 2.21 | 3.56 | -3.35 | *P. notoginseng* |
| Molecule0881 | 1,4,5-trimethyl-5,6-dialin | 52.69 | 1.90 | 0.06 | 2.12 | 4.96 | -4.91 | *P. notoginseng* |
| Molecule0882 | 1,4,6-trimethyl-1,2,3,4-tetralin | 50.13 | 1.88 | 0.06 | 2.12 | 5.16 | -5.30 | *P. notoginseng* |
| Molecule0883 | 1,6-dimethyl-4-isopropylnaphthalene | 13.03 | 1.95 | 0.08 | 1.92 | 5.70 | -6.06 | *P. notoginseng* |
| Molecule0884 | 1-ethyl-2-methyl cyclopropane | 44.67 | 1.79 | 0.01 | 2.21 | 2.93 | -2.93 | *P. notoginseng* |

Table S1: Continued

| Number | Compound | OB | Caco-2 | DL | BBB | Log*P* | Log*S* | Herbs |
| --- | --- | --- | --- | --- | --- | --- | --- | --- |
| Molecule0885 | 1-methyl-5-isopropenyl cyclohexene | 53.20 | 1.84 | 0.02 | 2.03 | 4.50 | -2.72 | *P. notoginseng* |
| Molecule0886 | 10-methyl nonadecane | 10.28 | 1.83 | 0.12 | 1.78 | 9.75 | -7.60 | *P. notoginseng* |
| Molecule0887 | 14-methyl pentadecanoate | 22.65 | 1.34 | 0.11 | 1.14 | 7.03 | -6.57 | *P. notoginseng* |
| Molecule0888 | 2,3-dichloroaniline | 49.51 | 1.18 | 0.02 | 1.08 | 2.73 | -2.43 | *P. notoginseng* |
| Molecule0889 | 2,6-dimethyl-cyclohexanol | 76.28 | 1.21 | 0.02 | 1.40 | 2.09 | -1.60 | *P. notoginseng* |
| Molecule0890 | 2,6-ditertbutyl paracresol | 45.08 | 1.75 | 0.07 | 1.81 | 5.25 | -4.16 | *P. notoginseng* |
| Molecule0891 | 2-ethanoyl-1h-pyrrole | 63.92 | 1.13 | 0.01 | 1.32 | 0.82 | 0.40 | *P. notoginseng* |
| Molecule0892 | 2-hydroxycinnamic acid | 66.00 | 0.44 | 0.04 | 0.25 | 1.90 | -2.15 | *P. notoginseng* |
| Molecule0893 | 2-octanone | 8.35 | 1.33 | 0.01 | 1.60 | 2.54 | -2.25 | *P. notoginseng* |
| Molecule0894 | 3',5'-dimethoxy acetophenone | 27.95 | 0.95 | 0.04 | 0.60 | 1.67 | -2.07 | *P. notoginseng* |
| Molecule0895 | 3,4-dichloroaniline | 9.92 | 1.88 | 0.02 | 2.22 | 3.04 | -2.66 | *P. notoginseng* |
| Molecule0896 | 3-ethyl-2,4-pentylene alcohol | 51.98 | 1.06 | 0.01 | 1.13 | 2.01 | -1.09 | *P. notoginseng* |
| Molecule0897 | 4-methoxyphenol | 48.98 | 1.27 | 0.02 | 1.33 | 1.31 | -0.54 | *P. notoginseng* |
| Molecule0898 | 5-methyl furfural | 49.49 | 1.07 | 0.01 | 1.48 | 0.69 | -0.87 | *P. notoginseng, G. biloba* |
| Molecule0899 | 5-octadecyne | 15.90 | 1.93 | 0.09 | 2.09 | 8.09 | -5.91 | *P. notoginseng* |
| Molecule0900 | 6-hydrogenation-(5e,9e)-6,10,14-trimethylpentadeca-5,9,13-trien-2-one | 23.30 | 1.43 | 0.10 | 1.30 | 7.48 | -6.83 | *P. notoginseng* |
| Molecule0901 | 9,12-octadecadienoic acid methyl ester | 46.93 | 1.43 | 0.17 | 1.10 | 6.95 | -6.76 | *P. notoginseng* |
| Molecule0902 | Alloaromadedrene | 60.07 | 1.84 | 0.10 | 2.15 | 3.70 | -4.94 | *P. notoginseng* |
| Molecule0903 | Benzenemethanol | 60.82 | 1.25 | 0.03 | 1.45 | 2.05 | -1.49 | *P. notoginseng* |
| Molecule0904 | Dencichine | 71.70 | -1.40 | 0.04 | -1.75 | -3.39 | -1.09 | *P. notoginseng* |
| Molecule0905 | Di(2-ethylhexyl)phthalate | 48.59 | 1.11 | 0.39 | 0.71 | 7.02 | -6.58 | *P. notoginseng* |
| Molecule0906 | Ginsenoside F1 | 4.05 | -1.20 | 0.60 | -2.12 | 2.68 | -3.94 | *P. notoginseng, P. ginseng* |
| Molecule0907 | Ginsenoside F2 | 36.43 | -1.80 | 0.25 | -3.03 | 2.24 | -3.95 | *P. notoginseng* |
| Molecule0908 | Ginsenoside Rb1 | 6.29 | -3.72 | 0.04 | -4.95 | -0.24 | -3.02 | *P. notoginseng, P. ginseng* |
| Molecule0909 | Ginsenoside Rb2 | 17.74 | -3.46 | 0.04 | -4.89 | -0.02 | -3.14 | *P. notoginseng, P. ginseng* |
| Molecule0910 | Ginsenoside Rb3 | 5.99 | -3.97 | 0.04 | -5.24 | -0.02 | -3.14 | *P. notoginseng, P. ginseng* |
| Molecule0911 | Ginsenoside Rc | 8.12 | -3.85 | 0.04 | -5.22 | -0.17 | -3.14 | *P. notoginseng, P. ginseng* |
| Molecule0912 | Ginsenoside Rd | 5.42 | -2.88 | 0.09 | -4.20 | 0.73 | -3.39 | *P. ginseng, P. notoginseng* |

Table S1: Continued

| Number | Compound | OB | Caco-2 | DL | BBB | Log*P* | Log*S* | Herbs |
| --- | --- | --- | --- | --- | --- | --- | --- | --- |
| Molecule0913 | Ginsenoside Re | 5.43 | -2.96 | 0.12 | -4.24 | 1.13 | -3.28 | *P. notoginseng, P. ginseng* |
| Molecule0914 | Ginsenoside Rf | 7.44 | -2.12 | 0.24 | -3.13 | 1.88 | -3.66 | *P. notoginseng, P. ginseng* |
| Molecule0915 | Ginsenoside Rg1 | 17.74 | -2.26 | 0.28 | -3.42 | 1.00 | -3.42 | *P. notoginseng, P. ginseng* |
| Molecule0916 | Ginsenoside rg2 | 10.09 | -1.83 | 0.26 | -2.65 | 1.88 | -3.66 | *P. notoginseng* |
| Molecule0917 | Ginsenoside Rg3 | 5.94 | -4.05 | 0.04 | -5.56 | 2.27 | -3.90 | *P. notoginseng, P. ginseng* |
| Molecule0918 | Ginsenoside Rh1 | 5.00 | -1.13 | 0.57 | -2.11 | 2.68 | -3.93 | *P. notoginseng, P. ginseng* |
| Molecule0919 | Ginsenoside Rh2 | 6.54 | -0.83 | 0.56 | -1.73 | 3.77 | -4.71 | *P. notoginseng, P. ginseng* |
| Molecule0920 | Ginsenoside R0 | 1.92 | -2.97 | 0.05 | -4.10 | 2.03 | -3.72 | *P. notoginseng, P. ginseng* |
| Molecule0921 | N-(3,5-dichlorophenyl)-1,2-dimethyl-1,2-cyclopropane dicarboximide | 50.66 | 0.90 | 0.15 | 0.55 | 2.83 | -3.49 | *P. notoginseng* |
| Molecule0922 | Notoginsenoside r1 | 8.59 | -2.98 | 0.13 | -4.15 | 0.13 | -2.95 | *P. notoginseng* |
| Molecule0923 | Notoginsenoside r2 | 10.32 | -1.92 | 0.28 | -2.80 | 1.44 | -3.53 | *P. notoginseng, P. ginseng* |
| Molecule0924 | Notoginsenoside r3 | 6.24 | -4.27 | 0.04 | -5.64 | -0.24 | -3.02 | *P. notoginseng* |
| Molecule0925 | Notoginsenoside r4 | 7.34 | -4.51 | 0.02 | -5.95 | -0.78 | -2.81 | *P. notoginseng* |
| Molecule0926 | Pentadecane | 13.98 | 1.81 | 0.05 | 1.87 | 8.17 | -6.77 | *P. notoginseng, P. ginseng, R. chuanxiong* |
| Molecule0927 | Acetophenone | 53.78 | 1.36 | 0.02 | 1.61 | 1.65 | -1.95 | *P. notoginseng, E. herba* |
| Molecule0928 | Butyl cyclobutane | 52.59 | 1.79 | 0.01 | 2.16 | 4.57 | -4.40 | *P. notoginseng* |
| Molecule0929 | Cadinene | 23.29 | 1.77 | 0.08 | 2.03 | 5.70 | -6.29 | *P. notoginseng* |
| Molecule0930 | Ditertbutyl phthalate | 67.02 | 0.86 | 0.13 | 0.77 | 4.32 | -4.57 | *P. notoginseng, P. ginseng* |
| Molecule0931 | Furan butanone | 11.01 | -0.87 | 0.19 | -1.37 | -0.67 | -1.47 | *P. notoginseng* |
| Molecule0932 | Germacrene | 20.80 | 1.85 | 0.06 | 2.15 | 6.65 | -6.36 | *P. notoginseng, R. chuanxiong* |
| Molecule0933 | Glucuronic acid | 3.95 | -1.92 | 0.04 | -4.78 | -2.32 | -0.29 | *P. notoginseng* |
| Molecule0934 | Glycyrrhizin | 35.00 | 0.51 | 0.18 | -0.22 | 2.79 | -3.28 | *P. notoginseng* |

Table S1: Continued

| Number | Compound | OB | Caco-2 | DL | BBB | Log*P* | Log*S* | Herbs |
| --- | --- | --- | --- | --- | --- | --- | --- | --- |
| Molecule0935 | Gypenosideix | 8.82 | -2.70 | 0.11 | -4.15 | 1.12 | -3.69 | *P. notoginseng* |
| Molecule0936* | Gypenosideix_DG | 12.84 | 0.40 | 0.77 | -0.41 | 5.36 | -5.12 | *P. notoginseng* |
| Molecule0937 | Gypenosidexv II | 4.86 | -3.07 | 0.10 | -4.51 | 0.79 | -3.50 | *P. notoginseng* |
| Molecule0938 | Hexanal | 19.59 | 1.23 | 0.01 | 1.47 | 2.37 | -1.35 | *P. notoginseng, E. herba, R. chuanxiong* |
| Molecule0939 | Hexenal | 54.22 | 1.26 | 0.01 | 1.52 | 1.80 | -1.49 | *P. notoginseng* |
| Molecule0940 | Isopulegone | 62.06 | 0.66 | 0.03 | 0.81 | 1.50 | -1.47 | *P. notoginseng* |
| Molecule0941 | Isopropyl benzene | 51.93 | 1.85 | 0.02 | 2.10 | 3.67 | -3.51 | *P. notoginseng* |
| Molecule0942 | Linoleny alcohol | 47.79 | 1.36 | 0.12 | 1.05 | 6.61 | -6.43 | *P. notoginseng* |
| Molecule0943 | Lutein | 17.88 | 1.09 | 0.55 | -0.82 | 8.29 | -5.89 | *P. notoginseng* |
| Molecule0944 | Palustrol | 82.08 | 1.44 | 0.12 | 1.65 | 3.15 | -4.72 | *P. notoginseng* |
| Molecule0945 | Panaxatriol | 15.44 | 0.52 | 0.79 | -0.07 | 4.59 | -5.35 | *P. notoginseng, P. ginseng* |
| Molecule0946 | Panaxydol | 25.83 | 1.07 | 0.13 | 0.11 | 4.68 | -4.60 | *P. notoginseng* |
| Molecule0947 | Panaxynol | 16.26 | 1.44 | 0.10 | 0.66 | 5.93 | -5.25 | *P. notoginseng, P. ginseng* |
| Molecule0948 | Panaxytriol | 33.76 | 0.05 | 0.13 | -0.98 | 0.48 | 0.17 | *P. notoginseng, P. ginseng* |
| Molecule0949 | Panaxadiol | 17.14 | 0.88 | 0.80 | 0.38 | 5.49 | -6.28 | *P. notoginseng, P. ginseng* |
| Molecule0950 | Parachlorophenol | 66.56 | 1.57 | 0.01 | 1.75 | 2.37 | -0.96 | *P. notoginseng* |
| Molecule0951 | Protopanaxatriol | 12.65 | 0.05 | 0.78 | -0.65 | 4.21 | -4.48 | *P. notoginseng* |
| Molecule0952 | Protopanoxadiol | 29.61 | 0.42 | 0.78 | -0.29 | 4.74 | -5.00 | *P. notoginseng* |
| Molecule0953 | Sanchinan-a | 7.57 | -8.82 | 0.03 | -14.19 | -2.25 | -0.62 | *P. notoginseng* |
| Molecule0954 | Valeraldehyde | 63.74 | 1.19 | 0.00 | 1.54 | 1.41 | -0.76 | *P. notoginseng* |
| Molecule0955 | α-cyperene | 55.07 | 1.81 | 0.11 | 2.08 | 5.51 | -4.26 | *P. notoginseng* |
| Molecule0956 | α-copaene | 42.81 | 1.83 | 0.08 | 2.05 | 5.82 | -5.47 | *P. notoginseng* |
| Molecule0957 | α-guaiene | 25.94 | 1.84 | 0.07 | 2.11 | 5.80 | -3.94 | *P. notoginseng* |
| Molecule0958 | α-gurjunene | 57.26 | 1.85 | 0.10 | 2.10 | 4.45 | -4.22 | *P. notoginseng, E. herba* |
| Molecule0959 | α-pinene | 51.20 | 1.82 | 0.05 | 2.12 | 3.66 | -2.94 | *P. notoginseng, E. herba, S. miltiorrhiza, R. chuanxiong* |
| Molecule0960 | β-elemene | 26.43 | 1.86 | 0.06 | 2.12 | 5.40 | -4.71 | *P. notoginseng* |

Table S1: Continued

| Number | Compound | OB | Caco-2 | DL | BBB | Log*P* | Log*S* | Herbs |
| --- | --- | --- | --- | --- | --- | --- | --- | --- |
| Molecule0961 | β-guaiene | 22.97 | 1.86 | 0.07 | 2.14 | 5.52 | -3.44 | *P. notoginseng* |
| Molecule0962 | β-phenethyl alcohol | 47.63 | 1.13 | 0.02 | 1.18 | 1.51 | -1.03 | *P. notoginseng* |
| Molecule0963 | β-pinene | 49.84 | 1.80 | 0.05 | 2.16 | 3.94 | -3.33 | *P. notoginseng* |
| Molecule0964 | γ-caprolactone | 75.69 | 1.12 | 0.01 | 1.40 | 0.77 | -0.71 | *P. notoginseng* |
| Molecule0965 | δ-guaiene | 18.22 | 1.83 | 0.07 | 2.12 | 5.61 | -3.97 | *P. notoginseng* |
| Molecule0966 | 2-hydroxybenzyl alcohol | 57.32 | 0.69 | 0.02 | 0.53 | 0.40 | -0.26 | *G. elata* |
| Molecule0967 | 3-hydroxybenzyl alcohol | 58.54 | 0.65 | 0.02 | 0.47 | 0.47 | -0.30 | *G. elata* |
| Molecule0968 | 4-ethoxymethylphenyl-4'-hydroxybenzyl ether | 3.20 | 0.95 | 0.66 | 0.08 | 6.21 | -6.33 | *G. elata* |
| Molecule0969 | P-hydroxybenzyl ether ether | 3.01 | 0.81 | 0.82 | -0.23 | 5.24 | -5.80 | *G. elata* |
| Molecule0970 | 4-hydroxybenzaldehyde | 18.85 | 0.80 | 0.02 | 0.65 | 1.27 | -1.11 | *G. elata* |
| Molecule0971 | 3-O-(4-hydroxybenzyl)-β-sitosterol | 19.65 | 1.42 | 0.76 | 0.77 | 8.52 | -7.91 | *G. elata* |
| Molecule0972 | Daucosterol | 20.63 | 0.03 | 0.63 | -0.69 | 5.71 | -5.31 | *G. elata, P. notoginseng, P. ginseng, S. miltiorrhiza, S. baicalensis, U. rhynchophylla, E. breviscapu, R. chuanxiong* |
| Molecule0973 | Bis-(4-hydroxybenzyl) hydroxylamide | 36.68 | 0.49 | 0.12 | 0.08 | 2.13 | -3.03 | *G. elata* |
| Molecule0974 | 4,4'-dihydroxydibenzyl sulfoxide | 3.64 | 0.22 | 0.12 | -0.20 | 2.05 | -2.50 | *G. elata* |
| Molecule0975 | Vanillyl alcohol | 37.70 | 0.56 | 0.03 | 0.24 | 0.50 | -0.72 | *G. elata* |
| Molecule0976 | 4-(4'-hydroxybenzyloxy)-benzyl methyl ether | 7.83 | 1.08 | 0.12 | 0.45 | 2.91 | -3.87 | *G. elata* |
| Molecule0977 | 4-hydroxybenzyl methyl ether | 26.85 | 1.06 | 0.02 | 0.99 | 1.23 | -1.05 | *G. elata* |
| Molecule0978 | 4,4'-dihydroxydibenzyl ether | 21.29 | 0.85 | 0.11 | 0.31 | 2.61 | -3.54 | *G. elata* |
| Molecule0979 | 4,4'-dihydroxydiphenyl methane | 42.87 | 1.17 | 0.08 | 0.69 | 2.61 | -3.10 | *G. elata* |
| Molecule0980 | 4-hydroxybenzyl alcohol | 55.21 | 0.60 | 0.02 | 0.34 | 0.53 | -0.32 | *G. elata* |
| Molecule0981 | Parishin | 3.01 | -4.12 | 0.14 | -5.00 | -0.62 | -3.12 | *G. elata* |
| Molecule0982* | Parishin_DG | 0.89 | -0.31 | 0.63 | -0.85 | 3.03 | -4.52 | *G. elata* |
| Molecule0983 | 4-[4’-(4”-hydroxybenzyloxy)benzyloxy]benzyl alcohol | 6.50 | 0.81 | 0.39 | -0.33 | 3.91 | -4.46 | *G. elata* |
| Molecule0984 | 2,2'-methylene bis(6-tert-butyl-4-methyl-phenol) | 17.51 | 1.53 | 0.26 | 1.22 | 5.89 | -5.55 | *G. elata* |
| Molecule0985 | Bis-(4-hydroxybenzy) sulfide | 83.57 | 1.08 | 0.11 | 0.63 | 2.98 | -3.71 | *G. elata* |

Table S1: Continued

| Number | Compound | OB | Caco-2 | DL | BBB | Log*P* | Log*S* | Herbs |
| --- | --- | --- | --- | --- | --- | --- | --- | --- |
| Molecule0986 | Dotriacontanoic acid | 12.80 | 1.35 | 0.49 | 0.96 | 10.49 | -7.59 | *G. elata* |
| Molecule0987 | 2,4-bis(4-hydroxybenzyl)phenol | 30.49 | 1.00 | 0.28 | 0.08 | 3.75 | -4.45 | *G. elata* |
| Molecule0988 | 4-(4'-hydroxybenzyloxy)benzyl alcohol | 18.15 | 0.69 | 0.11 | -0.07 | 2.32 | -3.50 | *G. elata* |
| Molecule0989 | 4'-hydroxybenzyl-4-hydroxy-3-(4''-hydroxybenzyl)benzy ether | 1.81 | 0.78 | 0.37 | -0.13 | 3.55 | -4.49 | *G. elata* |
| Molecule0990 | Protocaterchualdehyde | 58.41 | 0.41 | 0.03 | 0.18 | 0.89 | -0.99 | *G. elata, S. miltiorrhiza, E. breviscapu* |
| Molecule0991 | Citric acid | 67.56 | -1.28 | 0.05 | -1.85 | -1.33 | -0.26 | *G. elata* |
| Molecule0992 | 4-[4’-(4”-hydroxybenzyloxy)benzyloxy]benzyl methyl ether | 7.14 | 1.13 | 0.44 | 0.10 | 4.31 | -5.35 | *G. elata* |
| Molecule0993 | 2-methyl citrate | 24.98 | -0.87 | 0.05 | -1.07 | -1.30 | 0.01 | *G. elata* |
| Molecule0994 | 1,5-dimethyl citrat | 75.18 | -0.44 | 0.06 | -0.95 | -0.68 | -0.15 | *G. elata* |
| Molecule0995 | Dimethyl phthalate | 33.51 | 0.83 | 0.06 | 0.94 | 1.96 | -2.25 | *G. elata* |
| Molecule0996 | 4-hydroxy- 3-(4'-hydroxybenzyl)benzyl alcohol | 41.88 | 0.54 | 0.11 | -0.17 | 1.83 | -3.32 | *G. elata* |
| Molecule0997 | Gastrodamine | 52.04 | 0.49 | 0.12 | -0.01 | 2.13 | -3.03 | *G. elata* |
| Molecule0998 | Docosanoic acid oxiranylmethyl ester | 19.85 | 1.18 | 0.42 | 0.63 | 9.37 | -7.27 | *G. elata* |
| Molecule0999 | Succinic acid | 24.93 | -0.47 | 0.01 | -0.79 | -0.53 | 0.25 | *G. elata, S. miltiorrhiza* |
| Molecule1000 | Adenine | 38.91 | 0.12 | 0.03 | -0.01 | -0.38 | -1.07 | *G. elata, R. chuanxiong* |
| Molecule1001 | 5,5'-oxybis(methylene)difuran-2-carbaldehyde | 29.31 | 0.21 | 0.11 | -0.12 | 1.38 | -3.21 | *G. elata* |
| Molecule1002 | N-(4-hydroxybenzyl)-adenosine | 24.70 | -0.75 | 0.58 | -1.57 | -0.13 | -2.09 | *G. elata* |
| Molecule1003 | 4-methylphenyl-1-O-β-D-glucopyranoside | 21.03 | -0.34 | 0.14 | -0.66 | 0.22 | -1.12 | *G. elata* |
| Molecule1004 | P-xylene | 51.99 | 1.53 | 0.01 | 1.81 | 1.95 | -0.67 | *G. elata* |
| Molecule1005 | 3,5-dimethoxybenzoie acid-4-O-β-D-glucopyranoside | 30.47 | -1.17 | 0.30 | -1.74 | -0.86 | -1.60 | *G. elata* |
| Molecule1006 | 3,5-dimethoxybenzoie acid | 17.47 | 0.48 | 0.06 | 0.14 | 1.43 | -2.08 | *G. elata* |
| Molecule1007 | Adenosine | 18.96 | -1.64 | 0.18 | -2.36 | -1.21 | -1.28 | *G. elata, P. ginseng, R. chuanxiong* |
| Molecule1008 | L-pyroglutamic acid | 109.58 | -0.18 | 0.02 | -0.33 | -1.01 | 0.07 | *G. elata* |

Table S1: Continued

| Number | Compound | OB | Caco-2 | DL | BBB | Log*P* | Log*S* | Herbs |
| --- | --- | --- | --- | --- | --- | --- | --- | --- |
| Molecule1009 | Parishin C | 3.08 | -3.13 | 0.34 | -3.90 | -0.85 | -2.97 | *G. elata* |
| Molecule1010* | Parishin C_DG | 6.19 | -0.37 | 0.45 | -0.89 | 1.91 | -3.83 | *G. elata* |
| Molecule1011 | 4-ethoxymethylphenyl-1-O-β-D-glucopyranosid | 10.95 | -0.31 | 0.19 | -0.62 | -0.01 | -1.22 | *G. elata* |
| Molecule1012 | 4-methoxymethylphenol | 26.78 | 1.19 | 0.03 | 1.17 | 1.85 | -1.52 | *G. elata* |
| Molecule1013 | Vanillin | 69.24 | 0.67 | 0.03 | 0.56 | 1.31 | -1.48 | *G. elata, R. chuanxiong* |
| Molecule1014 | GAFT AmDz-9 | 30.26 | 0.22 | 0.11 | -0.10 | 1.38 | -3.21 | *G. elata* |
| Molecule1015 | GAFT AmDz-20 | 38.92 | 0.13 | 0.66 | -0.40 | 4.71 | -6.62 | *G. elata* |
| Molecule1016 | Parishin B | 3.12 | -2.91 | 0.35 | -3.70 | -0.90 | -2.96 | *G. elata* |
| Molecule1017* | Parishin B_DG | 6.24 | -0.55 | 0.43 | -1.05 | 1.77 | -3.86 | *G. elata* |
| Molecule1018 | Cymbinodin A | 50.26 | 0.71 | 0.21 | 0.28 | 3.04 | -3.70 | *G. elata* |
| Molecule1019 | 5-hydroxymethyl-2-furancarboxaldehyde | 49.13 | 0.15 | 0.02 | -0.17 | -0.17 | -0.61 | *G. elata* |
| Molecule1020 | S-(4-hydroxybenzyl)glutathione | 11.18 | -2.96 | 0.43 | -3.88 | -3.13 | -3.68 | *G. elata* |
| Molecule1021 | 4,4'-dihydroxybenzyl sulfone | 25.48 | -0.22 | 0.17 | -0.48 | 0.35 | -2.18 | *G. elata* |
| Molecule1022 | Benzoic acid | 25.20 | 0.86 | 0.02 | 0.99 | 1.72 | -1.24 | *G. elata, E. herba, S. baicalensis, U. rhynchophylla* |
| Molecule1023 | Gastrol A | 3.08 | 0.93 | 0.39 | -0.04 | 3.89 | -4.51 | *G. elata* |
| Molecule1024 | Gastrodin | 7.56 | -1.21 | 0.17 | -1.86 | -1.11 | -1.10 | *G. elata* |
| Molecule1025* | Gastrodin_DG | 55.19 | 0.61 | 0.02 | 0.29 | 0.53 | -0.32 | *G. elata* |
| Molecule1026 | Gastrol | 1.81 | 0.74 | 0.37 | -0.02 | 3.55 | -4.49 | *G. elata* |
| Molecule1027 | 2, 4-bis(4-hydroxybenzyl)phenol | 30.49 | 1.00 | 0.28 | 0.08 | 3.75 | -4.45 | *G. elata* |
| Molecule1028 | Gastrol B | 6.01 | -1.59 | 0.11 | -2.02 | -2.82 | -0.36 | *G. elata* |
| Molecule1029 | Bis-(4-hydroxybenzyl)ether-mono-β-D-glucopyranoside / gastrodioside | 8.76 | -0.79 | 0.52 | -1.43 | 0.26 | -2.69 | *G. elata* |
| Molecule1030* | Bis-(4-hydroxybenzyl)ether-mono-β-D-glucopyranoside_DG / gastrodioside | 3.34 | 0.85 | 0.11 | 0.32 | 2.61 | -3.54 | *G. elata* |
| Molecule1031 | Bis(4-hydroxybenzyl)ether | 3.34 | 0.86 | 0.11 | 0.31 | 2.61 | -3.54 | *G. elata* |
| Molecule1032 | P-ethoxymethylphenol | 34.11 | 1.20 | 0.03 | 1.24 | 1.85 | -1.52 | *G. elata* |
| Molecule1033 | (-)-camphor | 67.30 | 1.29 | 0.05 | 1.73 | 2.85 | -2.24 | *G. biloba* |
| Molecule1034 | (+) - longifolene | 15.11 | 1.79 | 0.11 | 2.12 | 4.65 | -5.21 | *G. biloba* |

Table S1: Continued

| Number | Compound | OB | Caco-2 | DL | BBB | Log*P* | Log*S* | Herbs |
| --- | --- | --- | --- | --- | --- | --- | --- | --- |
| Molecule1035 | α-cedrene | 52.59 | 1.81 | 0.10 | 2.10 | 5.18 | -5.11 | *G. biloba, P. notoginseng* |
| Molecule1036 | (E) -β- farnesene | 28.19 | 1.89 | 0.05 | 2.07 | 5.70 | -4.78 | *G. biloba* |
| Molecule1037 | (E)-2-Heptenal | 38.47 | 1.32 | 0.01 | 1.68 | 2.30 | -1.99 | *G. biloba* |
| Molecule1038 | [Epicatechin-(48)]5-epicatechin | 4.01 | -4.49 | 0.00 | -7.15 | 4.51 | -3.33 | *G. biloba* |
| Molecule1039 | 1-butanol | 87.92 | 0.95 | 0.00 | 1.09 | 0.84 | 0.33 | *G. biloba* |
| Molecule1040 | Hexanol | 20.59 | 1.08 | 0.01 | 1.16 | 2.03 | -1.09 | *G. biloba, E. herba* |
| Molecule1041 | 1- pentadecene | 5.35 | 1.82 | 0.05 | 2.02 | 7.70 | -6.49 | *G. biloba* |
| Molecule1042 | 1,2-dihydro-1,5,8-trimethylnaphthalene | 48.59 | 1.91 | 0.06 | 2.07 | 4.96 | -4.91 | *G. biloba* |
| Molecule1043 | 1,2-diphenylethylenediamine | 74.37 | 0.82 | 0.08 | 0.39 | 1.22 | -3.22 | *G. biloba* |
| Molecule1044 | 1-methylnaphthalene | 38.95 | 1.84 | 0.04 | 1.98 | 3.84 | -3.94 | *G. biloba* |
| Molecule1045 | 2- furanmethanol | 49.37 | 0.79 | 0.01 | 1.00 | 0.25 | -0.46 | *G. biloba* |
| Molecule1046 | 2- nonenal | 20.55 | 1.36 | 0.02 | 1.56 | 3.56 | -3.18 | *G. biloba* |
| Molecule1047 | 2- octenal | 39.64 | 1.33 | 0.01 | 1.47 | 4.02 | -3.41 | *G. biloba* |
| Molecule1048 | 2,3-dihydro-4-methyl-furan | 97.97 | 1.45 | 0.01 | 1.89 | 1.28 | 0.00 | *G. biloba* |
| Molecule1049 | 2,4-heptadienal | 25.09 | 1.33 | 0.01 | 1.62 | 2.13 | -2.06 | *G. biloba, P. notoginseng* |
| Molecule1050 | 2,6-dimethyl-pyridine | 41.33 | 1.58 | 0.01 | 1.89 | 1.60 | -0.08 | *G. biloba* |
| Molecule1051 | 2,2,3,3-tetramcthylbutane | 42.95 | 1.78 | 0.02 | 2.19 | 4.51 | -3.82 | *G. biloba* |
| Molecule1052 | 2,3-dihydro-7-hydroxy-3-methyl-1H-inden-1-one | 60.63 | 1.06 | 0.05 | 1.18 | 2.07 | -1.75 | *G. biloba* |
| Molecule1053 | 2,3-dihydrosciadopitysin | 0.89 | 0.19 | 0.54 | -1.38 | 4.49 | -5.17 | *G. biloba* |
| Molecule1054 | 2,3-dimethyl octane | 18.81 | 1.82 | 0.01 | 2.16 | 5.25 | -5.16 | *G. biloba* |
| Molecule1055 | 2,3-dimethylhexane | 46.24 | 1.77 | 0.01 | 2.15 | 4.59 | -4.21 | *G. biloba* |
| Molecule1056 | 2,4,6,8-tetramethyl-1-undecene | 15.23 | 1.84 | 0.04 | 2.01 | 6.73 | -5.90 | *G. biloba* |
| Molecule1057 | 2,5-octadecadiynoic acid, methyl ester | 6.88 | 1.46 | 0.17 | 0.95 | 7.05 | -5.27 | *G. biloba* |
| Molecule1058 | 2-butyl-1,1,3-trimethyl cyclohexane | 26.74 | 1.80 | 0.04 | 2.16 | 6.05 | -5.97 | *G. biloba* |
| Molecule1059 | 2-isopropyl-2,5-dimethyl-cyclohexanone | 65.44 | 1.35 | 0.04 | 1.61 | 3.28 | -3.21 | *G. biloba* |
| Molecule1060 | 2-methoxy-4-vinylphenol | 17.91 | 1.43 | 0.03 | 1.58 | 1.84 | -1.31 | *G. biloba, R. chuanxiong* |
| Molecule1061 | 2-methyl-1-penten-3-ol | 79.90 | 1.11 | 0.01 | 1.41 | 1.47 | -0.29 | *G. biloba* |
| Molecule1062 | 2-methyl-1-phenyl-2-propanol | 45.14 | 1.27 | 0.03 | 1.39 | 2.44 | -2.06 | *G. biloba* |
| Molecule1063 | 2-methyleicosane | 10.18 | 1.86 | 0.15 | 1.99 | 9.99 | -7.74 | *G. biloba* |
| Molecule1064 | 2-nonacosanone | 56.97 | 1.27 | 0.00 | 1.63 | 0.87 | -0.47 | *G. biloba* |

Table S1: Continued

| Number | Compound | OB | Caco-2 | DL | BBB | Log*P* | Log*S* | Herbs |
| --- | --- | --- | --- | --- | --- | --- | --- | --- |
| Molecule1065 | 2-pentylfuran | 51.39 | 1.70 | 0.02 | 1.99 | 4.06 | -3.26 | *G. biloba, E. herba, R. chuanxiong* |
| Molecule1066 | 2-phenyldodecane | 4.93 | 1.91 | 0.10 | 2.16 | 8.23 | -7.16 | *G. biloba* |
| Molecule1067 | 2-phenyltetradecane | 31.84 | 1.95 | 0.14 | 2.10 | 8.88 | -7.49 | *G. biloba* |
| Molecule1068 | 3-(2-pentenyl)-1,2,4-cyclopentanetrione | 94.05 | 0.08 | 0.05 | -0.17 | 1.89 | -2.05 | *G. biloba* |
| Molecule1069 | 3, 5- dimethyl- phenol | 43.49 | 1.58 | 0.02 | 1.75 | 2.38 | -1.33 | *G. biloba* |
| Molecule1070 | 3,4,4a,5,6,7-hexahydro-1,1,4a-trimethyl-2(1H)-napthalenone | 59.52 | 1.38 | 0.08 | 1.62 | 4.03 | -3.10 | *G. biloba* |
| Molecule1071 | 3,4-dimethyl cyclohexanol | 43.57 | 1.26 | 0.02 | 1.58 | 2.16 | -1.55 | *G. biloba* |
| Molecule1072 | 3,4-dimethylfuran-2,5-dione | 94.19 | 0.54 | 0.02 | 0.40 | 0.62 | -0.37 | *G. biloba* |
| Molecule1073 | 3-ethyl-4-methyl-pyrrole-2,5-dione | 101.72 | 0.50 | 0.03 | 0.43 | 0.41 | -1.08 | *G. biloba* |
| Molecule1074 | 3-ethylhexane | 50.72 | 1.77 | 0.01 | 2.04 | 4.83 | -4.39 | *G. biloba* |
| Molecule1075 | 3-heptylacrolein | 63.51 | 1.40 | 0.02 | 1.61 | 4.32 | -3.87 | *G. biloba* |
| Molecule1076 | 3-hexen-1-ol | 57.76 | 1.19 | 0.01 | 1.40 | 1.69 | -0.79 | *G. biloba* |
| Molecule1077 | 3-hydroxy-2-butanone | 40.78 | 0.37 | 0.01 | 0.43 | -0.66 | 0.73 | *G. biloba* |
| Molecule1078 | 3-methyl-2-cyclopenten-1-one | 93.70 | 1.20 | 0.01 | 1.58 | 0.48 | -0.42 | *G. biloba* |
| Molecule1079 | 3-methylacetophenone | 40.47 | 1.39 | 0.02 | 1.66 | 2.08 | -2.51 | *G. biloba, E. herba* |
| Molecule1080 | 3-methylbenzyl alcohol | 52.47 | 1.13 | 0.02 | 1.13 | 1.53 | -1.08 | *G. biloba* |
| Molecule1081 | 3'-methylmyricetin | 5.64 | -0.08 | 0.34 | -0.69 | 1.13 | -2.60 | *G. biloba* |
| Molecule1082 | 3-n-pentadecylphenol | 13.93 | 1.68 | 0.21 | 1.62 | 8.94 | -6.93 | *G. biloba* |
| Molecule1083 | 3-penten-2-one | 50.20 | 1.27 | 0.00 | 1.68 | 1.48 | -0.42 | *G. biloba* |
| Molecule1084 | 3-picoline | 68.71 | 1.52 | 0.01 | 1.83 | 1.11 | 0.33 | *G. biloba* |
| Molecule1085 | 4,4'-(ethene-1,2-diyl)bis(2-methoxyphenol) | 3.96 | 1.01 | 0.17 | 0.58 | 3.01 | -4.07 | *G. biloba* |
| Molecule1086 | 4-hydroxy-β-ionone | 19.08 | 0.59 | 0.07 | 0.19 | 2.10 | -2.46 | *G. biloba* |
| Molecule1087 | Terpinen-4-ol | 81.41 | 1.35 | 0.03 | 1.64 | 2.81 | -1.79 | *G. biloba, E. herba, S. miltiorrhiza, R. chuanxiong* |
| Molecule1088 | 5,6,7,7a-tetrahydro-4,4,7a-trimethy-2(4H)-benzofuranone | 83.79 | 1.22 | 0.05 | 1.39 | 2.25 | -1.72 | *G. biloba* |
| Molecule1089 | 5'-methoxybilobetin | 2.52 | -0.10 | 0.60 | -1.76 | 4.22 | -5.00 | *G. biloba* |
| Molecule1090 | 5-phenyldodecane | 4.99 | 1.91 | 0.08 | 2.03 | 8.15 | -7.13 | *G. biloba* |
| Molecule1091 | 6- octadecenoicacid, (Z) - | 33.13 | 1.18 | 0.14 | 1.03 | 7.72 | -6.39 | *G. biloba* |

Table S1: Continued

| Number | Compound | OB | Caco-2 | DL | BBB | Log*P* | Log*S* | Herbs |
| --- | --- | --- | --- | --- | --- | --- | --- | --- |
| Molecule1092 | 6,7-epoxy-octadecanoic acid,methyl ester | 24.21 | 1.06 | 0.21 | 0.64 | 6.71 | -6.50 | *G. biloba* |
| Molecule1093 | 6-heptadecenyl salicylic acid | 18.87 | 0.77 | 0.41 | 0.20 | 8.51 | -6.42 | *G. biloba* |
| Molecule1094 | 6-hydroxykynurenic acid | 49.30 | -0.17 | 0.09 | -0.64 | 1.39 | -1.53 | *G. biloba* |
| Molecule1095 | 6-methyl-5-octen-2-one | 24.22 | 1.37 | 0.02 | 1.58 | 2.65 | -2.06 | *G. biloba* |
| Molecule1096 | 6-methyl-3,5-heptadien-2-one | 19.01 | 1.35 | 0.01 | 1.51 | 2.01 | -1.62 | *G. biloba* |
| Molecule1097 | 6-phenyldodecane | 4.63 | 1.88 | 0.08 | 1.91 | 8.15 | -7.13 | *G. biloba* |
| Molecule1098 | Palmitoleic acid | 35.78 | 1.11 | 0.10 | 0.88 | 6.71 | -5.76 | *G. biloba, P. notoginseng, P. ginseng* |
| Molecule1099 | Methyl-oleate | 31.90 | 1.40 | 0.16 | 1.12 | 7.94 | -7.02 | *G. biloba, R. chuanxiong* |
| Molecule1100 | Alpha-methyl-benzene methanol | 54.75 | 1.16 | 0.02 | 1.35 | 1.58 | -0.90 | *G. biloba* |
| Molecule1101 | Amentoflavone | 2.95 | -0.27 | 0.65 | -1.72 | 4.06 | -5.01 | *G. biloba* |
| Molecule1102 | Anacaridic acid A | 15.94 | 0.76 | 0.31 | 0.37 | 8.13 | -6.06 | *G. biloba* |
| Molecule1103 | Anacaridic acid B | 20.18 | 1.03 | 0.32 | 0.61 | 7.76 | -6.08 | *G. biloba* |
| Molecule1104 | Anacaridic acid C | 24.08 | 1.12 | 0.32 | 0.61 | 7.28 | -5.93 | *G. biloba* |
| Molecule1105 | Anacaridic acid D | 26.78 | 1.09 | 0.33 | 0.59 | 6.74 | -6.15 | *G. biloba* |
| Molecule1106 | Anethole | 30.80 | 1.76 | 0.03 | 1.87 | 3.40 | -2.92 | *G. biloba* |
| Molecule1107 | Apigenin | 45.09 | 0.41 | 0.21 | -0.34 | 2.47 | -3.11 | *G. biloba, E. herba, S. miltiorrhiza, S. baicalensis, E. breviscapu* |
| Molecule1108 | Apigenin-7-O-β-D-glucoside | 9.68 | -1.20 | 0.74 | -2.13 | 0.17 | -2.45 | *G. biloba, S. baicalensis, E. breviscapu* |
| Molecule1109 | Benzaldehyde, 2-methyl- | 47.28 | 1.36 | 0.02 | 1.60 | 1.91 | -1.90 | *G. biloba* |
| Molecule1110 | Benzeneacetaldehyde | 40.00 | 1.35 | 0.02 | 1.81 | 1.75 | -1.76 | *G. biloba* |
| Molecule1111 | Benzyl-alcohol | 45.52 | 1.07 | 0.01 | 1.30 | 1.07 | -0.61 | *G. biloba, E. herba, R. chuanxiong* |
| Molecule1112 | β-eudesmol | 24.03 | 1.37 | 0.10 | 1.47 | 4.21 | -4.49 | *G. biloba, E. herba* |
| Molecule1113 | Bilobalide | 86.51 | -0.78 | 0.36 | -1.34 | 0.16 | -1.50 | *G. biloba* |
| Molecule1114 | Bilobanone | 81.90 | 1.24 | 0.10 | 1.29 | 3.90 | -3.91 | *G. biloba* |

Table S1: Continued

| Number | Compound | OB | Caco-2 | DL | BBB | Log*P* | Log*S* | Herbs |
| --- | --- | --- | --- | --- | --- | --- | --- | --- |
| Molecule1115 | Bilobetin | 2.46 | -0.13 | 0.63 | -1.60 | 4.36 | -4.98 | *G. biloba* |
| Molecule1116 | Bilobol | 3.35 | 1.37 | 0.24 | 1.03 | 8.00 | -5.98 | *G. biloba* |
| Molecule1117 | Butanoic acid | 22.12 | 0.70 | 0.00 | 0.94 | 0.78 | 0.43 | *G. biloba* |
| Molecule1118 | Caffeic acid | 45.99 | 0.24 | 0.05 | -0.13 | 1.67 | -2.05 | *S. miltiorrhiza, R. chuanxiong, G. biloba, E. breviscapu* |
| Molecule1119 | Caryophyllene oxide | 34.08 | 1.56 | 0.13 | 1.81 | 3.49 | -4.50 | *G. biloba, S. miltiorrhiza* |
| Molecule1120 | Caryophyllene | 29.23 | 1.83 | 0.09 | 2.08 | 5.35 | -4.83 | *G. biloba, E. herba, S. baicalensis, R. chuanxiong* |
| Molecule1121 | Catechin | 20.30 | -0.08 | 0.24 | -0.67 | 1.02 | -2.65 | *G. biloba* |
| Molecule1122 | Cedrol | 86.48 | 1.34 | 0.12 | 1.43 | 3.53 | -4.46 | *G. biloba* |
| Molecule1123 | Chlorogerdc acid | 18.60 | -1.07 | 0.33 | -1.71 | 0.17 | -2.01 | *G. biloba, S. miltiorrhiza, U. rhynchophylla, E. breviscapu* |
| Molecule1124 | Chrysoeriol-7-glucoside | 8.01 | -1.11 | 0.82 | -2.25 | 0.14 | -2.57 | *G. biloba* |
| Molecule1125 | Chrysoeriol | 46.45 | 0.46 | 0.27 | -0.25 | 2.53 | -3.36 | *G. biloba* |
| Molecule1126 | Cineole | 39.73 | 1.58 | 0.05 | 2.09 | 3.36 | -3.84 | *G. biloba, E. herba, S. baicalensis, R. chuanxiong* |
| Molecule1127 | Cinnamaldehyde | 30.59 | 1.37 | 0.02 | 1.47 | 2.00 | -2.51 | *G. biloba* |
| Molecule1128 | Cis- Farnesol | 22.37 | 1.28 | 0.06 | 1.09 | 4.84 | -3.58 | *G. biloba* |
| Molecule1129 | Oleic acid | 33.13 | 1.08 | 0.14 | 0.88 | 7.68 | -6.37 | *G. biloba, P. notoginseng, S. miltiorrhiza, R. chuanxiong* |
| Molecule1130 | Copaene | 29.59 | 1.83 | 0.12 | 2.13 | 3.75 | -5.05 | *G. biloba* |
| Molecule1131 | Cyanidin | 19.77 | 0.09 | 0.24 | -0.49 | 1.48 | -2.67 | *G. biloba, S. miltiorrhiza* |
| Molecule1132 | Cyclohexane | 54.12 | 1.76 | 0.01 | 2.16 | 3.46 | -3.33 | *G. biloba* |
| Molecule1133 | Cycloundecene(E) | 57.19 | 1.78 | 0.03 | 2.15 | 5.56 | -5.14 | *G. biloba* |

Table S1: Continued

| Number | Compound | OB | Caco-2 | DL | BBB | Log*P* | Log*S* | Herbs |
| --- | --- | --- | --- | --- | --- | --- | --- | --- |
| Molecule1134 | Dibutyl phthalate | 75.21 | 0.77 | 0.13 | 0.39 | 4.53 | -4.67 | *G. biloba, P. ginseng, E. herba, U. rhynchophylla* |
| Molecule1135 | Dihydroactinidiolide | 39.19 | 1.18 | 0.07 | 1.48 | 3.28 | -2.47 | *G. biloba* |
| Molecule1136 | Diisobutyl phthalate | 28.55 | 0.94 | 0.13 | 0.90 | 3.98 | -4.16 | *G. biloba, E. herba, U. rhynchophylla* |
| Molecule1137 | Dioctyl phthalate | 43.59 | 0.97 | 0.35 | 0.65 | 7.07 | -6.56 | *G. biloba* |
| Molecule1138 | Diosmetin | 50.41 | 0.36 | 0.27 | -0.50 | 2.52 | -3.34 | *G. biloba* |
| Molecule1139 | EdulanⅠ | 41.45 | 1.56 | 0.08 | 1.87 | 4.47 | -3.49 | *G. biloba* |
| Molecule1140 | Epicatechin | 32.16 | -0.11 | 0.24 | -0.67 | 1.02 | -2.65 | *G. biloba* |
| Molecule1141 | Epigallocatechin | 64.07 | -0.22 | 0.27 | -0.82 | 0.71 | -2.55 | *G. biloba* |
| Molecule1142 | Ethyl linoleate | 42.00 | 1.42 | 0.19 | 1.15 | 7.21 | -6.87 | *G. biloba, P. notoginseng, R. chuanxiong* |
| Molecule1143 | Eucarvone | 53.39 | 1.36 | 0.03 | 1.60 | 2.67 | -2.11 | *G. biloba* |
| Molecule1144 | E-α-hexenal | 46.01 | 1.29 | 0.01 | 1.66 | 1.80 | -1.49 | *G. biloba* |
| Molecule1145 | Farnecsyl acetone | 27.42 | 1.33 | 0.10 | 1.31 | 5.67 | -4.29 | *G. biloba* |
| Molecule1146 | Farnesol | 41.14 | 1.26 | 0.06 | 1.14 | 4.84 | -3.58 | *G. biloba, E. herba* |
| Molecule1147 | Farnesylacetone | 37.84 | 1.46 | 0.10 | 1.50 | 6.20 | -4.46 | *G. biloba* |
| Molecule1148 | Ferulic acid | 55.14 | 0.41 | 0.06 | 0.00 | 1.58 | -2.33 | *R. chuanxiong, G. biloba, E. breviscapu* |
| Molecule1149 | Hexahydrofarnesylacetone | 23.30 | 1.48 | 0.10 | 1.32 | 7.48 | -6.83 | *G. biloba, E. herba* |
| Molecule1150 | Flavoxanthin | 51.03 | 0.85 | 0.56 | -1.21 | 8.03 | -5.99 | *G. biloba* |
| Molecule1151 | Fluoranthene | 24.65 | 1.95 | 0.18 | 1.79 | 5.04 | -6.30 | *G. biloba* |
| Molecule1152 | Furfural | 34.81 | 0.97 | 0.01 | 1.21 | 0.43 | -0.79 | *G. biloba, E. herba, R. chuanxiong* |
| Molecule1153 | Gallocatechin-(4alpha-8)epicatechin | 17.66 | -1.34 | 0.61 | -2.55 | 2.22 | -3.53 | *G. biloba* |
| Molecule1154 | Gallocatechin | 17.94 | -0.26 | 0.27 | -0.97 | 0.71 | -2.55 | *G. biloba* |
| Molecule1155 | Gallocatechol | 25.08 | -0.22 | 0.27 | -0.82 | 0.71 | -2.55 | *G. biloba* |
| Molecule1156 | Genkwanin | 28.28 | 0.64 | 0.24 | -0.05 | 2.86 | -3.51 | *G. biloba* |

Table S1: Continued

| Number | Compound | OB | Caco-2 | DL | BBB | Log*P* | Log*S* | Herbs |
| --- | --- | --- | --- | --- | --- | --- | --- | --- |
| Molecule1157 | Geranylacetone | 22.45 | 1.44 | 0.04 | 1.56 | 4.59 | -3.31 | *G. biloba, E. herba* |
| Molecule1158 | Ginkgetin | 1.35 | 0.01 | 0.59 | -1.39 | 4.55 | -5.07 | *G. biloba* |
| Molecule1159 | Ginkgol | 17.77 | 1.76 | 0.21 | 1.76 | 8.76 | -6.95 | *G. biloba* |
| Molecule1160 | Ginkgolic acid | 20.18 | 1.05 | 0.32 | 0.75 | 7.76 | -6.08 | *G. biloba* |
| Molecule1161 | Ginkgolide A | 42.85 | -0.68 | 0.74 | -1.03 | 1.21 | -2.13 | *G. biloba* |
| Molecule1162 | Ginkgolide B | 44.38 | -1.22 | 0.73 | -1.55 | 0.49 | -1.91 | *G. biloba* |
| Molecule1163 | Ginkgolide C | 48.69 | -1.16 | 0.73 | -1.76 | 0.24 | -1.69 | *G. biloba* |
| Molecule1164 | Ginkgolide J | 44.60 | -1.18 | 0.74 | -1.59 | 0.23 | -1.79 | *G. biloba* |
| Molecule1165 | Ginkgolide M | 47.90 | -0.84 | 0.75 | -1.00 | 0.60 | -1.73 | *G. biloba* |
| Molecule1166 | Ginnol | 11.33 | 1.46 | 0.43 | 0.82 | 10.34 | -7.56 | *G. biloba* |
| Molecule1167 | Ginnone | 12.19 | 1.57 | 0.43 | 1.16 | 10.49 | -7.53 | *G. biloba* |
| Molecule1168 | Glucaric acid | 16.00 | -2.29 | 0.06 | -5.22 | -1.77 | -0.52 | *G. biloba* |
| Molecule1169 | Guaiacol | 72.14 | 1.35 | 0.02 | 1.62 | 1.32 | -0.27 | *G. biloba* |
| Molecule1170 | Heptadecanoicacid | 18.51 | 1.12 | 0.12 | 1.02 | 7.66 | -6.25 | *G. biloba, P. notoginseng* |
| Molecule1171 | Heptanal | 16.87 | 1.29 | 0.01 | 1.64 | 2.59 | -1.93 | *G. biloba, P. notoginseng, R. chuanxiong* |
| Molecule1172 | Heptane | 41.80 | 1.76 | 0.00 | 2.09 | 4.33 | -4.05 | *G. biloba, R. chuanxiong* |
| Molecule1173 | Hexadecane | 8.70 | 1.81 | 0.06 | 1.91 | 8.63 | -6.99 | *G. biloba, S. miltiorrhiza* |
| Molecule1174 | Hexane | 52.50 | 1.76 | 0.00 | 2.08 | 4.02 | -3.41 | *G. biloba* |
| Molecule1175 | Caproic acid | 23.20 | 0.83 | 0.01 | 1.02 | 1.88 | -1.08 | *G. biloba, P. notoginseng, E. herba, U. rhynchophylla* |
| Molecule1176 | Hydroginkgolic acid | 15.94 | 1.14 | 0.31 | 0.98 | 8.13 | -6.06 | *G. biloba* |
| Molecule1177 | Hydroginkgolinic acid | 16.43 | 1.14 | 0.27 | 0.96 | 7.75 | -5.77 | *G. biloba* |
| Molecule1178 | Isoamyl butyrate | 20.84 | 1.23 | 0.02 | 1.35 | 3.00 | -2.43 | *G. biloba* |
| Molecule1179 | Isoflavone | 37.04 | 1.20 | 0.13 | 1.14 | 3.10 | -4.24 | *G. biloba* |
| Molecule1180 | Isoginkgetin | 1.35 | 0.00 | 0.58 | -1.41 | 4.54 | -5.08 | *G. biloba* |
| Molecule1181 | Isogoycyrol | 39.05 | 0.89 | 0.83 | 0.00 | 4.13 | -3.98 | *G. biloba* |
| Molecule1182 | Isopentyl alcohol | 61.09 | 1.01 | 0.00 | 1.12 | 1.33 | -0.37 | *G. biloba* |
| Molecule1183 | Isophytol | 8.70 | 1.40 | 0.13 | 1.18 | 7.55 | -6.79 | *G. biloba* |

Table S1: Continued

| Number | Compound | OB | Caco-2 | DL | BBB | Log*P* | Log*S* | Herbs |
| --- | --- | --- | --- | --- | --- | --- | --- | --- |
| Molecule1184 | Isorhamnetin | 7.02 | 0.12 | 0.31 | -0.43 | 1.31 | -2.65 | *G. biloba* |
| Molecule1185 | Isorhamnetin-3-mono-beta-D-glucoside | 4.00 | -0.95 | 0.80 | -1.74 | 0.05 | -2.20 | *G. biloba* |
| Molecule1186 | Isorhamnetin-3-O-glucoside | 14.36 | -1.18 | 0.80 | -1.76 | 0.05 | -2.20 | *G. biloba* |
| Molecule1187 | Kaempferol 3-O-rhamnoside | 5.31 | -0.73 | 0.70 | -1.32 | 0.77 | -2.17 | *G. biloba* |
| Molecule1188 | Kaempferol | 42.30 | 0.28 | 0.24 | 0.02 | 1.23 | -2.47 | *E. herba, E. breviscapu, U. rhynchophylla, G. biloba* |
| Molecule1189 | Kaempferol-3-rhamnoglucoside | 7.92 | -1.84 | 0.73 | -2.55 | -0.20 | -1.96 | *G. biloba* |
| Molecule1190 | Kaempferol-7-rhamnoside | 39.54 | -0.96 | 0.72 | -2.07 | 0.41 | -2.25 | *G. biloba* |
| Molecule1191 | Lauric acid | 22.70 | 1.03 | 0.04 | 1.02 | 5.13 | -4.30 | *G. biloba, P. notoginseng, G. biloba, E. herba* |
| Molecule1192 | Leaf alcohol | 42.78 | 1.18 | 0.01 | 1.53 | 1.69 | -0.79 | *G. biloba, E. herba* |
| Molecule1193 | Linalool oxide | 64.13 | 1.05 | 0.04 | 1.18 | 1.51 | -2.05 | *G. biloba, E. herba* |
| Molecule1194 | Beta-linalool | 32.68 | 1.22 | 0.02 | 1.27 | 2.68 | -2.51 | *G. biloba, E. herba, S. miltiorrhiza, S. baicalensis, R. chuanxiong* |
| Molecule1195 | Linoleic acid | 41.90 | 1.15 | 0.14 | 0.92 | 7.06 | -6.26 | *G. biloba, P. notoginseng, E. herba, S. miltiorrhiza, R. chuanxiong* |
| Molecule1196 | linolenic acid ethyl ester | 46.10 | 1.45 | 0.20 | 1.02 | 6.77 | -6.83 | *G. biloba* |
| Molecule1197 | Linolenic acid | 45.01 | 1.09 | 0.15 | 0.99 | 6.62 | -6.02 | *G. biloba, E. herba* |
| Molecule1198 | Luteolin | 62.76 | 0.20 | 0.25 | -0.75 | 2.15 | -2.90 | *G. biloba, S. miltiorrhiza, E. breviscapu* |
| Molecule1199 | Luteolin-3′-glucoside | 8.65 | -1.33 | 0.80 | -2.20 | -0.03 | -2.40 | *G. biloba* |
| Molecule1200 | Luteolin-4′-glucoside | 41.97 | -1.35 | 0.79 | -2.22 | -0.01 | -2.42 | *G. biloba* |

Table S1: Continued

| Number | Compound | OB | Caco-2 | DL | BBB | Log*P* | Log*S* | Herbs |
| --- | --- | --- | --- | --- | --- | --- | --- | --- |
| Molecule1201 | Methionine | 82.14 | 0.06 | 0.01 | -0.17 | -1.85 | -0.80 | *G. biloba, S. miltiorrhiza* |
| Molecule1202 | Methyl eugenol | 57.98 | 1.56 | 0.04 | 1.59 | 2.99 | -2.98 | *G. biloba, R. chuanxiong* |
| Molecule1203 | Methyl hexadecanoate | 18.09 | 1.38 | 0.12 | 1.14 | 7.41 | -6.56 | *G. biloba, P. ginseng, P. notoginseng, E. herba, R. chuanxiong* |
| Molecule1204 | Methyl linoleate | 41.93 | 1.41 | 0.17 | 1.22 | 6.95 | -6.76 | *G. biloba, P. ginseng* |
| Molecule1205 | Methyl linolenate | 46.15 | 1.50 | 0.17 | 1.37 | 6.62 | -6.69 | *G. biloba* |
| Molecule1206 | Methylcyclopentane | 55.65 | 1.79 | 0.01 | 2.25 | 3.15 | -3.23 | *G. biloba* |
| Molecule1207 | Methylheptenone | 34.19 | 1.40 | 0.01 | 1.68 | 2.01 | -1.55 | *G. biloba* |
| Molecule1208 | Myricetin | 9.80 | -0.25 | 0.31 | -0.91 | 0.89 | -2.43 | *G. biloba* |
| Molecule1209 | Myristic acid | 21.18 | 1.06 | 0.07 | 1.03 | 6.10 | -5.12 | *G. biloba, P. notoginseng, E. herba* |
| Molecule1210 | Naphthalene | 27.55 | 1.88 | 0.03 | 1.93 | 3.33 | -3.29 | *G. biloba, E. herba* |
| Molecule1211 | Narcissin | 5.09 | -2.14 | 0.65 | -2.68 | -0.18 | -2.02 | *G. biloba* |
| Molecule1212 | Decanoicacid | 33.87 | 0.98 | 0.03 | 1.08 | 3.93 | -3.26 | *G. biloba, E. herba* |
| Molecule1213 | N-docosane | 8.37 | 1.88 | 0.18 | 1.83 | 10.16 | -7.76 | *G. biloba* |
| Molecule1214 | Nerlacetone | 19.04 | 1.46 | 0.04 | 1.58 | 4.59 | -3.31 | *G. biloba* |
| Molecule1215 | Nerolidol | 39.19 | 1.33 | 0.06 | 1.27 | 4.55 | -4.02 | *G. biloba, E. herba* |
| Molecule1216 | Heneicosane | 8.41 | 1.85 | 0.15 | 1.84 | 9.98 | -7.69 | *G. biloba, P. notoginseng, E. herba, S. miltiorrhiza* |
| Molecule1217 | Heptadecane | 7.34 | 1.81 | 0.07 | 1.78 | 9.01 | -7.23 | *G. biloba, P. notoginseng, E. herba* |
| Molecule1218 | N-hexacosane | 8.21 | 1.90 | 0.31 | 1.73 | 10.66 | -7.95 | *G. biloba* |
| Molecule1219 | N-hexacosanol | 10.95 | 1.45 | 0.35 | 0.72 | 9.98 | -7.45 | *G. biloba* |

Table S1: Continued

| Number | Compound | OB | Caco-2 | DL | BBB | Log*P* | Log*S* | Herbs |
| --- | --- | --- | --- | --- | --- | --- | --- | --- |
| Molecule1220 | Nonadecane | 8.52 | 1.87 | 0.11 | 1.85 | 9.55 | -7.51 | *G. biloba, E. herba* |
| Molecule1221 | N-octacosanol | 10.70 | 1.49 | 0.41 | 0.74 | 10.25 | -7.52 | *G. biloba, E. herba* |
| Molecule1222 | Octane | 29.72 | 1.77 | 0.01 | 2.08 | 4.73 | -4.53 | *G. biloba, P. ginseng, R. chuanxiong* |
| Molecule1223 | N-octanol | 21.14 | 1.14 | 0.01 | 1.22 | 3.21 | -2.39 | *G. biloba, E. herba* |
| Molecule1224 | Nonacosane | 43.54 | 1.77 | 0.00 | 2.16 | 2.81 | -1.91 | *G. biloba, E. herba* |
| Molecule1225 | Nonanal | 18.86 | 1.35 | 0.02 | 1.59 | 3.81 | -3.37 | *G. biloba, E. herba, R. chuanxiong* |
| Molecule1226 | Pentacosane | 8.25 | 1.91 | 0.28 | 1.82 | 10.55 | -7.90 | *G. biloba, S. miltiorrhiza* |
| Molecule1227 | Tetracosane | 8.28 | 1.91 | 0.24 | 1.77 | 10.43 | -7.87 | *G. biloba, E. herba* |
| Molecule1228 | Tricosane | 8.33 | 1.89 | 0.21 | 1.76 | 10.30 | -7.82 | *G. biloba, E. herba* |
| Molecule1229 | Octadecane | 8.58 | 1.87 | 0.09 | 1.86 | 9.31 | -7.38 | *G. biloba, P. notoginseng, E. herba, S. miltiorrhiza* |
| Molecule1230 | Stearic acid | 17.83 | 1.15 | 0.14 | 1.11 | 8.02 | -6.63 | *G. biloba, G. elata, P. notoginseng, E. herba, S. miltiorrhiza, S. baicalensis, E. breviscapu, R. chuanxiong* |
| Molecule1231 | Octanoic acid | 16.40 | 0.92 | 0.02 | 1.09 | 2.92 | -2.20 | *G. biloba, P. notoginseng, E. herba* |

Table S1: Continued

| Number | Compound | OB | Caco-2 | DL | BBB | Log*P* | Log*S* | Herbs |
| --- | --- | --- | --- | --- | --- | --- | --- | --- |
| Molecule1232 | O-cymene | 51.25 | 1.83 | 0.02 | 2.11 | 4.11 | -3.90 | *G. biloba, R. chuanxiong* |
| Molecule1233 | Paeonol | 30.98 | 0.91 | 0.04 | 0.93 | 1.60 | -1.75 | *G. biloba* |
| Molecule1234 | Eugenol | 44.47 | 1.36 | 0.04 | 1.42 | 2.66 | -2.06 | *G. biloba, S. baicalensis* |
| Molecule1235 | Ethyl palmitate | 17.70 | 1.38 | 0.18 | 1.05 | 8.59 | -6.99 | *G. biloba, P. notoginseng, E. herba, R. chuanxiong* |
| Molecule1236 | Palmitic acid | 19.30 | 1.10 | 0.10 | 1.04 | 7.23 | -5.80 | *G. biloba, P. notoginseng, G. elata, E. herba, S. miltiorrhiza, R. chuanxiong, P. ginseng* |
| Molecule1237 | P-coumaric acid | 44.94 | 0.47 | 0.04 | 0.11 | 1.74 | -2.21 | *G. biloba, E. herba* |
| Molecule1238 | P-cymene | 27.15 | 1.83 | 0.02 | 2.02 | 4.17 | -3.90 | *G. biloba, R. chuanxiong, S. miltiorrhiza, E. herba* |
| Molecule1239 | Pentadecanoic acid | 20.18 | 1.10 | 0.08 | 0.99 | 6.65 | -5.86 | *G. biloba, P. ginseng, E. herba* |
| Molecule1240 | Phenanthrene, 4- methyl- | 30.50 | 1.98 | 0.11 | 1.89 | 5.06 | -6.27 | *G. biloba* |
| Molecule1241 | Phenanthrene | 17.20 | 1.93 | 0.10 | 1.86 | 4.55 | -5.72 | *G. biloba, E. herba* |
| Molecule1242 | Phenethyl alcohol | 55.79 | 1.18 | 0.02 | 1.47 | 1.51 | -1.03 | *G. biloba, S. baicalensis, U. rhynchophylla* |
| Molecule1243 | Phenol, 4-(2-propenyl)- | 43.73 | 1.59 | 0.02 | 1.73 | 2.46 | -1.74 | *G. biloba* |
| Molecule1244 | Phenol | 36.05 | 1.48 | 0.01 | 1.85 | 1.39 | -0.31 | *G. biloba* |
| Molecule1245 | Phthalide | 63.49 | 1.13 | 0.03 | 1.27 | 0.99 | -1.29 | *G. biloba* |

Table S1: Continued

| Number | Compound | OB | Caco-2 | DL | BBB | Log*P* | Log*S* | Herbs |
| --- | --- | --- | --- | --- | --- | --- | --- | --- |
| Molecule1246 | P-hydroxybenzoic acid | 30.17 | 0.33 | 0.03 | 0.18 | 1.58 | -1.06 | *G. biloba, G. elata, E. herba, E. breviscapu, R. chuanxiong* |
| Molecule1247 | Phytol | 33.82 | 1.25 | 0.13 | 1.00 | 7.89 | -6.43 | *G. biloba, E. herba* |
| Molecule1248 | Pinitol | 22.30 | -1.54 | 0.05 | -3.81 | -2.67 | 0.45 | *G. biloba* |
| Molecule1249 | Protocatechuic acid | 25.40 | 0.14 | 0.04 | -0.01 | 1.32 | -1.10 | *G. biloba, E. herba, S. miltiorrhiza, E. breviscapu* |
| Molecule1250 | Pyrene | 25.05 | 1.92 | 0.19 | 1.76 | 5.19 | -6.94 | *G. biloba* |
| Molecule1251 | Pyridine | 42.32 | 1.47 | 0.01 | 1.92 | 0.70 | 0.49 | *G. biloba* |
| Molecule1252 | Quercitrin | 1.66 | -1.17 | 0.74 | -1.89 | 0.79 | -2.09 | *G. biloba, U. rhynchophylla* |
| Molecule1253 | Quercetin | 42.24 | 0.03 | 0.28 | 0.00 | 1.07 | -2.42 | *U. rhynchophylla, P. notoginseng, E. herba, E. breviscapu, G. biloba* |
| Molecule1254 | Quercitrin-2''-gallate | 3.01 | -1.83 | 0.68 | -2.60 | 2.27 | -2.74 | *G. biloba* |
| Molecule1255 | Quinic acid | 59.15 | -1.42 | 0.06 | -2.21 | -2.46 | 0.60 | *G. biloba* |
| Molecule1256 | Rutin | 3.20 | -2.50 | 0.68 | -3.04 | -0.14 | -1.86 | *G. biloba, E. herba, U. rhynchophylla* |
| Molecule1257 | Safranal | 39.81 | 1.42 | 0.04 | 1.80 | 2.93 | -2.04 | *G. biloba, E. herba* |
| Molecule1258 | Sciadopitysin | 0.89 | 0.17 | 0.54 | -1.23 | 4.49 | -5.17 | *G. biloba* |
| Molecule1259 | Sequoyitol | 20.64 | -1.57 | 0.05 | -3.91 | -2.67 | 0.45 | *G. biloba* |
| Molecule1260 | Sesamin | 10.90 | 0.76 | 0.83 | 0.19 | 2.25 | -4.20 | *G. biloba* |
| Molecule1261 | Shikimic acid | 42.68 | -1.36 | 0.04 | -3.66 | -1.70 | 0.07 | *G. biloba* |

Table S1: Continued

| Number | Compound | OB | Caco-2 | DL | BBB | Log*P* | Log*S* | Herbs |
| --- | --- | --- | --- | --- | --- | --- | --- | --- |
| Molecule1262 | Spathulenol | 81.61 | 1.40 | 0.12 | 1.58 | 2.75 | -3.84 | *G. biloba, P. notoginseng, S. miltiorrhiza, R. chuanxiong* |
| Molecule1263 | Stigmasterol | 43.83 | 1.31 | 0.76 | 0.77 | 6.95 | -7.18 | *G. biloba, G. elata, P. notoginseng, S. miltiorrhiza, S. baicalensis, E. breviscapu* |
| Molecule1264 | Syringetin | 35.12 | 0.05 | 0.37 | -0.59 | 1.59 | -2.80 | *G. biloba* |
| Molecule1265 | Syringetin-3-rutinoside | 8.59 | -1.80 | 0.58 | -2.47 | -0.11 | -2.08 | *G. biloba* |
| Molecule1266 | Tetradecane | 15.95 | 1.81 | 0.04 | 1.89 | 7.70 | -6.55 | *G. biloba, P. ginseng, E. herba, R. chuanxiong* |
| Molecule1267 | Thujopsene | 18.01 | 1.83 | 0.12 | 2.15 | 5.99 | -5.01 | *G. biloba* |
| Molecule1268 | Thymol | 42.91 | 1.61 | 0.03 | 1.89 | 3.16 | -2.37 | *G. biloba, E. herba, R. chuanxiong* |
| Molecule1269 | Trans-chalcone | 58.90 | 1.39 | 0.08 | 1.32 | 3.63 | -4.53 | *G. biloba* |
| Molecule1270 | Tricetin | 35.47 | 0.01 | 0.28 | -0.85 | 1.83 | -2.90 | *G. biloba* |
| Molecule1271 | Tricin | 29.00 | 0.53 | 0.34 | -0.20 | 2.54 | -3.52 | *G. biloba, E. herba* |
| Molecule1272 | Tridecanoic acid | 22.32 | 1.05 | 0.05 | 1.10 | 5.57 | -4.74 | *G. biloba, P. ginseng* |
| Molecule1273 | Vanillic acid | 59.94 | 0.39 | 0.04 | 0.12 | 1.70 | -1.47 | *G. biloba, E. herba, R. chuanxiong* |
| Molecule1274 | Vitamin C | 14.24 | -0.87 | 0.04 | -1.22 | -1.58 | 0.14 | *G. biloba* |
| Molecule1275 | Terpenol | 38.77 | 1.33 | 0.03 | 1.56 | 3.17 | -2.00 | *G. biloba, E. herba* |
| Molecule1276 | α-hexenal | 46.01 | 1.29 | 0.01 | 1.66 | 1.80 | -1.49 | *G. biloba* |
| Molecule1277 | α-ionone | 21.67 | 1.39 | 0.05 | 1.54 | 4.14 | -3.59 | *G. biloba, E. herba* |
| Molecule1278 | α-irone | 21.22 | 1.38 | 0.06 | 1.56 | 4.37 | -3.94 | *G. biloba* |

Table S1: Continued

| Number | Compound | OB | Caco-2 | DL | BBB | Log*P* | Log*S* | Herbs |
| --- | --- | --- | --- | --- | --- | --- | --- | --- |
| Molecule1279 | α-santalol | 23.34 | 1.32 | 0.13 | 1.27 | 3.93 | -4.15 | *G. biloba* |
| Molecule1280 | α-terpinene | 33.18 | 1.87 | 0.02 | 2.16 | 4.51 | -2.44 | *G. biloba, R. chuanxiong* |
| Molecule1281 | β-ionone epoxide | 21.24 | 1.07 | 0.09 | 1.11 | 3.20 | -3.64 | *G. biloba* |
| Molecule1282 | β-sesquiphellandrene | 17.21 | 1.88 | 0.06 | 2.06 | 5.71 | -5.00 | *G. biloba, R. chuanxiong* |
| Molecule1283 | Beta-sitosterol | 36.91 | 1.33 | 0.75 | 0.88 | 7.27 | -7.35 | *G. biloba, G. elata, P. notoginseng, S. miltiorrhiza, S. baicalensis, U. rhynchophylla, E. breviscapu, R. chuanxiong* |
| Molecule1284 | γ-linolenic acid | 45.01 | 1.10 | 0.14 | 0.89 | 6.59 | -6.04 | *G. biloba* |
| Molecule1285 | Hirsutine | 37.56 | 1.22 | 0.64 | 0.82 | 4.60 | -4.25 | *U. rhynchophylla* |

***molecule after deglycosylation**

Table S2. Active constituents of anti-stroke herbs and their corresponding ADME parameters

| Number | English Name | OB (%) | Caco-2 | DL | BBB | Log*P* | Log*S* | Herbs |
| --- | --- | --- | --- | --- | --- | --- | --- | --- |
| M_001 | Vanillic acid | 59.94 | 0.39 | 0.04 | 0.12 | 1.70 | -1.47 | *R. chuanxiong, G. elata, G. biloba* |
| M_002 | Vanillin | 69.24 | 0.67 | 0.03 | 0.56 | 1.31 | -1.48 | *G. elata, R. chuanxiong* |
| M_003 | Cnidilide | 77.55 | 1.21 | 0.07 | 1.41 | 3.37 | -3.47 | *R. chuanxiong* |
| M_004 | Myricanone | 57.61 | 0.67 | 0.51 | -0.08 | 3.95 | -4.51 | *R. chuanxiong* |
| M_005 | Spathulenol | 81.61 | 1.40 | 0.12 | 1.58 | 2.75 | -3.84 | *G. biloba, P. notoginseng, S. miltiorrhiza, R. chuanxiong* |
| M_006 | Globulol-(-)- | 81.84 | 1.33 | 0.12 | 1.45 | 3.15 | -4.72 | *R. chuanxiong* |
| M_007 | Ligustilide | 51.30 | 1.31 | 0.07 | 1.28 | 3.48 | -2.58 | *R. chuanxiong* |
| M_008 | Senkyunolide-A | 65.15 | 1.30 | 0.07 | 1.35 | 3.47 | -2.63 | *R. chuanxiong* |
| M_009 | 1,2,5,6-tetrahydrotanshinone | 45.68 | 1.00 | 0.36 | 0.40 | 3.33 | -4.37 | *S. miltiorrhiza* |
| M_010 | 1,2-dihydrotan-shinqiunone | 42.57 | 1.07 | 0.36 | 0.46 | 3.65 | -3.99 | *S. miltiorrhiza* |
| M_011 | 3α-hydroxytanshinone IIa | 50.10 | 0.52 | 0.44 | 0.22 | 3.14 | -3.97 | *S. miltiorrhiza* |
| M_012 | 3β-hydroxytanshinone IIa | 47.17 | 0.48 | 0.45 | -0.13 | 3.20 | -3.95 | *S. miltiorrhiza* |
| M_013 | Formyltanshinone | 74.09 | 0.53 | 0.42 | -0.28 | 3.85 | -4.11 | *S. miltiorrhiza* |
| M_014 | Salvianolic acid B | 3.01 | -1.68 | 0.41 | -2.52 | 3.93 | -4.51 | *S. miltiorrhiza* |
| M_015 | Tanshinal dehyde | 57.72 | 0.53 | 0.45 | -0.07 | 3.80 | -4.21 | *S. miltiorrhiza* |
| M_016 | Tanshinol II | 63.17 | 0.55 | 0.56 | 0.11 | 3.31 | -4.51 | *S. miltiorrhiza* |
| M_017 | Tanshinol I | 86.16 | 0.34 | 0.52 | -0.01 | 2.51 | -4.23 | *S. miltiorrhiza* |
| M_018 | Cryptotanshinone | 57.44 | 0.95 | 0.40 | 0.51 | 4.32 | -4.48 | *S. miltiorrhiza* |
| M_019 | Dihydrotanshinone I | 50.04 | 0.96 | 0.36 | 0.43 | 3.63 | -4.56 | *S. miltiorrhiza* |
| M_020 | Isocryptotanshinone | 60.08 | 0.93 | 0.39 | 0.34 | 4.18 | -4.42 | *S. miltiorrhiza* |
| M_021 | Isotanshinone IIb | 61.64 | 0.37 | 0.45 | -0.33 | 3.10 | -3.86 | *S. miltiorrhiza* |
| M_022 | Isotanshinone IIa | 55.09 | 1.02 | 0.40 | 0.45 | 4.11 | -4.34 | *S. miltiorrhiza* |
| M_023 | Miltionone II | 70.17 | 0.62 | 0.44 | 0.03 | 2.59 | -3.68 | *S. miltiorrhiza* |
| M_024 | Nortanshinone | 40.00 | 0.48 | 0.37 | -0.27 | 3.18 | -3.70 | *S. miltiorrhiza* |
| M_025 | Salvianic acid A | 78.22 | -0.27 | 0.06 | -0.62 | 0.77 | -1.47 | *S. miltiorrhiza* |
| M_026 | Salvianolic acid g | 51.90 | -0.14 | 0.61 | -0.97 | 2.38 | -3.40 | *S. miltiorrhiza* |
| M_027 | Tanshinone IIa | 20.32 | 1.04 | 0.40 | 0.70 | 4.10 | -4.45 | *S. miltiorrhiza* |
| M_028 | Tanshinone IIb | 70.19 | 0.43 | 0.45 | -0.30 | 3.31 | -4.01 | *S. miltiorrhiza* |
| M_029 | Tanshinone I | 29.32 | 1.04 | 0.36 | 0.53 | 3.38 | -4.38 | *S. miltiorrhiza* |
| M_030 | △1-dehydrotanshinone | 48.67 | 1.07 | 0.40 | 0.60 | 4.12 | -4.50 | *S. miltiorrhiza* |

Table S2: Continued

| Number | English Name | OB (%) | Caco-2 | DL | BBB | Log*P* | Log*S* | Herbs |  |
| --- | --- | --- | --- | --- | --- | --- | --- | --- | --- |
| M_031 | Apigenin-7-O-β-D-glucuronide | 9.68 | -1.20 | 0.74 | -2.13 | 0.17 | -2.45 | *E. breviscapu* | |
| M_032 | Caffeic acid | 45.99 | 0.24 | 0.05 | -0.13 | 1.67 | -2.05 | *S. miltiorrhiza, R. chuanxiong, G. biloba, E. breviscapu* | |
| M_033 | Ferulic acid | 55.14 | 0.41 | 0.06 | 0.00 | 1.58 | -2.33 | *R. chuanxiong, G. biloba, E. breviscapu* | |
| M_034* | Scutellarin_DG | 45.56 | 0.31 | 0.21 | -0.36 | 2.22 | -2.97 | *E. breviscapu, S. baicalensis* | |
| M_035 | Apigenin | 45.09 | 0.41 | 0.21 | -0.34 | 2.47 | -3.11 | *G. biloba, E. herba, S. miltiorrhiza, S. baicalensis, E. breviscapu* | |
| M_036* | 5,6,4'-trihydroxyflavone-7-O-β-D-galactonic acid_DG | 38.91 | 0.33 | 0.76 | -0.55 | 2.22 | -2.97 | *E. breviscapu* | |
| M_037 | 3,5,6,4’-tetrahydroxy-7-methoxyflavonoid | 55.25 | 0.37 | 0.30 | -0.39 | 1.36 | -2.61 | *E. breviscapu* | |
| M_038 | Naringenin | 39.62 | 0.34 | 0.21 | -0.21 | 2.47 | -3.11 | *E. breviscapu* | |
| M_039 | 1-hydroxy-2,3,5-trimethoxyxanthone | 102.76 | 1.06 | 0.30 | 0.30 | 2.96 | -3.49 | *E. breviscapu* | |
| M_040* | 5,4’-dihydroxyflavonoid-7-O-β-D-pyranglucuronatebuthylester_DG | 47.87 | 0.43 | 0.72 | -0.33 | 2.47 | -3.11 | *E. breviscapu* | |
| M_041 | 4’-hydroxybaicalein | 59.56 | 0.31 | 0.24 | -0.43 | 2.22 | -2.97 | *E. breviscapu* | |
| M_042* | Erigeside A_DG | 45.56 | 0.34 | 0.21 | 0.05 | 1.46 | -1.78 | *E. breviscapu* | |
| M_043 | Nemerosin | 51.78 | 0.83 | 0.65 | -0.09 | 3.48 | -4.96 | *U. rhynchophylla* | |
| M_044 | Ajmalicine | 55.19 | 0.94 | 0.81 | 0.33 | 3.41 | -3.54 | *U. rhynchophylla* | |
| M_045 | Angustidine | 41.37 | 1.15 | 0.66 | 0.16 | 2.93 | -3.63 | *U. rhynchophylla* | |
| M_046 | L-epicatechin | 53.89 | 0.01 | 0.24 | -0.65 | 1.02 | -2.65 | *U. rhynchophylla* | |
| M_047 | Geissoschizinc acid | 42.27 | 0.44 | 0.60 | 0.12 | 3.69 | -3.58 | *U. rhynchophylla* | |
| M_048 | Vallesiachotamine | 53.45 | 1.01 | 0.60 | 0.62 | 3.05 | -3.85 | *U. rhynchophylla* | |
| M_049 | Pteropodine | 62.34 | 0.09 | 0.75 | -0.19 | 2.31 | -3.06 | *U. rhynchophylla* | |
| M_050 | Hirsutine | 37.56 | 1.22 | 0.64 | 0.82 | 4.60 | -4.25 | *U. rhynchophylla* | |
| M_051 | Rhynchophylline A | 37.15 | 0.40 | 0.69 | -0.15 | 2.29 | -3.19 | *U. rhynchophylla* | |

Table S2: Continued

| Number | English Name | OB (%) | Caco-2 | DL | BBB | Log*P* | Log*S* | Herbs |
| --- | --- | --- | --- | --- | --- | --- | --- | --- |
| M_052 | Geissoschizinc | 48.77 | 0.70 | 0.64 | 0.37 | 3.84 | -4.12 | *U. rhynchophylla* |
| M_053 | Rhynchophylline C | 62.34 | 0.09 | 0.75 | -0.19 | 2.31 | -3.06 | *U. rhynchophylla* |
| M_054 | Rhynchophylline | 47.86 | 0.70 | 0.57 | 0.38 | 2.85 | -3.66 | *U. rhynchophylla* |
| M_055 | Rhynchophylline N-oxide | 51.19 | 0.58 | 0.57 | 0.33 | 2.85 | -3.66 | *U. rhynchophylla* |
| M_056 | Akuammigine | 65.04 | 0.72 | 0.81 | 0.54 | 3.41 | -3.54 | *U. rhynchophylla* |
| M_057 | Corynoxine | 38.51 | 0.63 | 0.57 | 0.33 | 2.85 | -3.66 | *U. rhynchophylla* |
| M_058 | Corynoxine B | 54.47 | 0.64 | 0.57 | 0.25 | 2.85 | -3.66 | *U. rhynchophylla* |
| M_059 | Hirsuteine | 56.27 | 0.92 | 0.64 | 0.61 | 3.33 | -4.04 | *U. rhynchophylla* |
| M_060 | Kaempferol | 42.30 | 0.28 | 0.24 | 0.02 | 1.23 | -2.47 | *E. herba, E. breviscapu, U. rhynchophylla, G. biloba* |
| M_061 | (2S,12bR)-methyl2-((E)-1-oxobut-2-en-2-yl)-1,2,6,7,12,12b-hexahydroindolo[2,3-a]quinolizine-3-carboxylate | 42.82 | 1.08 | 0.60 | 0.63 | 3.05 | -3.85 | *U. rhynchophylla* |
| M_062 | β-yohimbine | 49.15 | 0.44 | 0.81 | -0.10 | 2.36 | -3.01 | *U. rhynchophylla* |
| M_063 | Isorhynchophylline | 49.31 | 0.29 | 0.52 | -0.21 | 2.47 | -3.36 | *U. rhynchophylla* |
| M_064 | Geissoschizine methyl ether | 32.70 | 0.97 | 0.64 | 0.66 | 3.84 | -4.12 | *U. rhynchophylla* |
| M_065 | Corynantheine | 56.27 | 0.93 | 0.64 | 0.51 | 3.33 | -4.04 | *U. rhynchophylla* |
| M_066 | Quercetin | 42.24 | 0.03 | 0.28 | 0.00 | 1.07 | -2.42 | *G. biloba, P. notoginseng, E. herba, U. rhynchophylla, E. breviscapu* |
| M_067* | Cadambine_DG | 99.89 | 0.15 | 0.84 | -0.34 | 1.81 | -2.39 | *U. rhynchophylla* |
| M_068 | Angustine | 32.91 | 1.14 | 0.71 | 0.46 | 2.79 | -3.97 | *U. rhynchophylla* |
| M_069 | Isorhynchophyllic acid | 46.90 | 0.29 | 0.52 | -0.21 | 2.47 | -3.36 | *U. rhynchophylla* |
| M_070 | Isocorynantheic acid | 50.32 | 0.77 | 0.60 | 0.26 | 3.32 | -3.63 | *U. rhynchophylla* |
| M_071* | Arzelin_DG | 71.18 | 0.25 | 0.24 | -0.47 | 1.23 | -2.47 | *U. rhynchophylla* |
| M_072 | Ginsenoside CK | 6.51 | -3.39 | 0.04 | -0.44 | 3.73 | -4.62 | *P. notoginseng, P. ginseng* |
| M_073 | (tans)-5,7,2',6'-Tetrahydroxyflavanonol | 59.25 | -0.33 | 0.27 | -0.84 | 1.01 | -2.21 | *S. baicalensis* |
| M_074 | 2,6,2',4'-Tetrahydroxy-6'-methoxychalcone | 83.46 | 0.22 | 0.22 | -0.43 | 2.71 | -3.62 | *S. baicalensis* |

Table S2: Continued

| Number | English Name | OB (%) | Caco-2 | DL | BBB | Log*P* | Log*S* | Herbs |
| --- | --- | --- | --- | --- | --- | --- | --- | --- |
| M_075 | Tenaxin-I | 24.00 | 0.96 | 0.72 | 0.19 | 2.04 | -3.50 | *S. baicalensis* |
| M_076 | Acacetin | 34.93 | 0.62 | 0.35 | 0.00 | 2.95 | -3.51 | *S. baicalensis* |
| M_077 | Cineole | 39.73 | 1.58 | 0.05 | 2.09 | 3.36 | -3.84 | *G. biloba, E. herba, S. baicalensis, R. chuanxiong* |
| M_078* | Dihydrobaicalin_DG | 40.24 | 0.55 | 0.75 | 0.18 | 2.66 | -2.98 | *S. baicalensis* |
| M_079 | Eriodictyol | 60.85 | 0.07 | 0.23 | -0.66 | 2.15 | -2.90 | *S. baicalensis, E. breviscapu* |
| M_080 | Eugenol | 44.47 | 1.36 | 0.04 | 1.42 | 2.66 | -2.06 | *G. biloba, S. baicalensis* |
| M_081 | Phenethyl alcohol | 55.79 | 1.18 | 0.02 | 1.47 | 1.51 | -1.03 | *G. biloba, S. baicalensis, U. rhynchophylla* |
| M_082 | Viscidulin I | 41.43 | 0.02 | 0.35 | -0.42 | 1.01 | -2.21 | *S. baicalensis* |
| M_083 | Viscidulin II | 73.67 | 0.48 | 0.27 | -0.11 | 2.47 | -3.29 | *S. baicalensis* |
| M_084 | 3',5,6',7-tetrahydroxy-2',8-dimethoxyflavone | 53.58 | 0.25 | 0.23 | -0.57 | 2.19 | -3.13 | *S. baicalensis* |
| M_085 | Beta-linalool | 32.68 | 1.22 | 0.02 | 1.27 | 2.68 | -2.51 | *G. biloba, E. herba, S. miltiorrhiza, S. baicalensis, R. chuanxiong* |
| M_086* | Darendoside A_DG | 65.04 | 0.61 | 0.67 | 0.21 | 0.74 | -1.12 | *S. baicalensis* |
| M_087 | 5,7,2',6'-Tetrahydroxyflavone | 59.28 | 0.20 | 0.37 | -0.56 | 2.04 | -2.84 | *S. baicalensis* |
| M_088 | Menthone | 57.79 | 1.35 | 0.24 | 1.70 | 2.65 | -2.77 | *S. baicalensis* |
| M_089 | Oroxylin A | 45.59 | 0.77 | 0.44 | 0.13 | 3.01 | -3.34 | *S. baicalensis* |
| M_090 | 5,7,4'-trihydroxy-8-methoxyflavone | 38.33 | 0.45 | 0.45 | -0.40 | 2.54 | -3.30 | *S. baicalensis* |
| M_091 | Wogonin | 20.71 | 0.79 | 0.02 | 0.10 | 2.97 | -3.36 | *S. baicalensis* |
| M_092 | Baicalein | 23.77 | 0.58 | 0.44 | -0.05 | 2.66 | -2.98 | *S. baicalensis, E. breviscapu* |
| M_093 | Baicalin | 13.16 | -1.05 | 0.23 | -1.97 | 0.55 | -2.23 | *S. miltiorrhiza, S. baicalensis* |
| M_094 | 5,2',6'-trihydroxy-7,8-dimethoxyflavone | 48.71 | 0.45 | 0.33 | -0.10 | 2.47 | -3.29 | *S. baicalensis* |

Table S2: Continued

| Number | English Name | OB (%) | Caco-2 | DL | BBB | Log*P* | Log*S* | Herbs |
| --- | --- | --- | --- | --- | --- | --- | --- | --- |
| M_095 | 5,7,2'-trihydroxy-8,6'-dimethoxyflavone | 43.84 | 0.44 | 0.34 | -0.15 | 2.48 | -3.32 | *S. baicalensis* |
| M_096 | 5,2',5'-trihydroxy-6,7,8-trimethoxyflavone | 34.47 | 0.34 | 0.40 | -0.48 | 2.29 | -3.36 | *S. baicalensis* |
| M_097 | (2S)-7,2',6'-trihydroxy-5-methoxyflavanone | 63.69 | 0.26 | 0.27 | -0.26 | 2.43 | -3.19 | *S. baicalensis* |
| M_098 | 5,7,4'-trihydroxy-6-methoxyflavanone | 55.41 | 0.34 | 0.27 | -0.32 | 2.56 | -3.31 | *S. baicalensis* |
| M_099 | 5,8,2'-trihydroxy-7-methoxyflavone | 61.85 | 0.60 | 0.27 | -0.01 | 2.51 | -3.12 | *S. baicalensis* |
| M_100 | 5,7,2'-trihydroxy-8-methoxyflavone | 44.85 | 0.57 | 0.27 | 0.08 | 2.51 | -3.15 | *S. baicalensis* |
| M_101 | 3,5,7,2',6'-pentahydroxyflavanone | 68.45 | -0.27 | 0.27 | -0.80 | 1.01 | -2.21 | *S. baicalensis* |
| M_102 | Rehderianin I | 36.31 | 0.44 | 0.33 | -0.25 | 2.50 | -3.37 | *S. baicalensis* |
| M_103 | Ganhuangenin | 47.07 | 0.26 | 0.37 | -0.39 | 2.19 | -3.13 | *S. baicalensis* |
| M_104 | 5,8-Dihydroxy-6,7-dimethoxyflavone | 44.41 | 0.96 | 0.23 | 0.09 | 2.71 | -3.40 | *S. baicalensis* |
| M_105 | 7-methoxybaicalein | 32.65 | 0.78 | 0.23 | 0.34 | 3.09 | -3.37 | *S. baicalensis* |
| M_106 | (2R,3R)-2',3,5,7-tetrahydroxyflavanone | 33.88 | -0.29 | 0.24 | -0.70 | 1.18 | -2.30 | *S. baicalensis* |
| M_107 | (2S)-2',5,6',7-tetrahydroxyflavanone | 63.59 | 0.16 | 0.24 | -0.29 | 2.04 | -2.84 | *S. baicalensis* |
| M_108 | Rivularin(flavone) | 50.09 | 0.67 | 0.37 | 0.12 | 2.10 | -3.49 | *S. baicalensis* |
| M_109 | Moslosooflavone | 40.78 | 1.00 | 0.25 | 0.57 | 3.00 | -3.70 | *S. baicalensis* |
| M_110 | 5-methoxy-7-hydroxyflaconone | 45.49 | 0.82 | 0.29 | 0.26 | 3.38 | -3.45 | *S. baicalensis* |
| M_111 | Skullcapflavone II | 61.22 | 0.63 | 0.44 | 0.00 | 1.76 | -3.45 | *S. baicalensis* |
| M_112* | Scuteamoenoside_DG | 69.58 | 0.34 | 0.27 | -0.08 | 2.45 | -3.16 | *S. baicalensis* |
| M_113 | Scutevurin | 2.64 | -1.10 | 0.33 | -2.13 | 1.21 | -2.22 | *S. baicalensis, E. breviscapu* |
| M_114 | 5,7,2'-trihydroxy-6-methoxyflavone | 34.97 | 0.49 | 0.27 | 0.04 | 2.53 | -3.13 | *S. baicalensis* |
| M_115 | 5,2',6'-trihydroxy-6,7-dimethoxyflavone-2'-O-D-glucoside | 4.08 | -1.28 | 0.86 | -2.15 | 0.87 | -2.40 | *S. baicalensis* |
| M_116 | Savligenin | 48.73 | 0.85 | 0.33 | -0.08 | 1.95 | -3.87 | *S. baicalensis* |

Table S2: Continued

| Number | English Name | OB (%) | Caco-2 | DL | BBB | Log*P* | Log*S* | Herbs |
| --- | --- | --- | --- | --- | --- | --- | --- | --- |
| M_117 | 5,7-dihydroxy-6,8,2',3'-tetramethoxyflavone | 69.65 | 0.70 | 0.44 | 0.10 | 1.80 | -3.58 | *S. baicalensis* |
| M_118 | 6,2'-dihydroxy-5,7,8,6'-tetramethoxyflavone | 38.58 | 0.58 | 0.44 | 0.11 | 1.75 | -3.49 | *S. baicalensis* |
| M_119 | Ephedrine | 45.21 | 1.02 | 0.03 | 1.11 | 1.00 | -1.30 | *E. herba* |
| M_120 | Norpseudoephedrine | 74.13 | 0.53 | 0.03 | 0.54 | 0.57 | -0.87 | *E. herba* |
| M_121 | Ligustrazine | 29.64 | 1.19 | 0.03 | 1.06 | 1.46 | -0.96 | *E. herba, R. chuanxiong* |
| M_122 | Pseudoephedrine | 40.82 | 1.10 | 0.03 | 1.23 | 1.00 | -1.30 | *E. herba* |
| M_123 | N-methylephedrine | 37.82 | 1.03 | 0.04 | 1.28 | 1.73 | -1.07 | *E. herba* |
| M_124 | Leucopelargonidin | 58.03 | -0.14 | 0.24 | -0.62 | 0.62 | -2.20 | *E. herba* |
| M_125 | Herbacetin | 37.54 | 0.13 | 0.27 | -0.74 | 1.08 | -2.37 | *E. herba* |
| M_126 | Methylephedrine | 32.87 | 1.04 | 0.04 | 1.37 | 1.73 | -1.07 | *E. herba* |
| M_127 | Methylpseudoephedrine | 40.34 | 1.12 | 0.04 | 1.37 | 1.73 | -1.07 | *E. herba* |
| M_128 | Rutin | 3.20 | -2.50 | 0.68 | -3.04 | -0.14 | -1.86 | *G. biloba, E. herba, U. rhynchophylla* |
| M_129 | Leucocyanidin | 41.03 | -0.30 | 0.27 | -0.95 | 0.44 | -2.14 | *E. herba* |
| M_130 | Frutinone A | 66.05 | 0.89 | 0.34 | 0.46 | 2.80 | -2.81 | *P. ginseng* |
| M_131 | Ginsenoside Rb1 | 6.29 | -3.72 | 0.04 | -4.95 | -0.24 | -3.02 | *P. notoginseng, P. ginseng* |
| M_132 | N,N-Dimethyldecanamide | 55.50 | 1.51 | 0.75 | 1.72 | 3.75 | -3.12 | *P. ginseng* |
| M_133 | Protopine | 57.53 | 0.84 | 0.78 | 0.21 | 1.95 | -3.24 | *P. ginseng* |
| M_134 | Suchilactone | 57.52 | 0.82 | 0.24 | 0.28 | 3.45 | -4.89 | *P. ginseng* |
| M_135 | Beta-santalol | 35.30 | 1.28 | 0.76 | 1.25 | 4.42 | -3.91 | *P. ginseng* |
| M_136 | Ethyl linoleate | 42.00 | 1.42 | 0.19 | 1.15 | 7.21 | -6.87 | *G. biloba, P. notoginseng, R. chuanxiong* |
| M_137 | Ginsenoside F1 | 4.05 | -1.20 | 0.60 | -2.12 | 2.68 | -3.94 | *P. notoginseng, P. ginseng* |
| M_138 | Ginsenoside Rc | 8.12 | -3.85 | 0.04 | -5.22 | -0.17 | -3.14 | *P. notoginseng, P. ginseng* |
| M_139 | Ginsenoside Rd | 5.42 | -2.88 | 0.09 | -4.20 | 0.73 | -3.39 | *P. notoginseng, P. ginseng* |
| M_140 | Ginsenoside Re | 5.43 | -2.96 | 0.12 | -4.24 | 1.13 | -3.28 | *P. notoginseng, P. ginseng* |

Table S2: Continued

| Number | English Name | OB (%) | Caco-2 | DL | BBB | Log*P* | Log*S* | Herbs |
| --- | --- | --- | --- | --- | --- | --- | --- | --- |
| M_141 | Ginsenoside Rg1 | 17.74 | -2.26 | 0.28 | -3.42 | 1.00 | -3.42 | *P. notoginseng, P. ginseng* |
| M_142 | Ginsenoside Rh2 | 6.54 | -0.83 | 0.56 | -1.73 | 3.77 | -4.71 | *P. notoginseng, P. ginseng* |
| M_143 | Glycyrrhizin | 35.00 | 0.51 | 0.18 | -0.22 | 2.79 | -3.28 | *P. notoginseng* |
| M_144 | Protopanaxatriol | 12.65 | 0.05 | 0.78 | -0.65 | 4.21 | -4.48 | *P. notoginseng, P. ginseng* |
| M_145 | Protopanoxadiol | 29.61 | 0.42 | 0.78 | -0.29 | 4.74 | -5.01 | *P. notoginseng, P. ginseng* |
| M_146 | Vanillyl alcohol | 37.70 | 0.56 | 0.03 | 0.24 | 0.50 | -0.72 | *G. elata* |
| M_147 | β-sitosterol | 36.91 | 1.33 | 0.75 | 0.88 | 7.27 | -7.35 | *G. biloba, G. elata, P. notoginseng, S. miltiorrhiza, S. baicalensis, U. rhynchophylla, E. breviscapu, R. chuanxiong* |
| M_148 | Stigmasterol | 43.83 | 1.31 | 0.76 | 0.77 | 6.95 | -7.18 | *G. biloba, G. elata, P. notoginseng, S. miltiorrhiza, S. baicalensis, E. breviscapu* |
| M_149 | Cymbinodin A | 50.26 | 0.71 | 0.21 | 0.28 | 3.04 | -3.70 | *G. elata* |
| M_150* | Gastrodin_DG | 55.19 | 0.61 | 0.02 | 0.29 | 0.53 | -0.32 | *G. elata* |
| M_151 | Chrysoeriol | 46.45 | 0.46 | 0.27 | -0.25 | 2.53 | -3.36 | *G. biloba* |
| M_152 | Diosmetin | 50.41 | 0.36 | 0.27 | -0.50 | 2.52 | -3.34 | *G. biloba* |
| M_153 | Epigallocatechin | 64.07 | -0.22 | 0.27 | -0.82 | 0.71 | -2.55 | *G. biloba* |
| M_154 | Ginkgolide A | 42.85 | -0.68 | 0.74 | -1.03 | 1.21 | -2.13 | *G. biloba* |
| M_155 | Ginkgolide B | 44.38 | -1.22 | 0.73 | -1.55 | 0.49 | -1.91 | *G. biloba* |
| M_156 | Isogoycyrol | 39.05 | 0.89 | 0.83 | 0.00 | 4.13 | -3.98 | *G. biloba* |
| M_157 | Luteolin | 62.76 | 0.20 | 0.25 | -0.75 | 2.15 | -2.90 | *G. biloba, S. miltiorrhiza, E. breviscapu, S. baicalensis* |

Table S2: Continued

| Number | English Name | OB (%) | Caco-2 | DL | BBB | Log*P* | Log*S* | Herbs |
| --- | --- | --- | --- | --- | --- | --- | --- | --- |
| M_158 | Syringetin | 35.12 | 0.05 | 0.37 | -0.59 | 1.59 | -2.80 | *G. biloba* |
| M_159 | Tricetin | 35.47 | 0.01 | 0.28 | -0.85 | 1.83 | -2.90 | *G. biloba* |
| M_160 | Myricetin | 9.80 | -0.25 | 0.31 | -0.91 | 0.89 | -2.43 | *G. biloba* |
| M_161 | Carthamidin | 43.78 | 0.16 | 0.72 | -0.42 | 2.22 | -2.97 | *S. baicalensis* |
| M_162 | Isopteropodine | 69.54 | 0.38 | 0.75 | 0.09 | 2.31 | -3.06 | *U. rhynchophylla* |
| M_163 | Tetrahydroalstonine | 41.30 | 1.13 | 0.81 | 0.69 | 4.03 | -3.71 | *U. rhynchophylla* |
| M_164 | Tanshindiol a | 74.54 | 0.02 | 0.46 | -0.74 | 2.06 | -3.21 | *S. miltiorrhiza* |
| M_165 | Przewaquinone c | 60.99 | 0.41 | 0.40 | -0.30 | 2.89 | -3.82 | *S. miltiorrhiza* |
| M_166 | Ditertbutyl phthalate | 67.02 | 0.86 | 0.13 | 0.77 | 4.32 | -4.57 | *P. notoginseng, P. ginseng* |
| M_167 | 3,6,7,2',6'-pentahydroxyflavanone | 64.47 | -0.30 | 0.27 | -0.74 | 1.00 | -2.28 | *S. baicalensis* |
| M_168* | 5,2',6'-trihydroxy-6,7,8-trimethoxyflavone-2'-O-D-glucoside_DG | 46.10 | 0.38 | 0.40 | -0.23 | 2.22 | -3.28 | *S. baicalensis* |
| M_169 | Senkyunolide-K | 61.80 | 0.52 | 0.08 | 0.30 | 2.13 | -1.84 | *R. chuanxiong* |
| M_170 | Erigeside II | 34.27 | -0.43 | 0.28 | -1.02 | 0.38 | -1.72 | *E. breviscapu* |
| M_171* | 3α-dihydrocadambine_DG | 65.70 | 0.12 | 0.88 | -0.27 | 1.36 | -2.20 | *U. rhynchophylla* |
| M_172 | Mitraphyllic acid | 31.70 | 0.02 | 0.70 | -0.25 | 1.67 | -2.57 | *U. rhynchophylla* |
| M_173 | Epicatechin | 32.16 | -0.11 | 0.24 | -0.67 | 1.02 | -2.65 | *G. biloba* |
| M_174* | Trifolirhizin_DG | 82.10 | 0.88 | 0.64 | 0.13 | 2.19 | -2.75 | *P. ginseng* |
| M_175 | 2,2-Dimethylbutane | 37.81 | 1.78 | 0.39 | 2.18 | 3.74 | -2.97 | *P. ginseng* |
| M_176 | Rhynchophylline E | 79.92 | 0.28 | 0.75 | -0.24 | 2.31 | -3.06 | *U. rhynchophylla* |
| M_177 | Uncarine F | 68.17 | 0.49 | 0.75 | 0.17 | 2.31 | -3.06 | *U. rhynchophylla* |
| M_178 | Isoformosanine | 122.86 | 0.34 | 0.75 | 0.06 | 2.31 | -3.06 | *U. rhynchophylla* |
| M_179 | Angustoline | 33.15 | 0.41 | 0.77 | -0.31 | 1.84 | -3.28 | *U. rhynchophylla* |
| M_180 | 2',5,8-trihydroxy-6,7-dimethoxyflavone | 63.69 | 0.47 | 0.33 | -0.12 | 3.01 | -3.34 | *S. baicalensis* |
| M_181 | Perlolyrine | 67.82 | 0.88 | 0.27 | 0.15 | 2.66 | -3.47 | *R. chuanxiong* |
| M_182 | Alpha-cadinol | 31.69 | 1.30 | 0.22 | 1.27 | 3.52 | -3.60 | *P. ginseng, S. miltiorrhiza* |
| M_183 | Malkangunin | 57.61 | 0.22 | 0.56 | -0.17 | 2.72 | -3.76 | *P. ginseng* |
| M_184 | Deoxyharringtonine | 39.27 | 0.19 | 0.31 | -0.25 | 2.96 | -3.95 | *P. ginseng* |
| M_185 | Methylenetanshinone | 42.36 | 1.03 | 0.36 | 0.46 | 3.12 | -4.05 | *S. miltiorrhiza* |
| M_186 | 2,4-bis(4-hydroxybenzyl)phenol | 30.49 | 1.00 | 0.28 | 0.08 | 3.75 | -4.45 | *G. elata* |

Table S2: Continued

| Number | English Name | OB (%) | Caco-2 | DL | BBB | Log*P* | Log*S* | Herbs |
| --- | --- | --- | --- | --- | --- | --- | --- | --- |
| M_187 | Hirsutaside A | 70.34 | 0.34 | 0.81 | -0.10 | 3.23 | -4.85 | *U. rhynchophylla* |
| M_188 | 4-hydroxybenzyl alcohol | 55.21 | 0.60 | 0.02 | 0.34 | 0.53 | -0.32 | *G. elata* |
| M_189 | Isoferulic acid | 67.69 | 0.50 | 0.06 | 0.08 | 1.56 | -2.35 | *S. miltiorrhiza* |
| M_190 | Bilobalide | 86.51 | -0.78 | 0.36 | -1.34 | 0.16 | -1.50 | *G. biloba* |

***molecule after deglycosylation**

Table S3 The detailed information of docking validation.

| Compound | Target | GoldScore | Source | Compound | Target | GoldScore | Source |
| --- | --- | --- | --- | --- | --- | --- | --- |
| M_001 | NOS2 | 42.78 | LTC | M_074 | MAOA | 64.88 | SEA |
| M_003 | NOS3 | 44.79 | LTC | M_075 | PTGS2 | 46.76 | LTC |
| M_003 | ADRA1D | 50.21 | LTC | M_078 | PTGS2 | 48.59 | LTC |
| M_004 | NOS2 | 45.46 | LTC | M_079 | XDH | 49.00 | SEA |
| M_004 | GSK3B | 51.47 | LTC | M_080 | F2 | 42.98 | LTC |
| M_005 | PTGS1 | 40.99 | LTC | M_080 | NOS3 | 46.52 | LTC |
| M_006 | PTGS1 | 40.39 | LTC | M_082 | XDH | 49.99 | SEA |
| M_007 | PTGS2 | 41.14 | LTC | M_083 | PTGS2 | 49.22 | LTC |
| M_007 | F7 | 47.41 | LTC | M_083 | ALOX5 | 55.37 | SEA |
| M_007 | PYGM | 50.58 | LTC | M_084 | XDH | 52.72 | SEA |
| M_009 | PTGS2 | 49.97 | LTC | M_086 | PTGS2 | 44.07 | LTC |
| M_010 | PTGS2 | 57.86 | LTC | M_087 | XDH | 52.63 | SEA |
| M_011 | HMGCR | 43.08 | LTC | M_089 | PTGS2 | 42.65 | LTC |
| M_011 | TGFBR1 | 45.31 | LTC | M_089 | NOS2 | 53.01 | LTC |
| M_011 | PPARG | 46.27 | LTC | M_090 | XDH | 47.88 | SEA |
| M_011 | PPARD | 50.50 | LTC | M_091 | MAPK14 | 43.53 | LTC |
| M_011 | PLA2G2A | 51.84 | LTC | M_093 | MAPK14 | 52.66 | LTC |
| M_012 | PTGS2 | 41.02 | LTC | M_094 | PTGS2 | 49.46 | LTC |
| M_013 | NR3C1 | 46.54 | LTC | M_094 | ALOX5 | 53.83 | SEA |
| M_013 | HMGCR | 49.37 | LTC | M_094 | GSK3B | 54.24 | LTC |
| M_013 | PPARD | 55.16 | LTC | M_095 | ALOX5 | 50.90 | SEA |
| M_014 | BCL2L1 | 42.22 | STITCH | M_095 | PTGS2 | 57.02 | LTC |
| M_014 | SELE | 51.77 | LTC | M_096 | XDH | 51.93 | SEA |
| M_014 | KDR | 54.35 | LTC | M_097 | GSK3B | 46.77 | LTC |
| M_014 | CTSK | 56.08 | LTC | M_097 | PTGS2 | 50.92 | LTC |
| M_014 | F7 | 63.72 | LTC | M_097 | NOS2 | 56.06 | LTC |
| M_014 | BAX | 65.50 | STITCH | M_098 | MAOB | 61.30 | SEA |
| M_014 | HMGCR | 68.30 | LTC | M_099 | PTGS2 | 46.28 | LTC |
| M_014 | PLA2G2A | 68.77 | LTC | M_100 | PTGS2 | 47.19 | LTC |
| M_014 | MMP9 | 73.05 | STITCH | M_101 | XDH | 47.11 | SEA |
| M_014 | F2 | 79.68 | LTC | M_102 | PTGS2 | 53.79 | LTC |
| M_014 | PPARG | 92.31 | LTC | M_103 | XDH | 52.27 | SEA |
| M_015 | F2 | 53.91 | LTC | M_104 | MAPK14 | 40.01 | LTC |
| M_016 | F10 | 43.13 | LTC | M_104 | PTGS2 | 48.12 | LTC |
| M_016 | F2 | 46.61 | LTC | M_105 | PTGS2 | 41.60 | LTC |
| M_016 | CTSK | 46.96 | LTC | M_105 | NOS2 | 58.63 | LTC |
| M_016 | PPARD | 47.73 | LTC | M_106 | XDH | 45.16 | SEA |
| M_016 | HMGCR | 48.49 | LTC | M_107 | GSK3B | 42.52 | LTC |
| M_016 | TGFBR1 | 48.53 | LTC | M_107 | PTGS2 | 51.67 | LTC |
| M_016 | KDR | 48.56 | LTC | M_108 | NOS2 | 52.33 | LTC |
| M_016 | NR3C1 | 56.34 | LTC | M_108 | PTGS2 | 52.47 | LTC |
| M_017 | CTSK | 45.29 | LTC | M_108 | ALOX5 | 56.82 | SEA |

Table S3: Continued

| Compound | Target | GoldScore | Source | Compound | Target | GoldScore | Source |
| --- | --- | --- | --- | --- | --- | --- | --- |
| M_017 | PPARD | 45.57 | LTC | M_109 | PTGS2 | 41.97 | LTC |
| M_017 | TGFBR1 | 48.33 | LTC | M_109 | NOS2 | 51.29 | LTC |
| M_017 | PLA2G2A | 48.91 | LTC | M_110 | PTGS2 | 47.24 | LTC |
| M_017 | HMGCR | 50.26 | LTC | M_110 | NOS2 | 47.82 | LTC |
| M_017 | PTGS2 | 51.52 | LTC | M_111 | PTGS2 | 51.14 | LTC |
| M_017 | MMP9 | 51.83 | LTC | M_111 | ALOX5 | 55.11 | SEA |
| M_017 | F2 | 51.84 | LTC | M_112 | GSK3B | 40.62 | LTC |
| M_017 | NR3C1 | 54.24 | LTC | M_112 | PTGS2 | 45.67 | LTC |
| M_017 | NR1H3 | 62.27 | LTC | M_112 | NOS2 | 46.90 | LTC |
| M_018 | NR3C1 | 51.43 | LTC | M_112 | MAOB | 55.00 | SEA |
| M_018 | PPARD | 57.31 | LTC | M_113 | PTGS2 | 49.88 | LTC |
| M_018 | NR1H3 | 62.20 | LTC | M_114 | PTGS2 | 44.02 | LTC |
| M_019 | PTGS2 | 40.35 | LTC | M_115 | GSK3B | 48.40 | LTC |
| M_019 | GSK3B | 44.32 | LTC | M_115 | ALOX5 | 59.51 | SEA |
| M_020 | CASP3 | 40.50 | LTC | M_116 | PTGS2 | 47.75 | LTC |
| M_020 | PTGS2 | 40.73 | LTC | M_116 | GSK3B | 48.85 | LTC |
| M_020 | PPARG | 45.92 | LTC | M_116 | NOS2 | 55.34 | LTC |
| M_020 | PPARD | 50.90 | LTC | M_116 | ALOX5 | 59.58 | SEA |
| M_020 | PPARA | 51.01 | LTC | M_117 | PTGS2 | 46.28 | LTC |
| M_020 | PLA2G2A | 51.99 | LTC | M_118 | PTGS2 | 53.35 | LTC |
| M_020 | NR1H3 | 65.43 | LTC | M_118 | ALOX5 | 58.92 | SEA |
| M_021 | PPARG | 45.10 | LTC | M_119 | PTGS2 | 41.49 | LTC |
| M_021 | TGFBR1 | 46.37 | LTC | M_119 | NOS3 | 49.03 | LTC |
| M_021 | NR3C1 | 48.96 | LTC | M_123 | PTGS1 | 40.84 | LTC |
| M_021 | PLA2G2A | 52.24 | LTC | M_123 | ACHE | 53.04 | LTC |
| M_021 | PPARD | 58.05 | LTC | M_124 | DRD2 | 41.36 | LTC |
| M_021 | NR1H3 | 59.18 | LTC | M_124 | MAPK14 | 45.14 | LTC |
| M_022 | PTGS2 | 41.08 | LTC | M_124 | PTGS1 | 45.67 | LTC |
| M_022 | TGFBR1 | 46.32 | LTC | M_124 | PTGS2 | 46.28 | LTC |
| M_022 | PLA2G2A | 51.37 | LTC | M_124 | PTPN1 | 47.64 | LTC |
| M_022 | PPARD | 56.95 | LTC | M_124 | GSK3B | 49.07 | LTC |
| M_022 | NR1H3 | 57.62 | LTC | M_124 | MMP3 | 51.78 | LTC |
| M_023 | PTGS2 | 40.66 | LTC | M_124 | NOS3 | 52.47 | LTC |
| M_023 | HMGCR | 43.29 | LTC | M_124 | ACHE | 53.33 | LTC |
| M_023 | PPARG | 50.00 | LTC | M_124 | F2 | 62.52 | LTC |
| M_023 | NR1H3 | 62.03 | LTC | M_124 | HRH1 | 63.42 | LTC |
| M_024 | PTGS2 | 43.17 | LTC | M_125 | MAPK14 | 42.44 | LTC |
| M_025 | F10 | 40.10 | LTC | M_125 | PTGS2 | 43.74 | LTC |
| M_025 | NR3C1 | 42.09 | LTC | M_125 | DRD2 | 45.20 | LTC |
| M_025 | THRB | 43.81 | SEA | M_125 | MMP3 | 52.43 | LTC |
| M_025 | F7 | 47.16 | LTC | M_125 | NOS3 | 52.49 | LTC |
| M_025 | KDR | 50.38 | LTC | M_125 | PTPN1 | 54.09 | LTC |

Table S3: Continued

| Compound | Target | GoldScore | Source | Compound | Target | GoldScore | Source |
| --- | --- | --- | --- | --- | --- | --- | --- |
| M_026 | F10 | 40.83 | LTC | M_125 | PTGS1 | 54.27 | LTC |
| M_026 | TGFBR1 | 51.81 | LTC | M_125 | GSK3B | 55.13 | LTC |
| M_026 | NR3C1 | 52.95 | LTC | M_125 | ACHE | 57.55 | LTC |
| M_026 | F7 | 57.83 | LTC | M_125 | HRH1 | 59.89 | LTC |
| M_026 | PLA2G2A | 67.94 | LTC | M_126 | PTGS1 | 40.64 | LTC |
| M_027 | NR3C1 | 48.67 | LTC | M_126 | ACHE | 41.08 | LTC |
| M_027 | PLA2G2A | 51.18 | LTC | M_127 | ACHE | 40.08 | LTC |
| M_028 | F2 | 43.73 | LTC | M_128 | PTPN1 | 41.59 | LTC |
| M_028 | TGFBR1 | 45.08 | LTC | M_128 | AKT1 | 45.17 | LTC |
| M_028 | NR3C1 | 48.52 | LTC | M_128 | PARP1 | 46.85 | LTC |
| M_028 | PPARA | 51.01 | LTC | M_128 | PTGS1 | 50.36 | LTC |
| M_028 | PLA2G2A | 53.08 | LTC | M_128 | PIK3CG | 58.20 | LTC |
| M_028 | PPARD | 58.46 | LTC | M_128 | GSK3B | 58.35 | LTC |
| M_029 | TGFBR1 | 47.77 | LTC | M_129 | GABRA5 | 41.50 | LTC |
| M_029 | PLA2G2A | 52.19 | LTC | M_129 | MAPK14 | 44.98 | LTC |
| M_029 | PPARD | 54.15 | LTC | M_129 | PTGS1 | 45.13 | LTC |
| M_030 | PTGS2 | 40.11 | LTC | M_129 | HTR2A | 45.57 | LTC |
| M_031 | XDH | 73.38 | SEA | M_129 | DRD2 | 46.85 | LTC |
| M_032 | NOS3 | 44.63 | LTC | M_129 | GSK3B | 47.41 | LTC |
| M_032 | NOS2 | 50.45 | LTC | M_129 | PTGS2 | 52.58 | LTC |
| M_033 | PTGS2 | 40.24 | LTC | M_129 | MMP3 | 53.73 | LTC |
| M_033 | NOS3 | 45.27 | LTC | M_129 | ACHE | 54.30 | LTC |
| M_033 | NOS2 | 47.00 | LTC | M_129 | NOS3 | 55.71 | LTC |
| M_034 | PTGS2 | 49.90 | LTC | M_129 | PTPN1 | 56.41 | LTC |
| M_035 | XDH | 51.49 | SEA | M_129 | HRH1 | 67.38 | LTC |
| M_037 | XDH | 53.29 | SEA | M_130 | PTGS2 | 50.13 | LTC |
| M_037 | ALOX5 | 54.09 | SEA | M_132 | NOS3 | 47.40 | LTC |
| M_037 | MAOA | 63.77 | SEA | M_132 | F2 | 47.47 | LTC |
| M_038 | PTGS2 | 44.49 | LTC | M_133 | NOS2 | 49.94 | LTC |
| M_039 | ALOX5 | 51.46 | SEA | M_134 | F10 | 40.85 | LTC |
| M_040 | XDH | 50.34 | SEA | M_134 | PTGS2 | 59.86 | LTC |
| M_040 | MAOA | 62.51 | SEA | M_134 | NOS2 | 60.22 | LTC |
| M_041 | XDH | 49.72 | SEA | M_134 | F2 | 63.32 | LTC |
| M_042 | NOS2 | 41.80 | LTC | M_135 | PTGS2 | 40.02 | LTC |
| M_043 | F10 | 42.88 | LTC | M_136 | SELP | 53.71 | SEA |
| M_043 | PTGS2 | 45.12 | LTC | M_136 | FABP4 | 57.76 | SEA |
| M_043 | F2 | 65.58 | LTC | M_137 | PLA2G2A | 41.34 | LTC |
| M_043 | NOS2 | 68.94 | LTC | M_137 | HMGCR | 42.66 | LTC |
| M_044 | GSK3B | 47.18 | LTC | M_137 | KDR | 46.07 | LTC |
| M_044 | NOS2 | 54.32 | LTC | M_137 | CASP3 | 52.75 | LTC |
| M_044 | F2 | 61.68 | LTC | M_137 | F2 | 58.98 | LTC |
| M_045 | PTGS2 | 40.26 | LTC | M_143 | PTGS2 | 43.86 | LTC |

Table S3: Continued

| Compound | Target | GoldScore | Source | Compound | Target | GoldScore | Source |
| --- | --- | --- | --- | --- | --- | --- | --- |
| M_045 | NOS2 | 54.94 | LTC | M_144 | KDR | 40.01 | LTC |
| M_046 | PTGS2 | 47.03 | LTC | M_144 | HMGCR | 41.09 | LTC |
| M_046 | NOS3 | 51.93 | STITCH | M_144 | F2 | 44.25 | LTC |
| M_047 | PTGS2 | 44.64 | LTC | M_144 | PPARG | 58.98 | LTC |
| M_047 | GSK3B | 46.88 | LTC | M_145 | KDR | 41.89 | LTC |
| M_047 | F2 | 56.92 | LTC | M_145 | NR1H3 | 42.30 | LTC |
| M_048 | PTGS2 | 40.26 | LTC | M_145 | NR3C1 | 45.50 | LTC |
| M_048 | GSK3B | 47.81 | LTC | M_145 | F2 | 49.50 | LTC |
| M_048 | NOS2 | 54.23 | LTC | M_145 | CASP3 | 49.65 | LTC |
| M_049 | F2 | 58.13 | LTC | M_145 | PPARG | 55.27 | LTC |
| M_050 | BCL2 | 43.25 | STITCH | M_146 | ALOX5 | 41.58 | SEA |
| M_050 | F2 | 62.31 | LTC | M_148 | BCL2 | 40.76 | STITCH |
| M_051 | F2 | 58.21 | LTC | M_149 | NOS2 | 47.12 | LTC |
| M_052 | PTGS2 | 40.58 | LTC | M_149 | PTGS2 | 47.37 | LTC |
| M_052 | F2 | 59.99 | LTC | M_149 | NOS3 | 48.27 | LTC |
| M_053 | F2 | 58.39 | LTC | M_149 | F2 | 58.04 | LTC |
| M_054 | F2 | 59.05 | LTC | M_151 | ALOX5 | 53.17 | SEA |
| M_055 | F2 | 64.00 | LTC | M_152 | ALOX5 | 53.10 | SEA |
| M_057 | F2 | 65.39 | LTC | M_152 | XDH | 55.91 | SEA |
| M_058 | F2 | 63.37 | LTC | M_156 | PTGS2 | 43.42 | LTC |
| M_059 | PTGS2 | 41.50 | LTC | M_157 | MAOA | 66.98 | SEA |
| M_059 | F2 | 62.58 | LTC | M_158 | XDH | 52.35 | SEA |
| M_061 | GSK3B | 48.01 | LTC | M_158 | ALOX5 | 56.89 | SEA |
| M_061 | F2 | 56.69 | LTC | M_159 | XDH | 53.62 | SEA |
| M_061 | NOS2 | 59.64 | LTC | M_160 | ALOX12 | 46.71 | SEA |
| M_062 | HTR1D | 42.33 | STITCH | M_160 | AKT1 | 48.94 | STITCH |
| M_062 | F2 | 57.21 | LTC | M_160 | INSR | 52.89 | STITCH |
| M_062 | ADRA1D | 60.46 | STITCH | M_161 | ODC1 | 56.21 | SEA |
| M_063 | F2 | 54.93 | LTC | M_162 | ESR1 | 50.21 | LTC |
| M_064 | F2 | 52.99 | LTC | M_163 | ESR1 | 44.80 | LTC |
| M_065 | NOS2 | 54.70 | LTC | M_164 | NR3C2 | 51.30 | LTC |
| M_065 | F2 | 61.79 | LTC | M_165 | NR3C2 | 45.93 | LTC |
| M_068 | PTGS2 | 40.16 | LTC | M_166 | CES1 | 42.61 | SEA |
| M_069 | F2 | 65.22 | LTC | M_167 | MIF | 46.53 | SEA |
| M_070 | GSK3B | 49.22 | LTC | M_168 | ESR1 | 56.15 | LTC |
| M_070 | NOS2 | 52.49 | LTC | M_169 | PTGS1 | 40.43 | LTC |
| M_070 | F2 | 52.65 | LTC | M_169 | GSK3B | 42.04 | LTC |
| M_071 | XDH | 50.59 | SEA | M_169 | PTGS2 | 42.37 | LTC |
| M_071 | MAOB | 57.99 | SEA | M_169 | ADRA1D | 44.49 | LTC |
| M_071 | MAOA | 62.52 | SEA | M_169 | NOS3 | 53.36 | LTC |
| M_072 | TGFBR1 | 40.01 | LTC | M_170 | ESR1 | 40.19 | LTC |
| M_072 | HMGCR | 42.03 | LTC | M_170 | NOS2 | 47.52 | LTC |

Table S3: Continued

| Compound | Target | GoldScore | Source | Compound | Target | GoldScore | Source |
| --- | --- | --- | --- | --- | --- | --- | --- |
| M_072 | NOS2 | 44.50 | LTC | M_180 | GSK3B | 50.62 | LTC |
| M_072 | CASP3 | 58.46 | LTC | M_180 | ESR1 | 51.88 | LTC |
| M_073 | XDH | 45.73 | SEA | M_186 | PTGS2 | 53.64 | LTC |
| M_074 | MAOB | 58.55 | SEA | M_186 | ESR1 | 64.03 | LTC |
